# Multi-omics identification and functional validation of signal regulatory protein gamma as a prognostic biomarker and immune regulator in head and neck squamous cell carcinoma

**DOI:** 10.3389/fimmu.2026.1835156

**Published:** 2026-08-21

**Authors:** Jiaqi Tang, Yulun He, Yuqi Wang, Yanbing Yao, Mingqi Ma, Kunyu Chen, Xiaolong Zhang, Yun Li, Huan Li, Jianhua Wei

**Affiliations:** State Key Laboratory of Oral & Maxillofacial Reconstruction and Regeneration, National Clinical Research Center for Oral Diseases, Shaanxi Clinical Research Center for Oral Diseases, Department of Oral and Maxillofacial Surgery, School of Stomatology, The Fourth Military Medical University, Xi’an, Shaanxi, China

**Keywords:** head and neck squamous cell carcinoma, immune checkpoints, machine learning, SIRPG, tumor microenvironment

## Abstract

**Background:**

Head and neck squamous cell carcinoma (HNSCC) comprises biologically diverse tumors, and durable responses to immune-checkpoint blockade are achieved by only a subset of patients. There remains a need for markers that connect clinical outcome with malignant-cell phenotypes and tissue-level immune organization.

**Methods:**

We integrated The Cancer Genome Atlas HNSCC cohort (TCGA-HNSC), five Gene Expression Omnibus (GEO) validation cohorts, single-cell RNA sequencing, Visium spatial transcriptomics, cellular indexing of transcriptomes and epitopes by sequencing (CITE-seq)-informed protein-potential inference, pharmacogenomic screening, genetic-risk analysis and experimental validation. A reconstructed 296-pipeline survival modelling framework was used to prioritize prognostic hub genes across validation-cohort-specific analyses.

**Results:**

SIRPG was repeatedly ranked among the top ten selected genes in all five validation cohorts. At single-cell resolution, SIRPG-high tumor cells showed stronger malignant-cell features, immune-inhibitory and metabolic programs, Scissor-positive risk association, CLCA2/P53-related perturbation signals and inferred SIRPG-CD47/signal regulatory protein (SIRP) communication. Spatial analyses placed this axis within an immune-checkpoint-coupled niche, supported by Maxspin/multiview intercellular spatial modelling (MISTy) spatial coupling, communication analysis by optimal transport (COMMOT)-inferred CD47-SIRPG communication and scProTrans-inferred CD47/SIRPG protein-potential overlap. Functionally, SIRPG knockdown reduced HNSCC cell viability and increased apoptosis, whereas re-expression of short hairpin RNA (shRNA)-resistant SIRPG restored the CLCA2-BAX/BCL2 protein response.

**Conclusion:**

Together, these findings identify SIRPG as an immune-related prognostic hub and context-dependent tumor-cell regulator associated with apoptosis, immune communication and spatial microenvironmental organization in HNSCC.

## Introduction

Head and neck squamous cell carcinoma (HNSCC) is an aggressive epithelial cancer arising across the oral cavity, pharynx and larynx. Approximately 900,000 new cases are diagnosed worldwide each year (1). Despite advances in surgical techniques, precision radiotherapy, and immune checkpoint blockade (ICB) therapy, the 5-year survival rate for people with advanced HNSCC has remained at approximately 40%–50% (2). Adverse outcomes reflect early recurrence, marked intratumoral diversity and resistance that may be present at treatment initiation or emerge during therapy (3).

**Figure 1:**
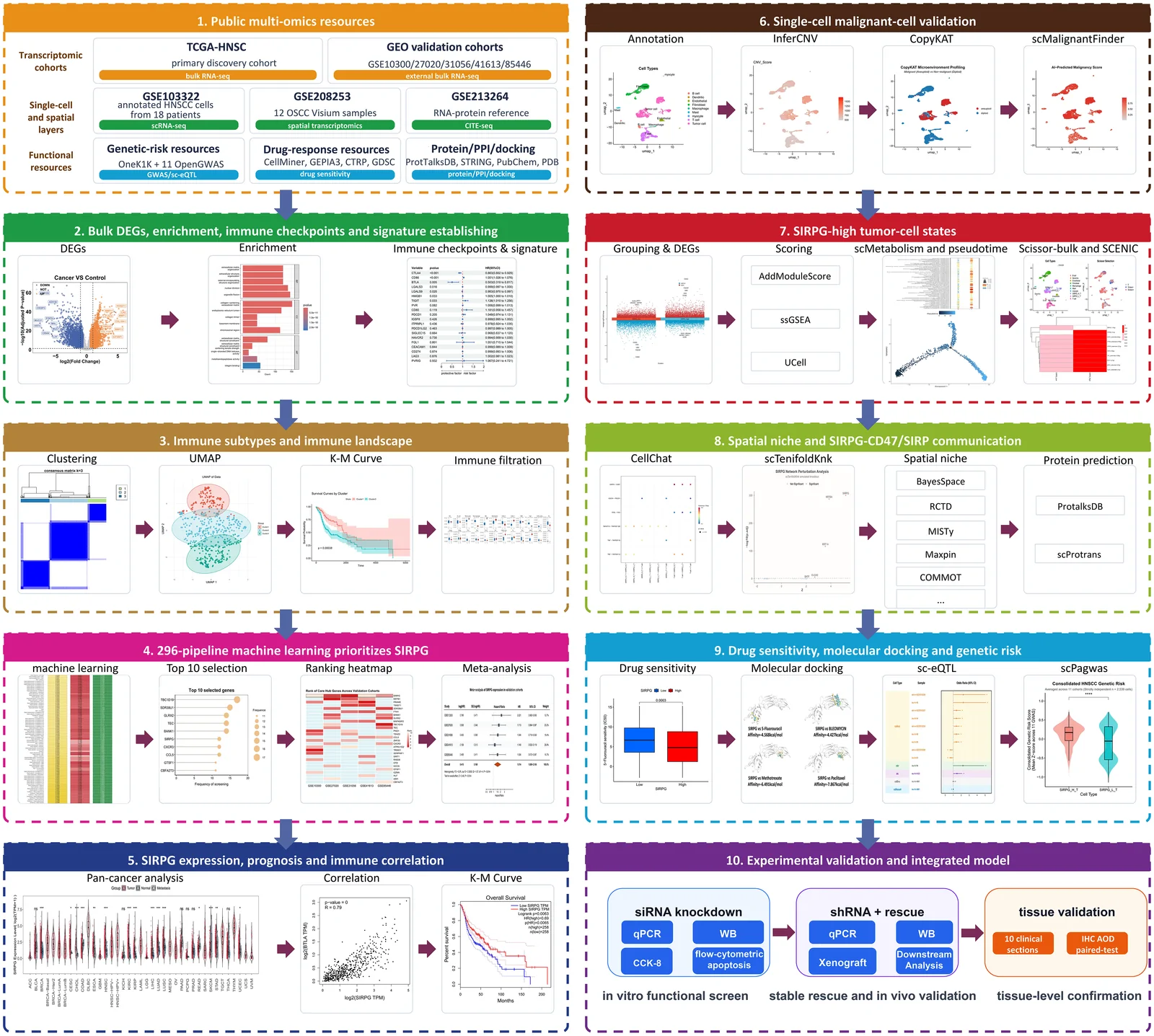
Illustration of the workflow and an overview of approaches and resources used.

Antibodies directed against programmed cell death protein 1 (PD-1) or programmed death ligand 1 (PD-L1) have changed first-line management for recurrent or metastatic HNSCC. Approximately 15%-20% of patients derive durable benefit (4). Nevertheless, many patients do not respond initially, reflecting the complexity of the tumor microenvironment (5). This immune escape mechanism involves not only the depletion of cluster of differentiation 8-positive (CD8+) T cells and the amplification of regulatory T cells, but is also closely linked to the compensatory upregulation of alternative immune checkpoints, including T-cell immunoreceptor with immunoglobulin and ITIM domains (TIGIT) and lymphocyte-activation gene 3 (LAG-3) (6). Because PD-L1 alone cannot represent the dynamic and heterogeneous immune state of HNSCC, a broader mapping of immune-related molecular alterations is required to identify markers linked to prognosis and microenvironmental context. Such biomarkers could improve patient stratification and guide immunotherapy-oriented studies. To address this question, we designed a multi-layered analytical framework. First, we characterized the immune landscape of HNSCC using the TCGA cohort. Next, a reconstructed 296-pipeline survival machine-learning framework was applied to identify reproducible prognostic hub genes across multiple independent GEO cohorts. Among the prioritized candidates, SIRPG exhibited repeated cross-cohort prioritization and was therefore selected for subsequent investigation. We then integrated four complementary evidence layers, including bulk transcriptomic cohorts, single-cell RNA-seq, Visium spatial transcriptomics and CITE-seq-derived RNA-protein translation information for protein-potential inference. We then integrated this framework with *in vitro* knockdown and rescue assays, pharmacogenomic prediction, molecular docking, single-cell Mendelian randomization and scPagwas analysis to define the prognostic, cellular, spatial and functional context of SIRPG in HNSCC (**Figure 1**).

## Materials and methods

### Data acquisition and processing

We obtained TCGA-HNSC transcriptomic profiles and matched clinical annotations through UCSC Xena. For discovery analyses, only primary tumors coded 01A and normal tissues coded 10A were retained. External validation and cross-cohort modelling were performed in five GEO series: GSE10300, GSE27020, GSE31056, GSE41613 and GSE85446. Where count-scale data were available, expression values were converted to TPM and then log2(TPM + 1) transformed. Pharmacogenomic analyses used CellMiner, CREAMMIST and GEPIA3-based drug-sensitivity resources integrating Cancer Therapeutics Response Portal (CTRP), Genomics of Drug Sensitivity in Cancer 1 (GDSC1) and Genomics of Drug Sensitivity in Cancer 2 (GDSC2) information. Single-cell expression quantitative trait loci (sc-eQTL) data were obtained from the OneK1K cohort, and 11 OpenGWAS outcomes related to HNSCC, oral cavity cancer or oropharyngeal cancer were screened for single-cell eQTL (sc-eQTL) Mendelian randomization analyses (7). The annotated HNSCC scRNA-seq dataset GSE103322, generated from 18 oral-cavity tumor samples, was used to examine SIRPG expression, checkpoint association, malignant-cell identity and SIRPG-defined tumor-cell states at single-cell resolution. For scPagwas-based genetic-risk projection, 11 HNSCC-related genome-wide association study (GWAS) datasets were retained after phenotype matching. We also analyzed the public OSCC Visium dataset GSE208253, which includes 12 spatial sections with published tumor-core, transitory-region and leading-edge labels, to evaluate tissue-level organization of SIRPG-associated programs (8). For scProTrans-based protein-potential inference, the CITE-seq dataset GSE213264 was used as an RNA-protein reference layer to support RNA-to-protein translation rather than as an independent HNSCC biological cohort.

### Differential expression, enrichment analysis, and identification of immune subtypes

DESeq2 was applied to TCGA-HNSC to identify differentially expressed genes (threshold: adjusted P < 0.05 and absolute log2 fold change (|log2FC|) > 1). clusterProfiler was used for Gene Ontology (GO), Kyoto Encyclopedia of Genes and Genomes (KEGG) and Gene Set Enrichment Analysis (GSEA; c5.go and c2.cp reference sets).

For representative immune-checkpoint genes, including PDCD1 and CTLA4, expression differences were tested with Wilcoxon analysis, and associations with overall survival (OS) and progression-free survival (PFS) were evaluated by univariable Cox regression. Related genes were then retrieved from the GeneCards database (protein coding, relevance score > 10). Totally, 28 immune-related feature genes were identified.

Based on these 28 genes, unsupervised clustering (k-means, Euclidean distance, sampling rate of 0.8, 1000 replicates, Uniform Manifold Approximation and Projection (UMAP) visualization) was performed on the TCGA-HNSC cohort using ConsensusClusterPlus to identify immune subtypes. Immune-cell infiltration in each subtype was estimated using the ImmuCellAI platform. Survival differences between subtypes were evaluated using Kaplan-Meier (K-M) curves and log-rank tests.

### Reconstructed 296-pipeline survival machine-learning framework

To prioritize stable prognostic features, prognosis-related genes derived from immune-subtype-associated differentially expressed genes (DEGs) were intersected with the expression matrices of TCGA-HNSC and each validation cohort before model fitting. The TCGA-HNSC cohort was used as the training cohort, and five GEO datasets were used as independent validation cohorts: GSE10300, GSE27020, GSE31056, GSE41613 and GSE85446. The validation cohorts contained 44 samples in GSE10300, 109 samples in GSE27020, 96 samples in GSE31056, 97 samples in GSE41613 and 66 samples in GSE85446.

Following a reported large-scale selector-learner survival-modelling strategy (9), we reconstructed a 296-pipeline framework in R language. The final grid contained 21 stand-alone survival models and 275 two-stage selector-learner pipelines generated from 11 feature selectors and 25 survival learners.

Feature selection was performed in the TCGA-HNSC training cohort and the selected features were then passed to the corresponding survival learner. The fitted learner generated risk scores for both the training cohort and the paired validation cohort. Model-derived risk scores were assessed by the concordance index (C-index).

For each validation-cohort-specific run, genes were ranked by integrating selector frequency and recurrence across the 296 fitted pipelines. The top-ranked genes were visualized as cohort-specific Top10 selected-gene plots, and SIRPG ranking across cohorts was summarized in the core hub gene rank heatmap.

### Analysis of the *SIRPG* gene

The expression of SIRPG in pan-cancer and its correlation with immune checkpoints were assessed using TIMER3 and Spearman’s correlation (10). The prognostic value of SIRPG for OS and disease-free survival (DFS) was assessed using Gene Expression Profiling Interactive Analysis 2 (GEPIA2) (11). Regarding drug sensitivity, U.S. Food and Drug Administration (FDA)-approved agents and drugs in clinical trials were first screened using CellMiner and CREAMMIST, and significant drug-gene pairings were defined as |R| > 0.5 and P < 0.05. Predicted drug sensitivity in HNSCC was further evaluated using Gene Expression Profiling Interactive Analysis 3 (GEPIA3) (12) resources integrating CTRP, GDSC1 and GDSC2, with lower predicted half-maximal inhibitory concentration (IC50) interpreted as greater predicted sensitivity.

### Single-cell RNA-seq preprocessing and annotation

The GSE103322 scRNA-seq matrix was processed in R v4.5.1 with a Seurat-based workflow. Cell identities supplied by the original study were kept for downstream comparisons. The first 40 principal components were used for dimensionality reduction and clustering, and the resolution parameter was set to 0.4. UMAP plots were generated to inspect annotated cell populations, sample distribution and cell-cycle phase. SIRPG and inhibitory checkpoint genes were visualized across the atlas, and pairwise correlations were calculated as supporting analyses.

### Malignant-cell validation and SIRPG stratification

Transcriptome-inferred copy-number variation (CNV) scores inferred by inferCNV were calculated to support malignant-cell identity across annotated cell types (13). CopyKAT was used as an independent aneuploidy-based malignant-cell classifier (14), and scMalignantFinder was used to estimate malignant probability at the single-cell level using a machine-learning classifier trained on pan-cancer malignant-cell signatures (15). Concordance among inferCNV-related CNV scoring, CopyKAT classification and scMalignantFinder probability was used to support the annotated tumor-cell compartment. Tumor cells were then extracted and divided into SIRPG-high and SIRPG-low groups using the median SIRPG expression value as the cutoff after MAGIC-based dropout correction (16).

### Functional scoring and prognostic projection

SIRPG-high and SIRPG-low tumor cells were compared for differential expression within the Seurat workflow. GO and KEGG enrichment analyses were performed using clusterProfiler. Immune-inhibitory programs were quantified using AddModuleScore, single-sample gene set enrichment analysis (ssGSEA) and UCell; agreement between scoring approaches was evaluated by pairwise correlation. Metabolic pathway activity was estimated using scMetabolism with Reactome pathway gene sets (17).

Scissor linked tumor-cell states from the single-cell data to TCGA-HNSC bulk risk information (18). Scissor-positive marker genes were intersected with survival-associated prognostic genes to define a 17-gene Scissor-positive survival-related signature. In independent bulk cohorts, ssGSEA was used to score this signature, and Kaplan-Meier analysis tested its survival-stratification ability. Time-dependent receiver operating characteristic (ROC) analyses were then used to compare the signature with clinical variables.

### Pseudotime and virtual perturbation analyses

CytoTRACE2 was used to estimate tumor-cell differentiation potential (19). A monocle2/DDRTree workflow ordered tumor cells along pseudotime, after which selected genes were plotted across the inferred trajectory (20). Virtual SIRPG knockout was performed using scTenifoldKnk to identify perturbation-sensitive genes and network responses after simulated SIRPG depletion. Candidate mechanism genes were nominated from the overlap among SIRPG-associated DEGs, genes affected by virtual knockout and HALLMARK P53 pathway members.

### Genetic-risk projection and cell-cell communication

To examine inherited-susceptibility signals at single-cell resolution, scPagwas projected 11 HNSCC-related GWAS datasets (21) onto SIRPG-stratified tumor cells. scPagwas generated a transcriptional risk score (TRS) for each cell, and these values were summarized as a consolidated HNSCC genetic-risk score after within-GWAS standardization and cell-level averaging.

Transcriptional regulatory activity was analyzed with Single-Cell rEgulatory Network Inference and Clustering (SCENIC) (22). Cell-cell communication was inferred using CellChat (23), with focused analysis of the SIRP signaling pathway and the SIRPG-CD47 ligand-receptor pair.

### Spatial transcriptomics, CITE-seq-informed protein-potential inference and clinical proteome-context analyses

Neighbor-purity analysis and BayesSpace clustering were used to evaluate whether the published tumor-region labels were spatially coherent (24). For each Visium spot, neighbor purity represented the proportion of adjacent spots carrying the same published region label. Observed values were compared against label-permuted controls to determine whether tumor core, transitory and leading-edge regions formed coherent domains. BayesSpace was additionally applied as an independent spatial clustering method, and the resulting clusters were mapped back to the published tumor regions.

To characterize the SIRPG-associated spatial niche, SIRPG expression, immune-checkpoint signatures and regional marker programs were projected onto spatial sections. Maxspin, or maximization of spatial information, was used to quantify pairwise spatial coupling between SIRPG and checkpoint-related features using an information-theoretic framework (25). MISTy multiview modelling was further used to evaluate intra-spot and neighboring-region predictors of SIRPG expression, enabling separation of local molecular associations from spatial-context effects (26). Robust Cell Type Decomposition (RCTD) was used to estimate cell-type proportions in spatial spots using the matched single-cell reference (27). To evaluate deconvolution quality, spots with a dominant RCTD cell-type weight greater than 0.6 were defined as high-confidence assignments, and both these assignments and the second-largest cell-type weight were visualized across tissue sections. The second-largest weight was interpreted as a proxy for spot-level compositional mixing.

COMMOT was used to infer spatial ligand-receptor communication (28), focusing on the CD47-SIRPG axis. Communication direction was visualized on the histological image and within the published spatial-region framework to evaluate whether the inferred CD47-SIRPG signal showed regional organization across tumor core, transitory and leading-edge domains.

ScProTrans was used to infer spatial protein-potential maps for CD47 and SIRPG from Visium spatial transcriptomic profiles using RNA-protein translation information learned from paired CITE-seq reference data (29). In this analysis, GSE213264 provided CITE-seq data, a paired RNA and protein reference layer, whereas GSE208253 provided the target spatial transcriptomic profiles. Predicted CD47 and SIRPG protein-potential scores were visualized on tissue sections, and their spatial overlap was used as a transcriptome-derived approximation of protein-level co-localization. These predictions were interpreted as inferred protein potential rather than measured spatial proteomics.

For external clinical proteomic context, ProtTalksDB was queried for SIRPG and selected pathway-relevant proteins, including CLCA2, BAX, BCL2 and immune-checkpoint-related proteins (30). These analyses were used as external proteome-context evidence.

### Single-cell eQTL analysis

To evaluate genetic regulation of SIRPG across cell types, cis-eQTL data of 14 immune cell types from the OneK1K cohort (7) were extracted as exposures. Instrumental variables (IVs) were selected using P < 0.05, linkage disequilibrium r² < 0.001 within a 100-kb window and F-statistic > 10. OpenGWAS outcomes related to HNSCC, oral cavity cancer and oropharyngeal cancer were harmonized using TwoSampleMR, and palindromic single nucleotide polymorphisms (SNPs) were removed. Steiger filtering was applied to test directionality. Inverse-variance weighting or Wald ratio analysis for single-IV instruments was used as the primary method, with Mendelian randomization-Egger (MR-Egger) and additional sensitivity methods used as supporting analyses.

### Molecular docking analysis

Molecular docking was performed using a conventional non-covalent docking workflow. The three-dimensional structure of SIRPG was prepared by removing solvent molecules and non-standard residues, followed by hydrogen addition and energy minimization when applicable. The three-dimensional structures of 5-Fluorouracil, Bleomycin, Methotrexate, and Paclitaxel were obtained from PubChem (https://pubchem.ncbi.nlm.nih.gov/) and the Protein Data Bank (PDB; https://www.rcsb.org/). Docking was performed between SIRPG and each ligand, whose conformations were visualized using PyMOL.

### Cell lines and culture conditions

SCC9 and CAL27 tongue squamous carcinoma cells and human oral keratinocytes were obtained from Wuhan Procell Biotechnology Co., Ltd. (Wuhan, China). Cells were maintained in Dulbecco’s modified Eagle medium/nutrient mixture F-12 (DMEM/F12; Gibco, Thermo Fisher Scientific, USA) supplemented with 10% fetal bovine serum (Gibco) and 1% penicillin-streptomycin (NCM Biotech). Cells were cultured in a humidified incubator at 37 °C and 5% CO_2_. The medium was replaced every 2–3 days, and cells were passaged when the confluence reached around 80%–90%.

### Small interfering RNA transfection

For transient SIRPG silencing, Shanghai GenePharma Co., Ltd. (Shanghai, China) synthesized threehuman SIRPG-targeting small interfering RNAs (siRNAs) and a non-targeting control (si-NC). SCC9 and CAL27 cells were plated in 6-well plates and transfected when they reached roughly 70% confluence. siRNA delivery was performed with Lipofectamine RNAiMAX (Thermo Fisher Scientific, USA) using the working conditions recommended by the manufacturer. Cells were collected 48 h later to assess knockdown efficiency. Western blotting was used to compare knockdown efficiency, after which si-SIRPG-321 was carried forward for Cell Counting Kit-8 (CCK-8) and apoptosis experiments. The siRNA sequences are listed in **Supplementary Table 1**.

### Stable shRNA-mediated SIRPG knockdown and rescue overexpression

Shanghai GenePharma Co., Ltd. provided three lentiviral shRNAs targeting SIRPG together with anon-targeting shRNA control (sh-NC). Each shRNA was carried by the LV-H1-ZsGreen1-T2A-Neo lentiviral vector at a titer of 10^8^ TU/mL. SCC9 and CAL27 cells were infected and selected following the supplier’s protocol. After quantitative real-time PCR (qRT-PCR) and western blot screening, sh-SIRPG-321 was chosen from the three candidates for functional assays and rescue experiments. The shRNA target sequences and vector information are listed in **Supplementary Table 1**.

For the rescue design, Shanghai GenePharma Co., Ltd. cloned an shRNA-resistant full-length human SIRPG coding sequence into a lentiviral overexpression vector. Three cell states were examined for each cell line: parental SCC9 or CAL27 control cells cultured under the same conditions without knockdown or rescue manipulation (SCC9-Control or CAL27-Control), stable sh-SIRPG cells, and sh-SIRPG + oe-SIRPG rescue cells generated by re-expressing shRNA-resistant SIRPG in the sh-SIRPG background. The non-targeting shRNA control described above was used during initial shRNA screening and was not the group labeled “Control” in the final three-group rescue experiment. SIRPG re-expression and the accompanying changes in CLCA2, BAX and BCL2 protein levels were assessed by western blotting.

### Subcutaneous xenograft assay

To test *in vivo* growth effects, nude-mouse subcutaneous xenografts were established using parental SCC9 or CAL27 control cells (SCC9-Control or CAL27-Control), stable sh-SIRPG cells or sh-SIRPG + oe-SIRPG rescue cells. Each cell-line experiment included three treatment groups with five mice in each group, resulting in 30 mice across the SCC9 and CAL27 experiments. The group size was selected to balance experimental comparability with the reduction principle of animal research; for each independent cell-line experiment, the three-group design with five mice per group yielded an error degrees of freedom of E = 15 − 3 = 12 under the resource equation approach (31). Tumor dimensions were measured at the indicated time points, and volume was calculated as length x width^2^/2. At the experimental endpoint (day 21 after cell inoculation), mice were euthanized by intraperitoneal injection of sodium pentobarbital (250 mg/kg). Following confirmation of death, the subcutaneous tumors were excised and photographed for gross comparison.

### Immunohistochemistry and image quantification

SIRPG IHC was performed on paraffin-embedded tissue sections. Sections were deparaffinized, rehydrated and subjected to heat-mediated antigen retrieval in ethylenediaminetetraacetic acid (EDTA) buffer (pH 8.0) for 30 min. Endogenous peroxidase activity was blocked with 3% methanol-hydrogen peroxide, and non-specific binding was blocked with 3% bovine serum albumin. Sections were incubated overnight at 4 °C with a rabbit anti-SIRPG primary antibody (PK94782S, Abmart, China, 1:100), followed by a horseradish peroxidase-conjugated goat anti-rabbit polymer secondary antibody (S-vision; G1302, Servicebio, China). Staining was visualized with 3,3′-diaminobenzidine, counterstained with hematoxylin, dehydrated, mounted and scanned.

For quantification, 10 sections containing tumor and adjacent normal regions were analyzed. In each section, three representative tumor fields and three adjacent-normal fields were selected and averaged separately. SIRPG staining was quantified as average optical density (AOD), defined as integrated optical density normalized to tissue area. Paired tumor and adjacent-normal AOD values were compared using a two-sided Wilcoxon signed-rank test.

### RNA extraction and qRT-PCR

Total RNA was isolated from cultured cells with a Rapid RNA Extraction Kit (M5105; NCM Biotech, Suzhou, China) following the supplied protocol. RNA concentration and purity were determined with a NanoDrop spectrophotometer (Thermo Fisher Scientific). Next, 1 µg of total RNA was reverse transcribed into complementary DNA (cDNA) using a PrimeScript™ RT Kit (with gDNA Eraser) (catalog number: RR037A; Takara, Tokyo, Japan).

qRT-PCR used TB Green Premix Ex Taq II (Takara) and a Bio-Rad CFX96 real-time PCR system(Bio-Rad, Hercules, CA, USA). The amplification program consisted of initial denaturation at 95 °C for 30 seconds, followed by 40 cycles of denaturation at 95 °C for 5 seconds and annealing/extension at 60 °C for 30 seconds. Relative SIRPG mRNA abundance was calculated by the comparative Ct method with GAPDH as the internal reference. Each reaction was run in triplicate. The primer sequences, siRNA sequences and lentiviral vector information used in this study are provided in **Supplementary Table 1**.

### Western blot

Cellular proteins were lysed in radioimmunoprecipitation assay (RIPA) buffer supplemented with protease inhibitor at 100:1 (NCM Biotech, China). Protein concentration was measured using a bicinchoninic acid (BCA) assay kit (NCM Biotech). Equal amounts of protein (20 μg) were separated by sodium dodecyl sulfate-polyacrylamide gel electrophoresis (SDS-PAGE) and transferred to 0.45-μm polyvinylidene difluoride (PVDF) membranes at 400 mA for 30 min. After blocking with 5% skim milk at room temperature for 2 h, membranes were incubated overnight at 4 °C with primary antibodies against SIRPG (1:1000, catalog number YT0733), BCL-2 (1:5000, catalog number YM8319), BAX (1:2000, catalog number YM8175), CLCA2 (1:500, catalog number YN6660) and GAPDH (1:10000, catalog number YM8394). All primary antibodies were purchased from Immunoway (China), and GAPDH served as the loading control. After TBST washing, membranes were incubated for 1 h with horseradish peroxidase-conjugated goat anti-rabbit immunoglobulin G (IgG; 1:5000). Chemiluminescent signals were developed with an enhanced chemiluminescence kit (NCM Biotech) and captured using a Bio-Rad imaging system. Band intensities were quantified using ImageJ and analyzed in R v4.5.1.

For the rescue western blots, each lane represented one biological replicate derived from a separately maintained culture flask (n = 3 independently cultured biological replicates per group), rather than a technical replicate. For each cell line, samples were loaded on a single gel in the order parental control replicates 1–3, molecular-weight marker, sh-SIRPG replicates 1–3, molecular-weight marker and sh-SIRPG + oe-SIRPG rescue replicates 1–3. All sample lanes were transferred together to one PVDF membrane. After transfer, the membrane was divided only horizontally according to molecular-weight range for antibody incubation and was not cut vertically by experimental group. Target proteins and GAPDH were detected from matched lane positions derived from the same transfer, and target-band intensity was normalized lane by lane to GAPDH.

### Cell viability test

Cell viability was measured with the Cell Counting Kit-8 assay. SCC9 and CAL27 cells were plated in 96-well plates at 1 x 10^4 cells per well. After attachment, wells were assigned to mock transfection, non-targeting siRNA control (si-NC) or SIRPG siRNA (si-SIRPG-321). Twenty-four hours after transfection, 20 uL CCK-8 reagent (NCM Biotech, China) was added per well, followed by a 2-h incubation at 37 °C. Absorbance was read at 450 nm on an Epoch microplate spectrophotometer (BioTek Instruments, USA). Each group contained six replicate wells. Cell viability was calculated as follows:

Cell viability (%) = (As − Ab)/(Ac − Ab) × 100%.

In this formula, As, Ac and Ab represent absorbance values for the si-SIRPG group, the control group (si-NC or mock) and blank medium, respectively.

### Flow cytometry for apoptosis

Forty-eight hours after si-SIRPG-321 or control transfection, apoptosis in SCC9 and CAL27 cells was measured with an Annexin V-fluorescein isothiocyanate (FITC) Apoptosis Detection Kit (C1062, Beyotime, Shanghai, China) following the kit protocol. Briefly, transfected cells were detached with ethylenediaminetetraacetic acid (EDTA)-free trypsin, washed twice with phosphate-buffered saline (PBS) and resuspended in 195 μL 1 x binding buffer at 1 x 10^5^ cells/mL. Cells were stained in the dark for 20 min at room temperature with 5 μL annexin V-FITC and 10 μL propidium iodide (PI). Analysis was performed within 1 hour post-staining using a BD FACSCanto II flow cytometer (BD Biosciences, San Jose, CA, USA). FlowJo software (Tree Star, Ashland, OR, USA) was used for data acquisition review and apoptosis quantification. The total apoptosis rate was estimated as the sum of the percentages of early apoptotic cells (Annexin V-positive/PI-negative) and late apoptotic cells (Annexin V-positive/PI-positive).

### Statistical analysis

The statistical analysis in the bioinformatics section was described in the corresponding methods section. Unless otherwise specified, the default statistical parameters from the relevant R packages were utilized.

Experimental data were analyzed and visualized in R v4.5.1. Continuous-variable normality was examined with the Shapiro-Wilk test. Variance homogeneity was evaluated with Levene’s test. Normally distributed datasets were compared with independent-samples t-tests. Specifically, if the Levene test showed homogeneity of variance (P > 0.05), Student’s t-tests were used. If the variances were unequal (P < 0.05), Welch-corrected t-tests were used. For abnormally distributed continuous data, Mann-Whitney U tests were used.

Furthermore, the chi-square test (or, if necessary, Fisher’s exact test) was used to compare categorical data between two groups.

Individual figure panels were generated or visualized in R, and final multi-panel figure layouts were assembled with EasyFigAssembler, a web-based figure assembly tool for publication-ready figures (32).

## Results

### Differentially expressed gene map and biological function annotation in head and neck squamous cell carcinoma

**Figure 2:**
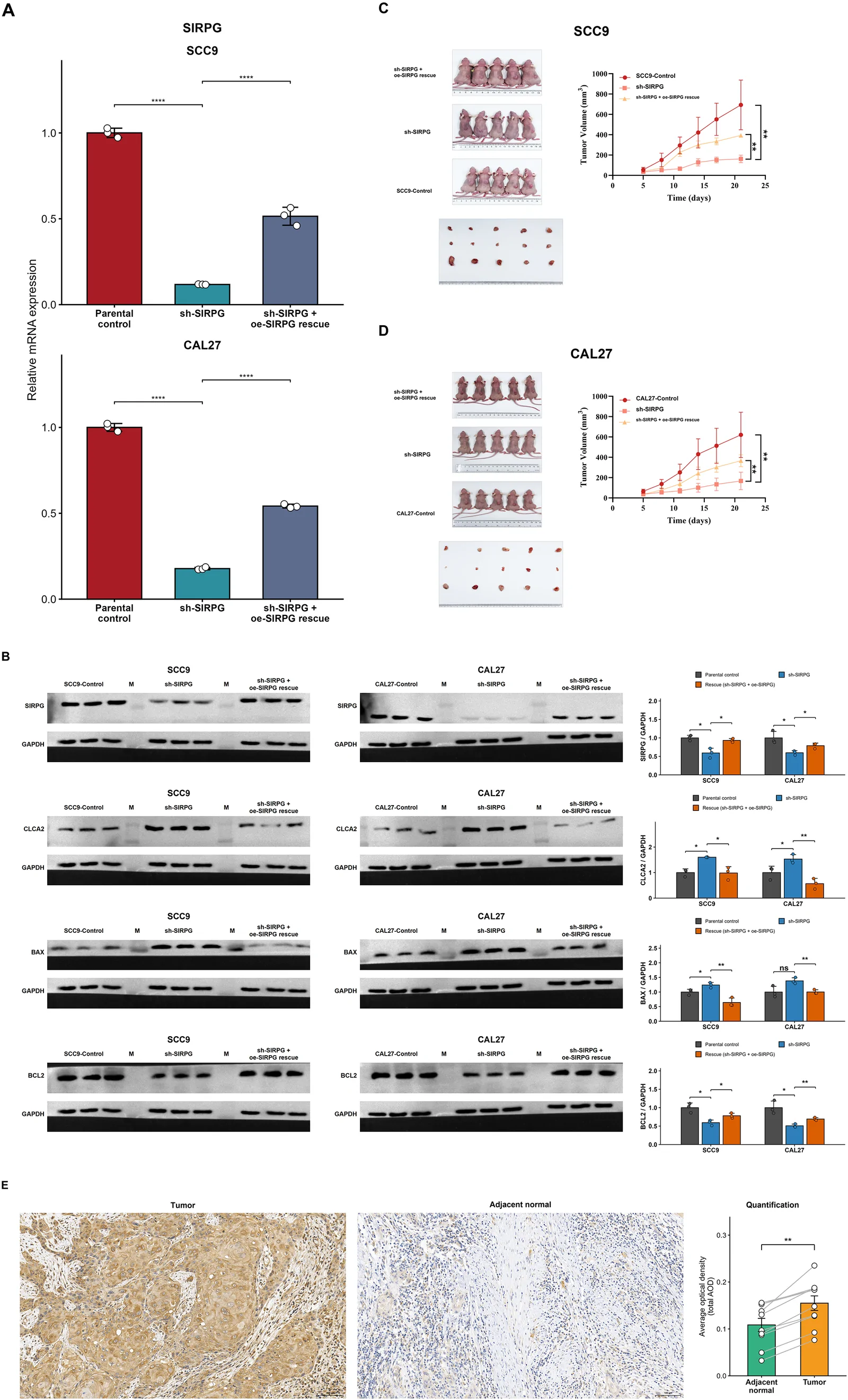
Transcriptomic landscape and functional enrichment analysis of differentially expressed genes in HNSCC. **(A)** Volcano plot illustrating differentially expressed genes (DEGs) between tumor and normal tissues. Red dots indicate significantly upregulated genes, blue dots indicate downregulated genes, and grey dots represent non-significant genes. Top significant genes are labeled. The x-axis represents the log2 fold change, and the y-axis represents the -log10 adjusted P-value. **(B, D)** Gene Ontology **(GO)** enrichment analysis of DEGs visualized as a bar plot **(B)** and a bubble plot **(D)**. Terms cover Biological Process (BP), Cellular Component (CC), and Molecular Function (MF). Bubble size corresponds to gene count, and color intensity indicates significance (p.adjust). **(C, E)** KEGG pathway enrichment analysis of DEGs shown as a bar plot **(C)** and a bubble plot **(E)**. **(F)** GSEA plot showing GO terms significantly downregulated in HNSCC, highlighting muscle-related functions. **(G)** GSEA plot showing KEGG pathways significantly enriched in HNSCC (e.g., Cell cycle) and downregulated pathways.

To characterize transcriptomic alterations in HNSCC, we compared TCGA-HNSC tumor samples with paired normal tissues. DESeq2 identified 7,574 differentially expressed genes (DEGs), including 3,462 upregulated genes and 4,112 downregulated genes. The volcano plot highlighted CA9 (log2FC = 5.85, adjusted P = 3.8 × 10^–42^) and MMP13 (log2FC = 5.75, adjusted P = 3.0 × 10^–25^) among the most significantly altered genes, consistent with hypoxia-related and extracellular-matrix-remodeling programs in tumor tissues (**Figure 2A**) (33).

GO analysis suggested that upregulated genes were significantly enriched in extracellular matrix tissue (adjusted P = 3.1 × 10^–24^) and nuclear division during biological processes. In terms of molecular functions and cellular components, they were primarily involved in extracellular matrix structural components and collagen-containing extracellular matrix (**Figures 2B, D**). KEGG analysis showed that DEGs were closely related to human papillomavirus (HPV) infection, cell cycle, and cytokine-cytokine receptor interaction pathways (**Figures 2C, E**). Next, GSEA-GO analysis revealed that muscle contraction-related functions were significantly downregulated in tumor samples, including myofibril, with a normalized enrichment score (NES) of -2.50, and sarcomeres (NES = -2.51). Hub-gene enrichment analysis suggested that the loss of expression of genes such as *KCNA5*, *CFL2*, and *KCTD6* was a key contributor to this downregulation (**Figure 2F**). Meanwhile, GSEA-KEGG analysis confirmed the significant activation of pro-cancer pathways. *PKMYT1* and *COL1A1* exhibited high enrichment in the cell cycle (NES = 2.51) and HPV infection (NES = 2.01) pathways. Conversely, the cytochrome P450 metabolism of exogenous substances pathway (NES = -2.22) was significantly suppressed. The low expression of *GSTT1* reflected the metabolic reprogramming characteristics of tumor cells (**Figure 2G**).

### Analysis of variations of expression profile of immune checkpoint molecules in HNSCC and their prognostic value

**Figure 3:**
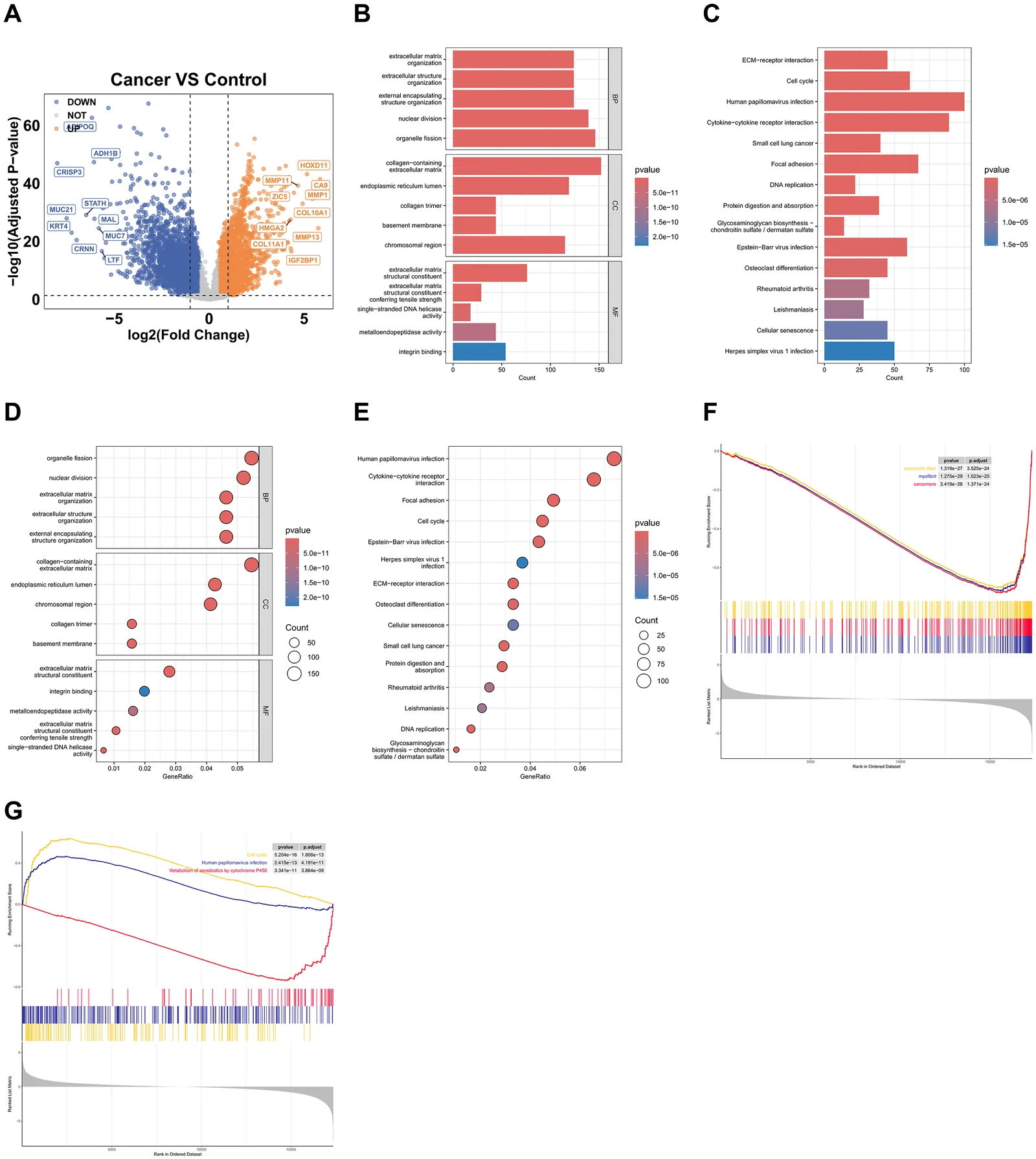
Expression patterns and prognostic significance of immune checkpoint genes in HNSCC. **(A–D)** Violin plots displaying the expression levels of 20 key immune checkpoints and ligands in HNSCC tumor tissues (blue) compared to normal tissues (red). The thick line inside the box represents the median value. Statistical significance was determined using the Wilcoxon rank-sum test (*P < 0.05, **P < 0.01, ***P < 0.001). **(E)** Forest plot summarizing the results of multivariate Cox proportional hazards regression analysis. The plot shows the Hazard Ratio (HR) and 95% Confidence Interval (CI) for each immune checkpoint. An HR > 1 indicates a risk factor for poor prognosis, while an HR < 1 indicates a protective factor. P-values are listed on the right.

We systematically evaluated the expression differences of 20 key immune checkpoint molecules between tumor and normal tissues. The results showed that most classic T-cell inhibitory receptors and their ligands were significantly downregulated in HNSCC tumor tissues. Specifically, the expression levels of 18 genes, encompassing *CTLA4, PDCD1* (PD-1)*, CD274* (PD-L1)*, TIGIT, LAG3*, and *HAVCR2* (TIM-3), were markedly lower in tumor samples relative to normal controls (P < 0.05) (**Figures 3A–C**). Conversely, *LGALS3* and *HMGB1* were significantly highly expressed in tumor tissues (**Figure 3D**). This expression pattern, characterized by low expression of classic T-cell exhaustion markers and high expression of selected immunomodulatory factors such as galectins, suggests that HNSCC may involve immunosuppressive mechanisms beyond the traditional PD-1/CTLA-4 pathway. The molecules with significant differential expression were incorporated into a multivariable Cox proportional hazards regression model for survival analysis. Forest plot indicated that these molecules had a significant and specific impact on patient prognosis (**Figure 3E**). Among them, *CD86* (hazard ratio (HR) = 1.051, 95% confidence interval (CI): 1.026-1.076, P < 0.001), *TIGIT* (HR = 1.126, P = 0.033), and *HMGB1* (HR = 1.005, P = 0.033) were identified as independent risk determinants for HNSCC. Their high expression levels were significantly associated with poor prognosis. Conversely, *CTLA4* (HR = 0.863, P < 0.001), *BTLA* (HR = 0.503, P = 0.005), and *LGALS9* (HR = 0.983, P = 0.020) exhibited significant protective effects. These results implied that maintaining a certain level of expression of these molecules may help maintain anti-tumor immune homeostasis or reflect better immune infiltration.

### Molecular typing and immune landscape analysis of HNSCC based on features of immune checkpoints

Based on an immune checkpoint-related gene set constructed based on Genecards (**Supplementary Table 2**), unsupervised consensus clustering analysis was performed on 520 tumor samples. Based on the heatmap and tracking map, the samples were clustered into three molecular subtypes: Cluster 1 (n = 113), Cluster 2 (n = 233), and Cluster 3 (n = 174) (**Figures 5A–C**; **Supplementary Table 3**).

**Figure 4:**
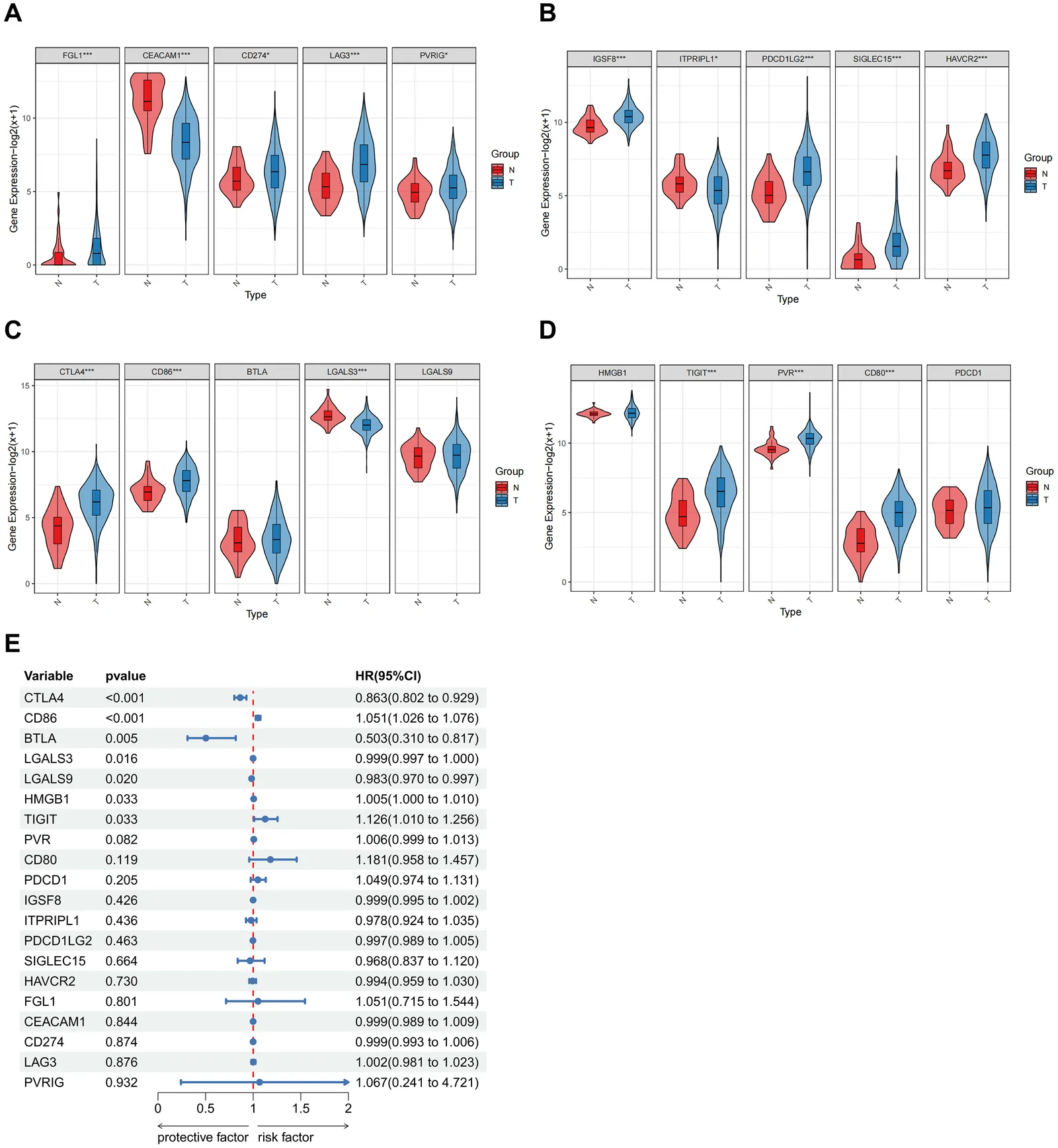
Consensus clustering and immune landscape characterization based on immune checkpoint-related signatures in HNSCC. **(A)** Legend for the consensus matrix, indicating co-clustering frequency. **(B)** Consensus clustering matrix heatmap at k = 3, separating samples into three clusters. **(C)** Tracking plot illustrating sample assignment changes across different k values. **(D)** UMAP visualization of the three identified clusters. Cluster 1 and Cluster 3 show distinct separation, while Cluster 2 exhibits an intermediate distribution. **(E)** Kaplan-Meier survival curves comparing OS between Cluster 1 and Cluster 3. Statistical significance was determined by the Log-rank test (P = 0.00038), showing a favorable prognosis for Cluster 1. **(F)** Comparison of immune cell infiltration levels between Cluster 1 and Cluster 3 using the ImmuCellAI platform. Results indicate significant enrichment of cytotoxic effector cells in Cluster 1, whereas Cluster 3 is characterized by immunosuppressive cells.

The UMAP dimensionality reduction algorithm was leveraged to visually represent the distribution relationships between clusters. The results indicated that Cluster 1 and Cluster 3 exhibited significant spatial separation, suggesting their distinct transcriptomic characteristics. Cluster 2, however, displayed a diffuse distribution intermediate between the two clusters, and was partially overlapped with the other two clusters. This pattern was consistent with an intermediate transitional state with less pronounced immune characteristics (**Figure 4D**). Given that this study intended to elucidate the key immune drivers in determining prognostic differences, we deeply analyzed the most distinctive Cluster 1 and Cluster 3 to capture significant phenotypic differences. Survival analysis showed a marked difference in clinical prognosis between the two clusters. Patients in Cluster 1 had markedly better OS than those in Cluster 3 (Log-rank P = 0.00038), suggesting that Cluster 1 was associated with a favorable prognosis (**Figure 4E**). We then comprehensively assessed the infiltration abundance of immune cells in both clusters using the ImmuCellAI online analysis platform. The analysis revealed markedly different immune landscapes in the two clusters. Cluster 1 exhibited significant immune activation, enriched with a large number of effector cells with anti-tumor activity, including CD8+ T cells, natural killer cells, mucosa-associated invariant T cells, and neutrophils. In contrast, Cluster 3 showed a marked immunosuppression or exhaustion state, significantly enriched with regulatory T cells and exhausted T cells (**Figure 4F**). This “hot” immune microenvironment dominated by lymphocytes was highly consistent with the favorable prognosis of Cluster 1, while the enrichment of suppressor cells in Cluster 3 reasonably explained its poor prognosis.

### Reconstructed 296-pipeline survival modelling identified SIRPG as a robust cross-cohort hub gene

**Figure 5:**
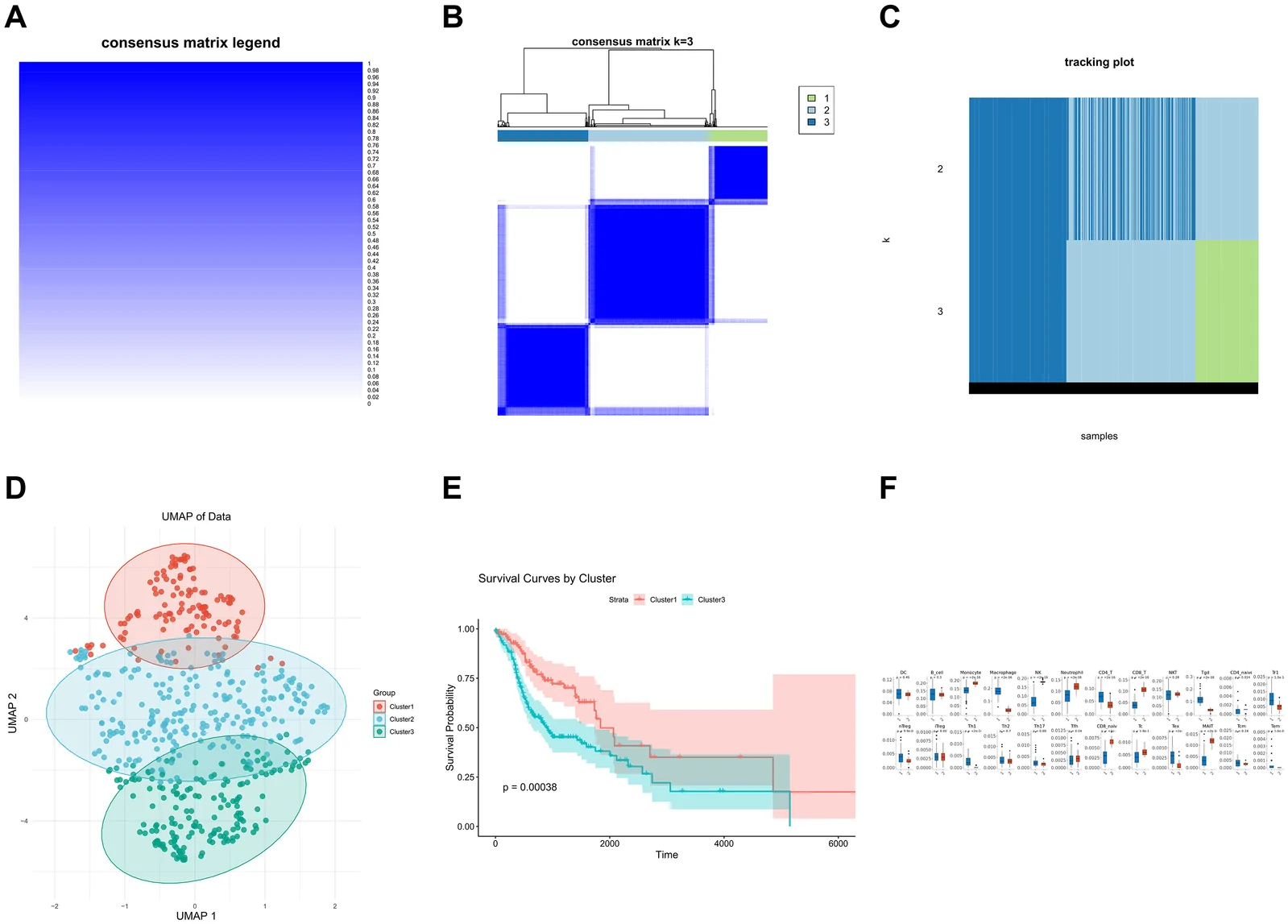
Immune-subtype DEG screening and 296-pipeline machine-learning prioritization of SIRPG. **(A)** Volcano plot showing DEGs between Cluster 1 and Cluster 3. Red dots indicate genes significantly upregulated in Cluster 1, blue dots indicate downregulated genes and grey dots indicate non-significant genes. **(B)** Hierarchical clustering heat map showing that the identified DEGs separated Cluster 1 from Cluster 3. **(C)** Heatmap showing the rank of core hub genes across five validation-cohort-specific analyses, including GSE10300, GSE27020, GSE31056, GSE41613 and GSE85446. Rows represent candidate hub genes and columns represent validation cohorts. **(D–H)** Lollipop plots showing the top ten selected genes in each validation-cohort-specific analysis. Dot size indicates screening frequency from the core-feature ranking module. SIRPG appeared among the top ten selected genes in all five validation analyses. **(I)** Meta-analysis of SIRPG expression in the five validation cohorts, showing the pooled HR and 95% CI.

To move from immune-subtype differences to candidate biomarkers, we compared Cluster 1 and Cluster 3 at the transcriptomic level. This comparison identified 1,029 DEGs, including 713 genes upregulated in Cluster 1 and 316 genes downregulated in Cluster 1 (**Figure 5A**). Hierarchical clustering showed that these DEGs separated the two immune subtypes, supporting their relevance to the survival and immune-infiltration differences described above (**Figure 5B**). The DEGs with P < 0.001 were then evaluated by univariable Cox regression to derive a prognosis-relevant candidate pool for downstream machine-learning screening (**Supplementary Figure 1**). We next reconstructed the previously reported 296-pipeline survival modelling framework and applied it to TCGA-HNSC together with five validation-cohort-specific candidate gene sets. The final source tables for all five **Supplementary Figure 2** panels contained exactly 296 distinct pipelines, with no missing or additional models relative to the reconstructed reference list. Across validation cohorts, the optimal pipeline differed, indicating cohort-dependent model performance (**Supplementary Figure 2**). SIRPG appeared among the top ten selected genes in all five validation-cohort-specific analyses, ranking 10th in GSE10300, 2nd in GSE27020, 1st in GSE31056, 10th in GSE41613 and 6th in GSE85446 (**Figures 5C–H**). Meta-analysis of SIRPG expression across the five validation cohorts showed an overall risk association (pooled HR = 1.514, 95% CI: 1.088-2.105, P = 0.014), supporting its context-dependent prognostic relevance and selection for subsequent expression, immune-correlation, single-cell and experimental validation (**Figure 5I**).

### Expression profile, prognostic value, and immune correlation analysis of *SIRPG* in pan-cancer and HNSCC

**Figure 6:**
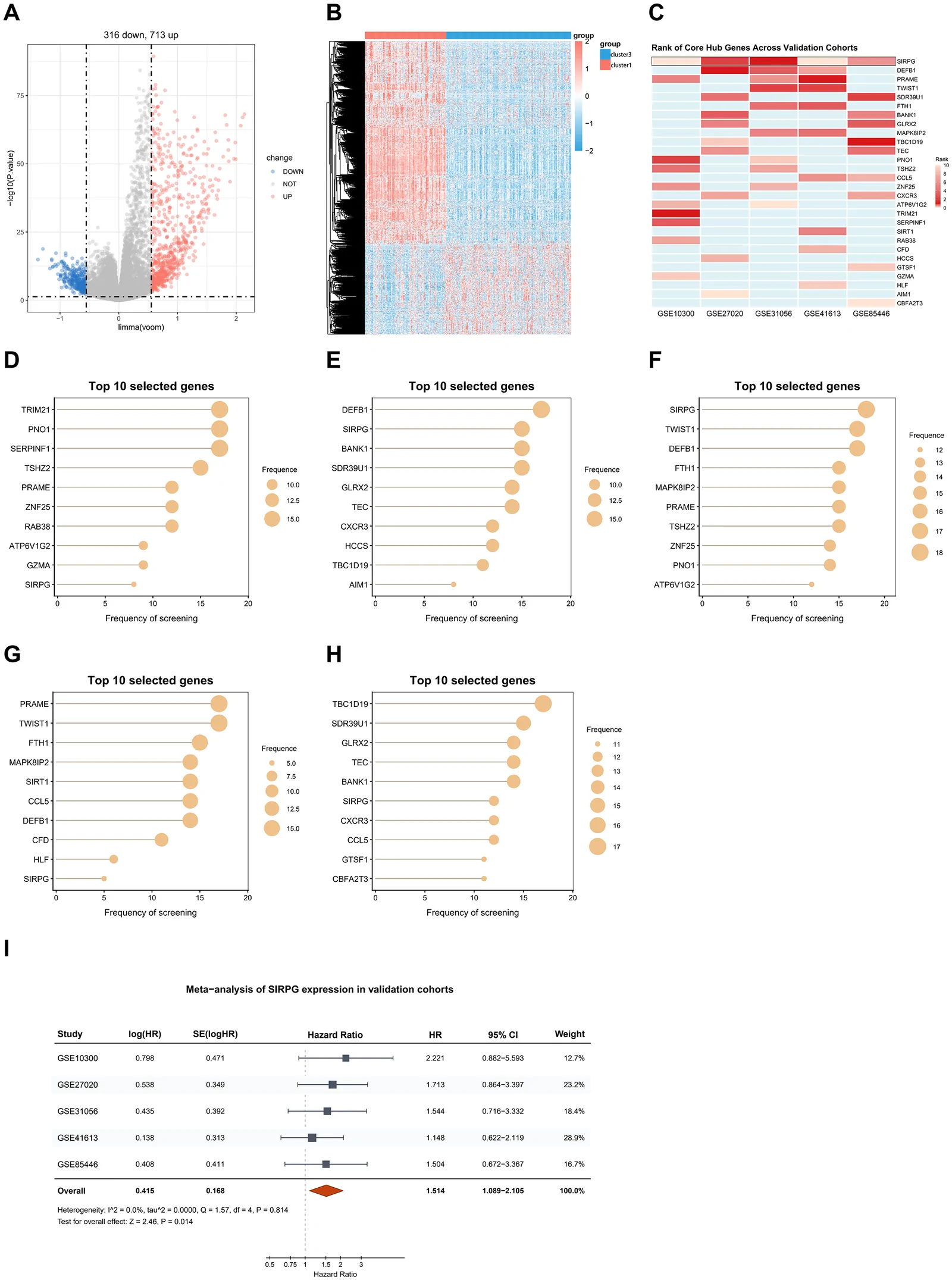
Multi-dimensional validation of SIRPG expression patterns, immune checkpoint correlations and prognostic significance. **(A)** Pan-cancer SIRPG expression landscape across tumor, normal and metastatic tissues. **(B)** Paired differential-expression analysis of SIRPG between tumor and adjacent-normal tissues in the TCGA-HNSC cohort. **(C)** GEPIA body-map visualization of median SIRPG expression across tumor and normal tissues. **(D–L)** Pearson correlation analyses between SIRPG and immune checkpoint or immune-regulatory genes, including BTLA, C10orf54/VSIR, CD274, CTLA4, HAVCR2, LAG3, PDCD1LG2, PDCD1 and TIGIT. **(M, N)** Kaplan-Meier survival curves for HNSCC patients stratified by SIRPG expression, showing overall survival **(M)** and disease-free survival **(N)**. *P < 0.05, **P < 0.01 and ***P < 0.001.

Given the central role of SIRPG screened by machine learning models, we further validated its expression patterns and clinical significance in pan-cancer and HNSCC. Paired sample analysis based on the TCGA-HNSC dataset showed that SIRPG expression was markedly elevated in tumor tissues relative to adjacent normal tissues (**Figure 6B**). Pan-cancer analysis further revealed heterogeneous SIRPG expression across tumor types, with notable upregulation in several solid tumors (**Figures 6A, C**). Pearson correlation analysis then showed that SIRPG was strongly associated with multiple immune checkpoint and immune-regulatory molecules, including BTLA, C10orf54/VSIR, CD274, CTLA4, HAVCR2, LAG3, PDCD1LG2, PDCD1 and TIGIT (**Figures 6D–L**). Survival analysis using GEPIA2 showed that high SIRPG expression was associated with better OS (Log-rank P = 0.0063, HR = 0.69) and DFS (Log-rank P = 0.0058, HR = 0.48), suggesting that SIRPG had a complex prognostic pattern in bulk HNSCC cohorts (**Figures 6M, N**).

The strong co-expression between SIRPG and immune checkpoint molecules suggested that SIRPG was tightly linked to T-cell activation, exhaustion and immune-regulatory programs in the HNSCC microenvironment. This pattern also raised an important interpretive issue: bulk SIRPG expression could reflect immune-cell infiltration as well as tumor-cell-intrinsic biology, requiring single-cell analysis to separate cell-source effects from malignant-cell states.

### CNV-supported single-cell analysis enabled tumor-cell-restricted assessment of *SIRPG*

**Figure 7:**
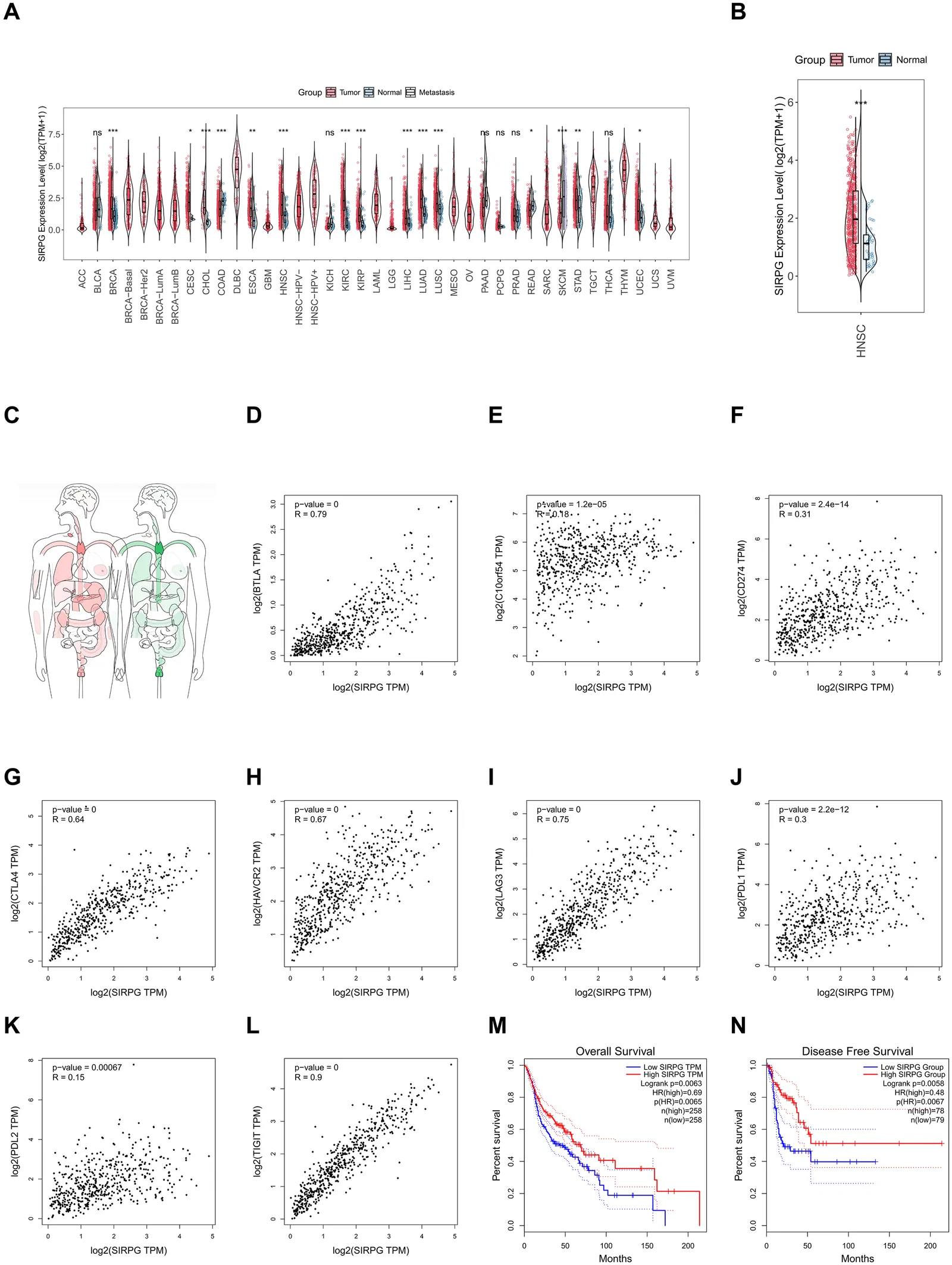
Multi-method malignant-cell validation and SIRPG-stratified HNSCC tumor-cell definition. **(A)** UMAP of the annotated GSE103322 single-cell atlas. **(B)** UMAP colored by sample identity to evaluate batch-related structure. **(C)** UMAP colored by cell-cycle phase. **(D)** Expression of SIRPG and inhibitory checkpoint genes BTLA, CTLA4, HAVCR2, LAG3 and TIGIT. **(E)** UMAP projection of transcriptome-inferred CNV scores. **(F)** CNV-score comparison across annotated cell types. **(G)** CopyKAT-based malignant-cell classification on the UMAP. **(H)** CopyKAT diploid/aneuploid proportions across annotated cell types. **(I)** scMalignantFinder malignancy-score projection on the UMAP. **(J)** scMalignantFinder malignancy-score comparison across annotated cell types. **(K–M)** Comparison of CNV score **(K)**, CopyKAT-defined aneuploid proportion **(L)** and scMalignantFinder malignancy probability **(M)** between SIRPG-high and SIRPG-low tumor cells. **** means P < 0.0001.

To resolve the bulk immune-correlation signal at cellular resolution, we first established a single-cell framework using the annotated HNSCC dataset GSE103322 from 18 patients. The UMAP embedding resolved tumor, immune, stromal, endothelial, and other cellular compartments, supporting the use of the published annotations for downstream analysis (**Figure 7A**). Sample labels and cell-cycle labels did not dominate the embedding, indicating that the major cell-state structure was not primarily driven by batch or cell-cycle effects (**Figures 7B, C**).

Within this annotated atlas, the UMAP distribution of SIRPG overlapped with checkpoint-related expression patterns, and correlation analysis further supported its association with BTLA, CTLA4, HAVCR2, LAG3 and TIGIT (**Figure 7D**; **Supplementary Figure 3A**). Before interpreting SIRPG-defined tumor-cell states, we first tested whether the annotated tumor-cell compartment was reliable. InferCNV-type CNV scoring showed higher CNV scores in tumor cells than in non-malignant cell types (**Figures 7E, F**). CopyKAT independently classified the annotated tumor-cell population as enriched for aneuploid malignant cells (**Figures 7G, H**). scMalignantFinder further assigned the highest malignancy scores to tumor cells, supporting the reliability of the malignant-cell annotation (**Figures 7I, J**). An extracted tumor-cell UMAP and concordance analysis across inferCNV, CopyKAT and scMalignantFinder provided additional support for the downstream malignant-cell framework (**Supplementary Figures 3B, C**).

After malignant-cell validation, we extracted tumor cells and analyzed SIRPG-associated heterogeneity within this malignant population. To improve comparability between SIRPG-defined groups, dropout correction was applied before tumor cells were divided by the median SIRPG expression value (**Supplementary Figures 3D, E**). SIRPG-high tumor cells showed higher CNV scores, a larger CopyKAT-defined aneuploid fraction and higher scMalignantFinder malignancy probabilities than SIRPG-low tumor cells, supporting downstream analyses focused on SIRPG-associated malignant-cell states (**Figures 7K–M**).

### *SIRPG*-high tumor cells exhibited immune-inhibitory and risk-associated programs

**Figure 8:**
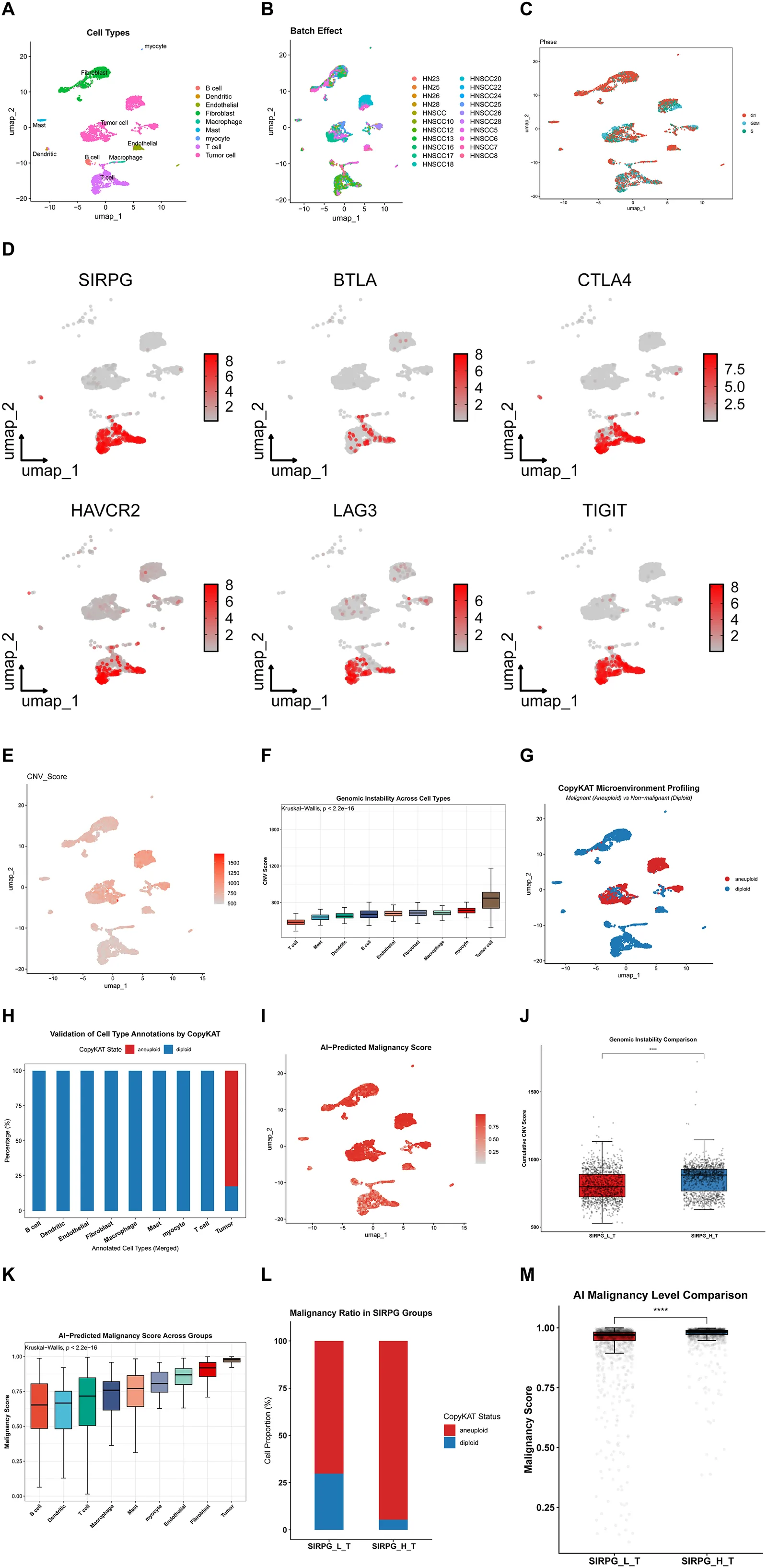
Immune-inhibitory, metabolically active, Scissor-positive and externally validated prognostic features of SIRPG-high tumor cells. **(A)** Differentially expressed genes between SIRPG-high and SIRPG-low tumor cells. **(B, C)** GO **(B)** and KEGG **(C)** enrichment analyses of SIRPG-associated genes. **(D)** Immune-inhibitory scores calculated using AddModuleScore, ssGSEA and UCell. **(E)** scMetabolism-based Reactome pathway scoring of 82 metabolic pathways in SIRPG-high and SIRPG-low tumor cells. **(F)** Scissor projection linking single-cell tumor states with bulk transcriptomic risk information. **(G)** Proportions of Scissor-positive, Scissor-negative and neutral cells across cell groups. **(H)** Intersection between Scissor-positive marker genes and survival-associated prognostic genes, defining a 17-gene Scissor-positive survival-related signature. **(I)** Kaplan-Meier survival validation of the 17-gene signature in GSE10300, GSE27020 and GSE31056. **(J)** Time-dependent ROC analyses comparing the 17-gene signature with clinical variables in GSE10300, GSE27020 and GSE31056. **** means P < 0.0001.

After defining SIRPG-stratified tumor cells, we investigated whether SIRPG-high cells represented a broader transcriptional state rather than a single-gene difference. Differential-expression analysis separated SIRPG-high from SIRPG-low tumor cells, indicating substantial transcriptional remodeling between the two groups (**Figure 8A**). GO enrichment linked SIRPG-associated genes to immune interaction, leukocyte adhesion, and lymphocyte differentiation processes (**Figure 8B**). KEGG enrichment extended this pattern to cytokine and T-cell receptor signaling pathways, placing SIRPG-high tumor cells in an immune-interactive transcriptional state (**Figure 8C**).

We next tested whether this immune-interactive state carried inhibitory and metabolic components. AddModuleScore, ssGSEA and UCell consistently showed higher inhibitory scores in SIRPG-high tumor cells (**Figure 8D**). In parallel, scMetabolism-based Reactome pathway scoring across 82 metabolic pathways showed that SIRPG-high tumor cells had higher activity in many, but not necessarily all, metabolic pathways, supporting a broadly metabolically active tumor-cell state (**Figure 8E**). The three immune-inhibitory scoring approaches were concordant in pairwise correlation analysis, strengthening the interpretation that this was a stable program-level signal (**Supplementary Figure 3F**).

To connect the single-cell state with clinical-risk information, Scissor was used to map tumor-cell states onto TCGA-HNSC bulk transcriptomic risk patterns (**Figure 8F**). SIRPG-high tumor cells contained a larger fraction of Scissor-positive, risk-associated cells than the SIRPG-low group, suggesting that the SIRPG-high state captured clinically adverse expression features (**Figure 8G**). To test whether this Scissor-positive state carried transferable prognostic information, we intersected Scissor-positive marker genes with survival-associated prognostic genes and obtained a 17-gene Scissor-positive survival-related signature (**Figure 8H**). Protein-protein interaction (PPI) analysis characterized the interaction structure of this signature, and four network-topology metrics, maximal clique centrality (MCC), maximum neighborhood component (MNC), Closeness and Degree, were further used to rank the top ten hub genes within the 17-gene network (**Supplementary Figures 4E–G**). ssGSEA-based scoring of this signature stratified OS in three independent validation cohorts, GSE10300, GSE27020 and GSE31056 (**Figure 8I**), whereas the remaining two validation cohorts were retained as supporting, non-confirmatory analyses (**Supplementary Figures 3G, H**). Time-dependent ROC analyses further compared the 17-gene signature with clinical variables in the three significant validation cohorts, supporting its external prognostic utility beyond a single Kaplan-Meier readout (**Figure 8J**).

### SIRPG perturbation linked pseudotime progression to CLCA2/P53-related regulatory programs

**Figure 9:**
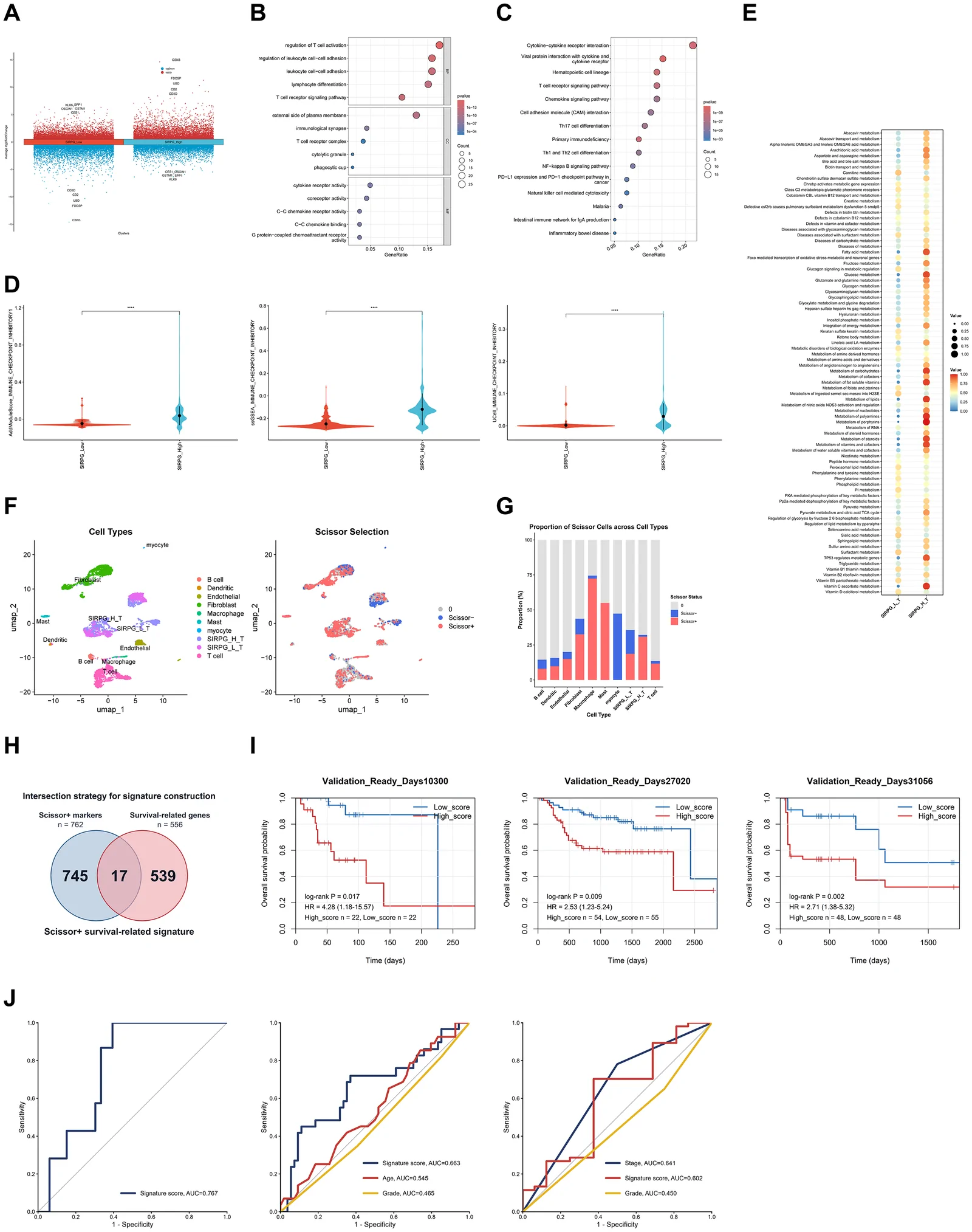
SIRPG-associated pseudotime progression, perturbation-sensitive CLCA2/P53-related programs and transcription-factor remodeling. **(A)** DDRTree pseudotime trajectory of tumor cells. **(B)** UMAP projection of tumor-cell pseudotime. **(C)** Immune-inhibitory score dynamics along the SIRPG-associated pseudotime trend. **(D)** Pseudotime dynamics of SIRPG, CD47, CLCA2, BAX and BCL2. **(E)** Virtual SIRPG knockout analysis showing network perturbation after simulated SIRPG depletion. **(F)** Three-way intersection of SIRPG-associated differentially expressed genes, virtual-knockout-affected genes and HALLMARK P53 pathway genes, nominating CLCA2. **(G)** Binarized SCENIC regulon activity in SIRPG-high and SIRPG-low tumor cells. **(H)** Regulators preferentially active in SIRPG-high tumor cells.

To further explore the mechanism of SIRPG in HNSCC tumor cells, we first evaluated differentiation potential as a boundary condition. CytoTRACE analyses did not support a SIRPG-high stemness-enriched pattern; instead, SIRPG-low tumor cells showed higher differentiation-potential scores, indicating that the tumor-promoting activity of SIRPG was unlikely to be explained by a simple stemness program (**Supplementary Figures 4A, B**). We then organized malignant cells along a DDRTree pseudotime trajectory and projected pseudotime onto the tumor-cell UMAP (**Figures 9A, B**), with ordering-gene selection shown in **Supplementary Figure 4C**. Along this trajectory, immune-inhibitory scores changed dynamically with the SIRPG-associated trend, and SIRPG showed coordinated temporal patterns with CD47, CLCA2 and apoptosis-related genes BAX and BCL2 (**Figures 9C, D**). To test whether SIRPG loss could perturb the tumor-cell gene-regulatory network, we performed virtual SIRPG knockout using scTenifoldKnk, which showed network perturbation after simulated SIRPG depletion (**Figure 9E**). Because the wet-lab apoptosis phenotype implicated the BAX/BCL2 balance, and because the HALLMARK P53 pathway is central to tumor progression, DNA-damage response and apoptosis regulation, we intersected SIRPG-associated DEGs, virtual-knockout-affected genes and HALLMARK P53 pathway genes. This three-way intersection prioritized CLCA2 as a candidate node linking SIRPG-associated tumor-cell states with P53-related apoptosis signaling (**Figure 9F**).

We next examined transcription-factor activity and found that multiple tumor-progression-related regulons were more active in SIRPG-high tumor cells than in SIRPG-low tumor cells (**Figure 9G**). Ranking analysis further highlighted STAT4, NKX3–1 and ZFP82 among the top SIRPG-high-associated regulators, supporting broader transcriptional regulatory remodeling in this state (**Figure 9H**). A detailed regulon heat map is provided as supporting evidence for this regulatory separation (**Supplementary Figure 4D**).

### SIRPG-high tumor cells exhibited prominent SIRPG-CD47/SIRP communication

**Figure 10:**
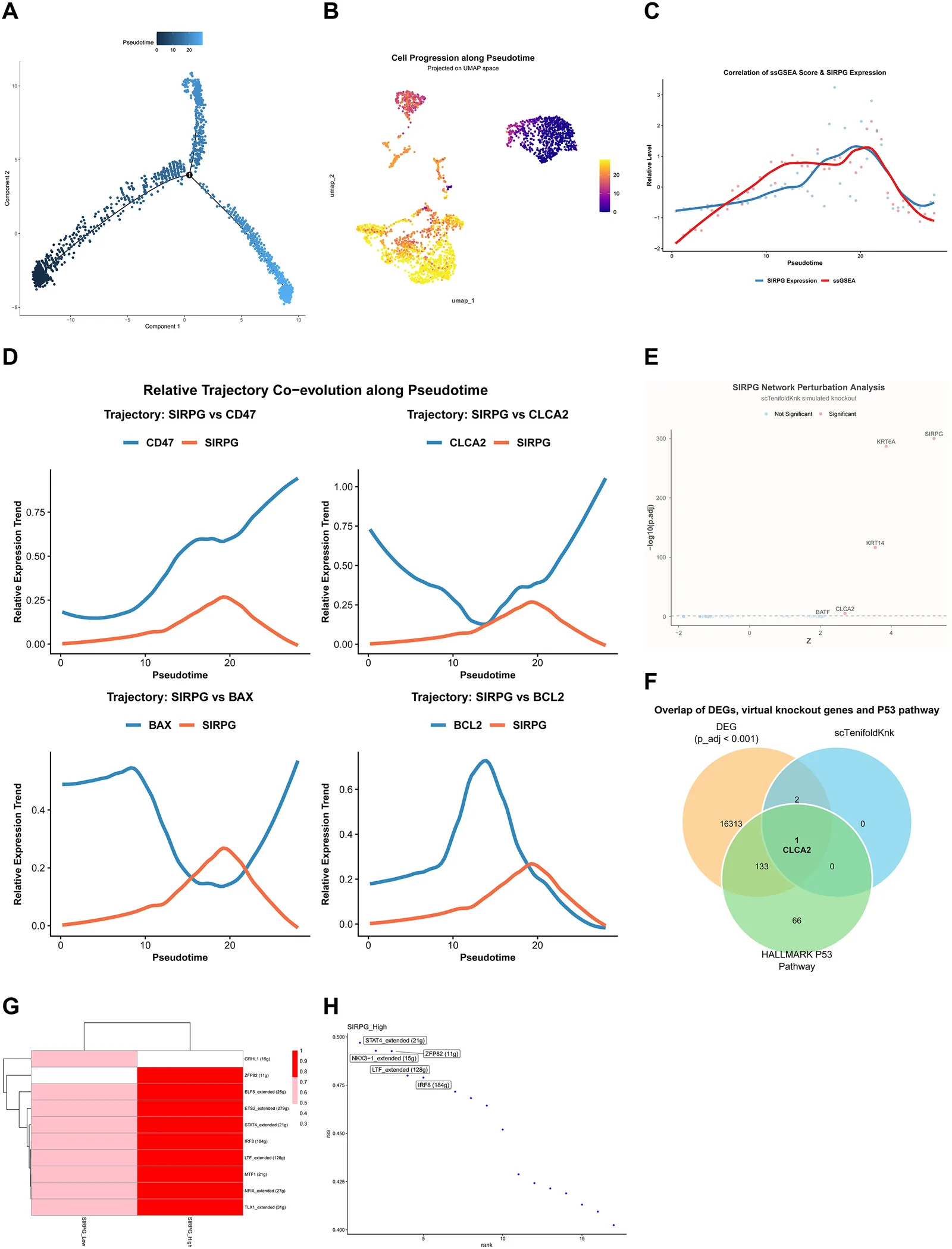
SIRPG-high tumor cells show prominent inferred SIRPG-CD47/SIRP communication with the tumor microenvironment. **(A)** Global inferred cell-cell communication numbers across annotated cell populations. **(B)** Comparison of selected immune-related ligand-receptor pathways, highlighting the SIRPG-CD47 interaction. **(C)** Outgoing and incoming interaction strengths within the SIRP pathway. **(D)** Cell-role heat map for the SIRP pathway. **(E)** SIRP-pathway communication network involving tumor and immune-cell populations. **(F)** Ligand-receptor contribution analysis showing the dominant contribution of SIRPG-CD47 to the inferred SIRP-pathway signal.

Because these intracellular changes may interact with the tumor microenvironment, we next performed cell-cell communication analysis as a separate communication-focused module. SIRPG-high tumor cells showed the greatest number of inferred interactions among the annotated cell populations (**Figure 10A**). Comparison of selected immune-related ligand-receptor pathways showed that SIRPG-CD47 communication had a higher inferred communication probability than several canonical pathways examined in parallel, including CD274-PDCD1, FASL-FAS and TNF-family interactions (**Figure 10B**). Focusing on the SIRP pathway, signaling-role analysis based on outgoing and incoming interaction strengths highlighted macrophages, T cells, SIRPG-high tumor cells and SIRPG-low tumor cells as major participating populations (**Figure 10C**). A pathway-role heat map further showed that macrophages and T cells acted as prominent receiver or influencer populations, whereas SIRPG-high tumor cells contributed more than SIRPG-low tumor cells to the SIRP signaling network (**Figure 10D**). The SIRP-pathway network and ligand-receptor contribution analysis further supported SIRPG-CD47 as the dominant communication pair in this inferred axis (**Figures 10E, F**). Collectively, these analyses suggest that SIRPG-high HNSCC tumor cells represent an immune-interactive state that combines inhibitory transcriptional programs, CLCA2/P53-related network changes and inferred SIRPG-CD47/SIRP communication.

### Spatial transcriptomics and protein-context analyses supported a SIRPG-associated tissue niche

**Figure 11:**
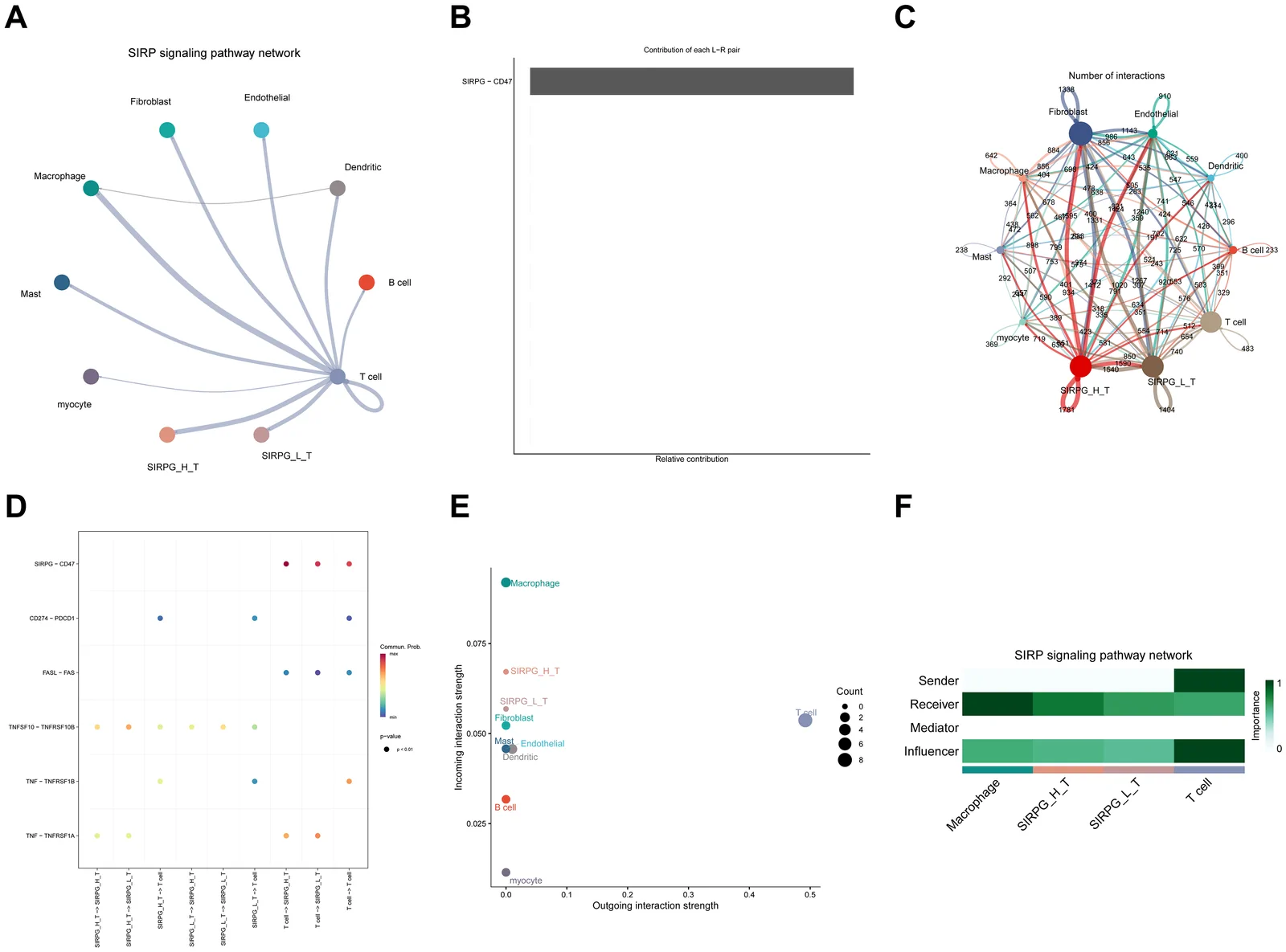
Spatial transcriptomics and CITE-seq-informed protein-context analyses support a SIRPG-associated immune-checkpoint niche in HNSCC tissues. **(A)** Spatial coherence of published tumor-region annotations assessed by real neighbor purity versus label-permuted controls. **(B)** Spatial distribution of SIRPG expression and immune-checkpoint signature activity in the representative section GSM6339637. **(C)** Maxspin-derived spatial partners of SIRPG, highlighting immune-checkpoint-related programs. **(D)** MISTy multiview analysis of local and neighboring programs explaining SIRPG expression. **(E)** COMMOT-inferred CD47-SIRPG communication trajectory across tumor regions. **(F)** scProTrans-inferred spatial overlap of CD47 and SIRPG protein-potential scores. **(G)** ProtTalksDB clinical proteomic context of SIRPG-associated checkpoint and apoptosis-related proteins. *P < 0.05 and ***P < 0.001.

To examine whether the inferred SIRPG-associated communication state had tissue-level spatial organization, we next analyzed the public OSCC Visium spatial transcriptomics cohort GSE208253, including 12 spatial transcriptomic sections and the representative section GSM6339637, using published tumor-region annotations (8). The published tumor core, transitory and leading-edge annotations showed significantly higher neighbor purity than permuted controls, indicating that these labels represented spatially coherent tissue domains rather than randomly scattered spots (**Figure 11A**). Independent BayesSpace clustering and marker visualization across sections further supported the stability of these regional annotations (**Supplementary Figures 5**. **6**).

After establishing region stability, we examined the SIRPG-associated niche. In the representative section GSM6339637, SIRPG expression was spatially aligned with immune-checkpoint signature activity (**Figure 11B**). Maxspin analysis identified the immune-checkpoint signature and multiple checkpoint molecules as prominent spatial partners of SIRPG, supporting non-random spatial coupling between SIRPG and checkpoint-related programs (**Figure 11C**) (25). MISTy multiview modelling further indicated that SIRPG variation was explained by both local intra-spot programs and neighboring spatial context, linking SIRPG to checkpoint activity, survival-associated programs and regional tumor architecture (**Figure 11D**). RCTD quality-control plots supported the reliability of the spatial deconvolution framework across sections (**Supplementary Figure 7**). Spots with a dominant RCTD cell-type weight greater than 0.6 were treated as high-confidence assignments, and their coherent spatial distribution paralleled the published histology-based tumor-region organization, further supporting the quality of the spatial transcriptomic dataset for downstream niche analysis.

We then examined ligand-receptor communication within this SIRPG-associated spatial niche as a separate transcriptome-based analysis. COMMOT suggested a CD47-SIRPG communication trajectory organized across tumor regions, with additional section-level visualizations supporting that this inferred flow was not limited to a single representative section (**Figure 11E**; **Supplementary Figure 8**). We next added a CITE-seq-informed protein-potential layer. scProTrans-inferred protein-potential maps showed spatial overlap between predicted CD47 and SIRPG scores, providing a transcriptome-derived approximation of protein-level co-localization rather than measured spatial proteomics (**Figure 11F**; **Supplementary Figure 9**). Finally, ProtTalksDB placed the SIRPG-associated spatial niche within a broader clinical proteomic context involving checkpoint and apoptosis-associated proteins, including CLCA2, BAX and BCL2-related programs (**Figure 11G**; **Supplementary Figure 10**). Taken together, the spatial analyses place SIRPG within an organized tissue niche characterized by checkpoint activity, inferred CD47-SIRPG communication and protein-context programs.

### SIRPG knockdown suppressed HNSCC cell survival and promoted apoptosis *in vitro*

**Figure 12:**
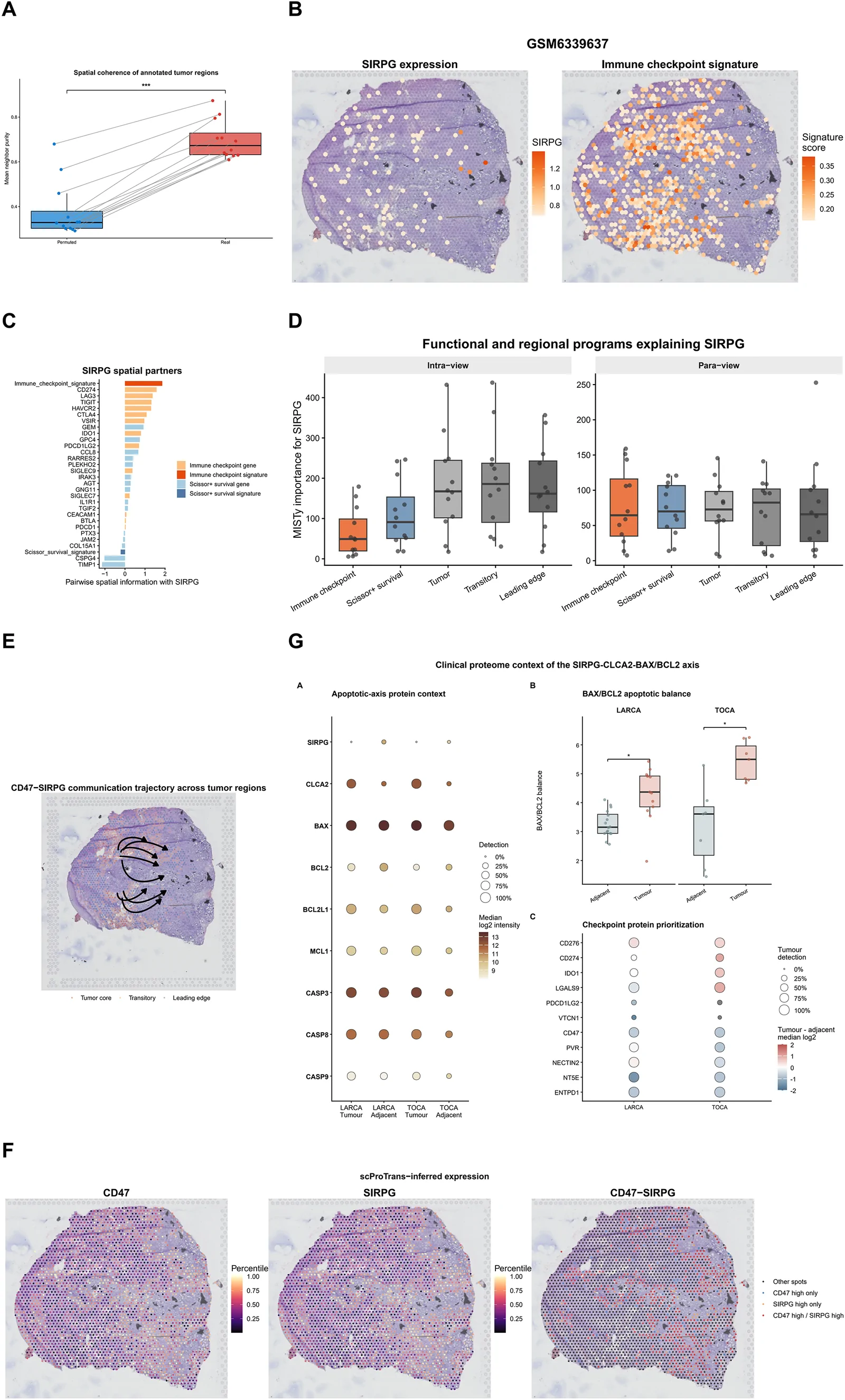
SIRPG knockdown suppresses HNSCC cell survival and promotes apoptosis *in vitro*. **(A)** Relative SIRPG mRNA expression in HOK normal cells and CAL27/SCC9 HNSCC cells. **(B–D)** Western blot analysis and quantification of SIRPG, BAX and BCL2 after transient SIRPG silencing. **(E, F)** CCK-8 cell-viability assays in CAL27 **(E)** and SCC9 **(F)** cells after SIRPG knockdown. **(G)** Flow-cytometry analysis of apoptosis after SIRPG knockdown. *P < 0.05, **P < 0.01 and ****P < 0.0001.

After these computational analyses, we tested the tumor-cell relevance of SIRPG *in vitro*. qRT-PCR showed that SIRPG expression was markedly higher in CAL27 and SCC9 HNSCC cells than in normal HOK cells (**Figure 12A**). Transient SIRPG silencing reduced SIRPG protein levels and shifted apoptosis-related markers, with BAX upregulation and BCL2 downregulation, supporting disruption of the BAX/BCL2 survival balance (**Figures 12B–D**). Functionally, SIRPG knockdown reduced cell viability in both CAL27 and SCC9 cells and increased apoptotic fractions by flow cytometry (**Figures 12E–G**). These functional assays indicate that SIRPG helps maintain HNSCC cell viability and limits apoptotic loss.

### SIRPG-associated therapeutic sensitivity, molecular docking and single-cell genetic-risk analysis

**Figure 13:**
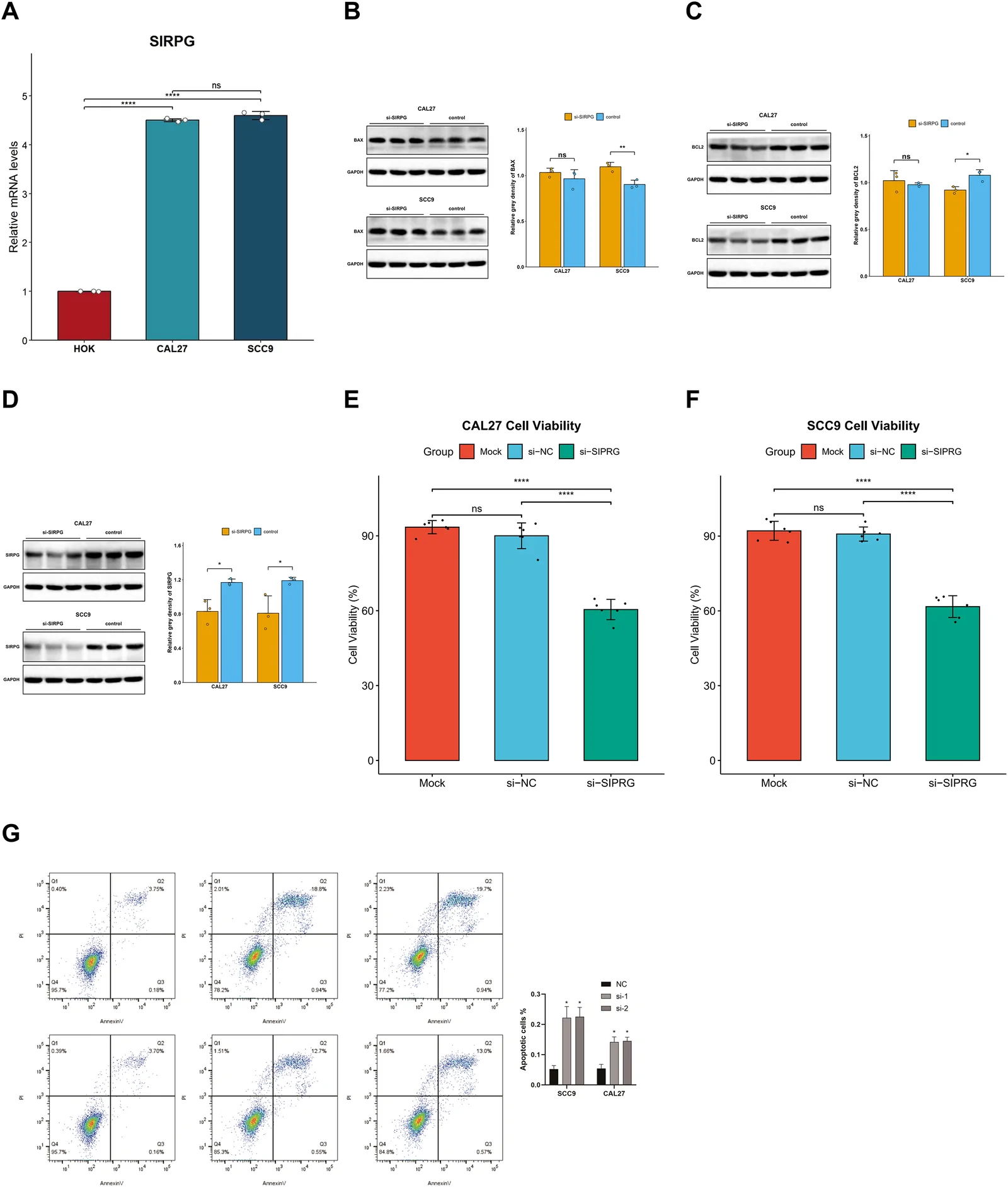
SIRPG-associated therapeutic sensitivity, molecular docking and single-cell genetic-risk analyses. **(A–D)** Box plots comparing predicted drug sensitivity between SIRPG-high and SIRPG-low HNSCC groups for 5-fluorouracil **(A)**, methotrexate **(B)**, paclitaxel **(C)** and bleomycin **(D)**. Lower predicted IC50 indicates greater predicted sensitivity. **(E)** Lollipop plot showing candidate SIRPG-associated therapeutic agents and pathway categories from pharmacogenomic drug-sensitivity analyses. **(F)** Molecular docking models between SIRPG and 5-fluorouracil, bleomycin, methotrexate and paclitaxel, with predicted binding affinities shown in kcal/mol. **(G)** Forest plot showing single-cell eQTL-based Mendelian randomization results for the association between genetically predicted SIRPG expression in immune-cell subsets and HNSCC risk. **(H)** Consolidated scPagwas genetic-risk score comparison between SIRPG-high and SIRPG-low tumor cells. Transcriptional risk score (TRS) values from 11 HNSCC-related GWAS datasets were Z-score-normalized within each GWAS and averaged per cell. **** means P < 0.0001.

We next examined whether SIRPG was linked to therapeutic-response patterns and genetically inferred cancer risk, with the complete sc-eQTL Mendelian randomization output provided in **Supplementary Table 4**. Among the evaluated small-molecule agents, SIRPG-high tumors showed lower predicted IC50 values for 5-fluorouracil (P = 0.0003), methotrexate (P = 0.015), paclitaxel (P = 0.00097) and bleomycin (P = 0.01), indicating greater predicted sensitivity to these agents (**Figures 13A–D**). GEPIA3 integration of pharmacogenomic information from CTRP, GDSC1 and GDSC2 further showed that SIRPG expression was associated with sensitivity to multiple candidate therapeutic agents, with enriched pathway categories including apoptosis regulation and phosphoinositide 3-kinase/mechanistic target of rapamycin (PI3K/mTOR) signaling (**Figure 13E**). Molecular docking added an exploratory structural layer: 5-fluorouracil, bleomycin, methotrexate and paclitaxel occupied distinct predicted binding regions on SIRPG, with paclitaxel showing the strongest predicted binding affinity among the tested agents (-7.867 kcal/mol), followed by methotrexate (-6.491 kcal/mol), 5-fluorouracil (-4.568 kcal/mol) and bleomycin (-4.427 kcal/mol) (**Figure 13F**; **Supplementary Table 5**).

We then introduced single-cell eQTL data from the OneK1K cohort and screened OpenGWAS outcomes related to HNSCC, oral cavity cancer and oropharyngeal cancer in a Mendelian randomization framework. The forest plot indicated that genetically predicted SIRPG expression was associated with cancer risk in specific immune-cell subpopulations. In CD4+ naive T cells, genetically predicted SIRPG expression was positively associated with head and neck cancer risk (ebi-a-GCST012239: odds ratio (OR) = 2.26, 95% CI: 1.02-5.02, P = 0.0446; ieu-b-95: OR = 2.26, 95% CI: 1.00-5.10, P = 0.0492). Higher SIRPG expression was also identified as a risk factor in natural killer receptor-positive cells and dendritic cells (OR > 1, P < 0.05) (**Figure 13G**; **Supplementary Table 4**). To complement this variant-instrument analysis, scPagwas projected 11 HNSCC-related GWAS datasets onto SIRPG-stratified tumor cells. After within-GWAS standardization and cell-level averaging, SIRPG-high tumor cells showed higher consolidated HNSCC genetic-risk scores than SIRPG-low tumor cells (**Figure 13H**). Individual GWAS projections showed some heterogeneity but supported the interpretation that HNSCC susceptibility signals were preferentially enriched in the SIRPG-high tumor-cell state (**Supplementary Figure 3I**).

### SIRPG re-expression rescued CLCA2-BAX/BCL2 signaling and xenograft growth after stable knockdown

**Figure 14:**
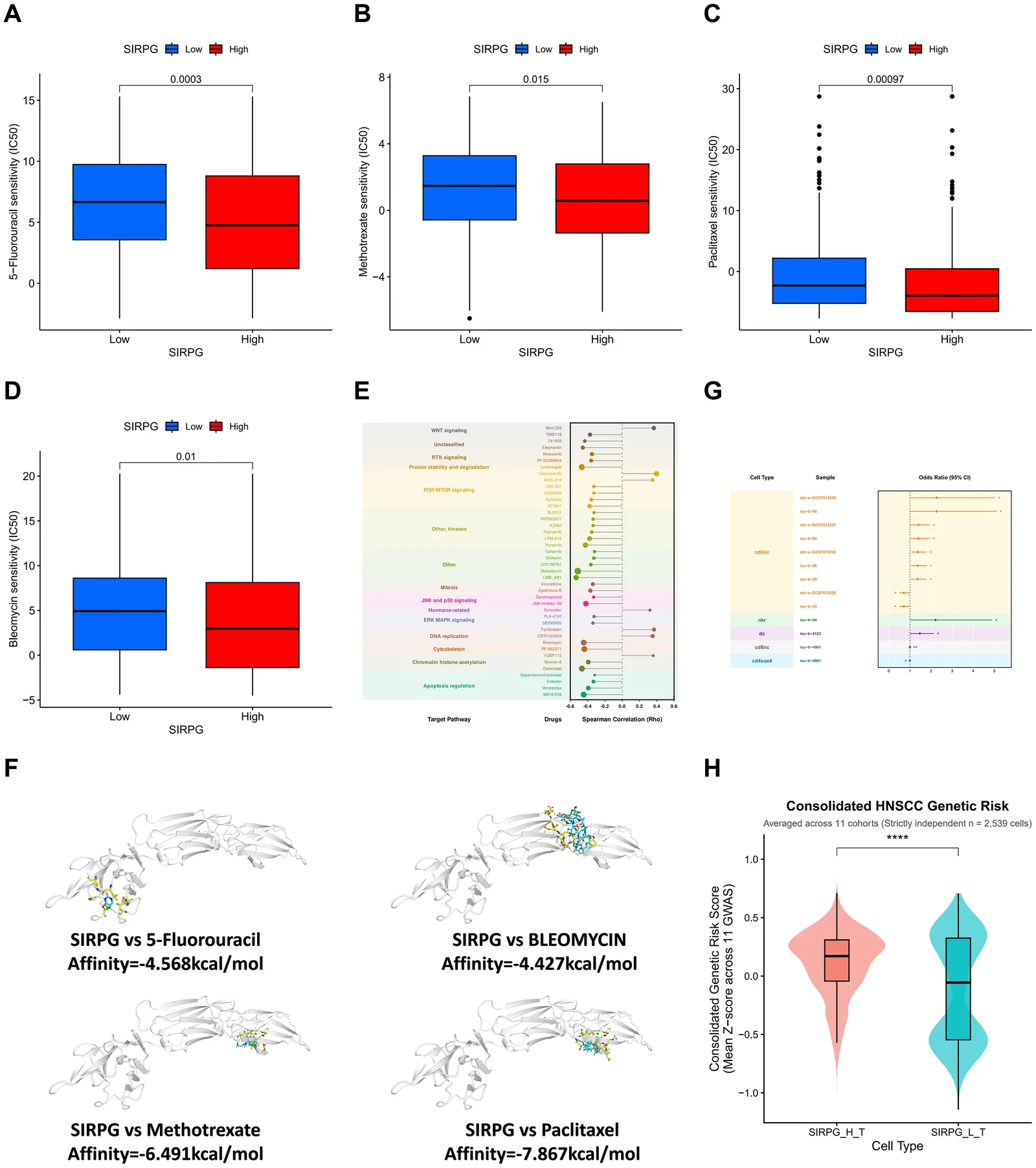
Rescue validation of the SIRPG-CLCA2-BAX/BCL2 axis and tissue-level SIRPG protein staining. **(A)** qRT-PCR analysis of SIRPG mRNA expression in parental SCC9-Control or CAL27-Control cells, stable sh-SIRPG cells and sh-SIRPG + oe-SIRPG rescue cells. The rescue group represents re-expression of an shRNA-resistant SIRPG sequence in the sh-SIRPG background. **(B)** Western blot analysis and quantification of SIRPG, CLCA2, BAX and BCL2 protein levels in SCC9 and CAL27 cells across the same three groups, showing reversal of the CLCA2/BAX/BCL2 response after SIRPG re-expression. Each lane represents one independently cultured biological replicate (n = 3 per group). For each cell line, samples were loaded on a single gel in the order parental control replicates 1–3, M, sh-SIRPG replicates 1–3, M and rescue replicates 1–3; M denotes a molecular-weight marker lane. All sample lanes were transferred together, and target proteins were normalized to GAPDH from matched lane positions derived from the same transfer. The membrane was not divided vertically by experimental group. Bars show mean ± SD, and points denote biological replicates. **(C, D)** Representative *in vivo* tumor images, excised tumor images and tumor-volume curves from SCC9 **(C)** and CAL27 **(D)** subcutaneous xenograft models. SIRPG knockdown suppressed tumor growth, whereas shRNA-resistant SIRPG re-expression partially restored tumor growth. n = 5 mice per group for each xenograft model. **(E)** Representative SIRPG immunohistochemistry images from tumor and adjacent normal tissue regions, together with section-level paired quantification. For each section, AOD values from three tumor fields and three adjacent-normal fields were averaged separately to generate one tumor value and one adjacent-normal value. n = 10 paired sections; two-sided Wilcoxon signed-rank test, *P < 0.05, **P < 0.01 and ****P < 0.0001.

Finally, we performed rescue experiments to test whether the molecular changes induced by SIRPG knockdown were specifically attributable to SIRPG loss. qRT-PCR confirmed that stable sh-SIRPG markedly reduced SIRPG mRNA expression in SCC9 and CAL27 cells, whereas shRNA-resistant SIRPG re-expression partially restored SIRPG expression in the sh-SIRPG + oe-SIRPG rescue group (**Figure 14A**). Western blotting further showed that SIRPG knockdown increased CLCA2 and BAX while reducing BCL2, and SIRPG re-expression reversed these changes across both HNSCC cell lines (**Figure 14B**). Each western-blot lane represented an independently cultured biological replicate, and all three groups for a given cell line were run on one gel and transferred together. We then extended this rescue design to subcutaneous xenograft models. In both SCC9 and CAL27 xenografts, sh-SIRPG tumors grew more slowly and remained smaller than their corresponding parental control tumors (SCC9-Control or CAL27-Control), whereas shRNA-resistant SIRPG re-expression partially restored tumor growth (**Figures 14C, D**). At the tissue level, representative immunohistochemistry (IHC) images showed stronger SIRPG staining in tumor regions than in adjacent normal regions, and section-level paired quantification confirmed higher average optical density in tumor regions across 10 paired sections (**Figure 14E**). Together, these rescue, xenograft and tissue-level IHC data support the specificity of the SIRPG-CLCA2-BAX/BCL2 axis and connect the CLCA2 node nominated by single-cell virtual perturbation analysis with both the apoptosis phenotype observed *in vitro* and the tumor-growth phenotype observed *in vivo*.

## Discussion

Although immunotherapy has improved the treatment landscape for HNSCC, durable responses remain limited and resistance continues to restrict clinical benefit (34). No single biomarker is likely to summarize the tumor immune microenvironment, and reliance on one prespecified algorithm can amplify overfitting and cohort-specific effects (35). We therefore combined immune-subtype analysis, a reconstructed 296-pipeline survival machine-learning framework, single-cell and spatial transcriptomic analyses, pharmacogenomic and genetic-risk analyses, and wet-lab validation to identify immune-related biomarkers with biological relevance in HNSCC.

A key clarification is that the present study did not aim to establish a fixed prognostic model for immediate clinical deployment. Instead, we reconstructed and adapted a previously reported large-scale selector-learner survival modelling strategy to prioritize repeatedly selected hub genes across independent validation settings. The 296-pipeline framework should therefore be interpreted as computational biomarker prioritization rather than as a newly invented algorithmic method. This reconstruction is still useful, because it reduces dependence on a single modelling choice and exposes genes that remain competitive across different selector-learner combinations and validation cohorts. Among the candidates, SIRPG showed repeated top-ten prioritization across all five validation-cohort-specific analyses and was selected for downstream immune, single-cell, spatial, pharmacological, genetic-risk and experimental analyses. In future work, this reconstructed framework could be standardized into a reusable R package for transparent selector-learner benchmarking, but such software development is beyond the scope of the current biological study.

SIRPG requires a cell-context-aware interpretation. SIRPG, also known as SIRP-gamma, is mainly expressed in immune-cell populations, especially T cells and natural killer cells, and participates in immune-cell interaction, adhesion and immune-response regulation (36). Previous studies have linked SIRPG to immune diseases and immune microenvironment remodeling in several tumor contexts, including lung squamous cell carcinoma and BRAF-mutant colorectal cancer. In the present study, TCGA clinical data suggested that high SIRPG expression was associated with better survival, whereas cellular experiments showed that SIRPG supported tumor-cell survival and suppressed apoptosis. This apparent paradox is plausible because bulk SIRPG expression may partly reflect immune-cell infiltration and an inflamed tumor microenvironment, while tumor-cell-intrinsic SIRPG may carry a distinct pro-survival function. Thus, SIRPG should not be interpreted as a uniformly protective or uniformly oncogenic marker without considering the cellular source and tissue context of its expression.

One methodological strength of the single-cell analysis is that we did not directly accept the annotated malignant compartment without additional validation. Accurate malignant-cell inference remains a major challenge (37) in tumor single-cell transcriptomic analysis, because different computational methods capture distinct biological signals and each method has its own bias. Given that the available GSE103322 data consisted of expression matrices rather than raw reads, DNA-level mutation calls, allele-specific read counts or fusion evidence, read-level methods such as Numbat, scAllele, Monopogen, STAR-Fusion and scFusion were not directly applicable. Likewise, gene-set or signature-list approaches such as UCell, GSVA and Ikarus can be useful for program scoring but may introduce subjectivity when used as primary malignant-cell callers. We therefore selected three complementary expression-matrix-compatible strategies: inferCNV-type CNV scoring, CopyKAT aneuploidy classification and scMalignantFinder malignant probability. Their concordant support for the annotated tumor-cell compartment increased confidence that downstream SIRPG-high versus SIRPG-low comparisons were performed within a reliable malignant-cell framework.

Single-cell analysis helped separate malignant-cell-associated SIRPG programs from signals attributable to immune-cell infiltration. SIRPG-high malignant cells carried immune-inhibitory transcriptional programs, increased activity in many Reactome metabolic pathways and a larger fraction of Scissor-positive risk-associated cells. However, CytoTRACE argued against interpreting SIRPG-high cells as a stemness-enriched population. Instead, the combined pseudotime, virtual perturbation and HALLMARK P53 intersection analyses support a later immune-interactive tumor-cell state linked to CLCA2/P53-related perturbation signals and the BAX/BCL2 apoptosis axis. The rescue experiments strengthened this interpretation by showing that shRNA-resistant SIRPG re-expression reversed the CLCA2-BAX/BCL2 protein response after stable SIRPG knockdown and partially restored xenograft tumor growth *in vivo*.

Spatial transcriptomics then placed this candidate axis in its tissue context. Because the original GSE208253 study provided tumor core, transitory-region and leading-edge annotations, we first tested whether these annotations formed coherent spatial domains in the sections used here. Neighbor-purity analysis and BayesSpace support reduced the risk that downstream spatial findings were driven by arbitrary region labels. We then used Maxspin to quantify spatial coupling between SIRPG and checkpoint-related features. From an information-theoretic perspective, this choice is relevant because spatial co-localization is a symmetric biological question. Classical Kullback-Leibler divergence is directional, meaning that the divergence from P to Q is generally not the same as the divergence from Q to P (38). This asymmetry is awkward for spatial co-localization analysis, because the inferred similarity between SIRPG and a checkpoint-related program should not depend on which feature is placed first. Kullback-Leibler divergence is also sensitive to zero-probability entries, which is a practical concern for spatial transcriptomic matrices that contain many zero values because of limited capture efficiency and platform sensitivity. Jensen-Shannon divergence addresses these issues by comparing each distribution with a shared mixture distribution. This construction is symmetric, better behaved for sparse distributions and has a metric form after square-root transformation (39). Therefore, the Jensen-Shannon-based information-theoretic structure of Maxspin provides a more interpretable way to quantify SIRPG-checkpoint spatial coupling in this study (25). Together with MISTy, COMMOT, scProTrans and ProtTalksDB, the spatial analyses support a SIRPG-associated immune-checkpoint-coupled niche, while retaining the boundary that these results are retrospective and inference-based.

From a clinical translation perspective, SIRPG expression could be evaluated using qRT-PCR, targeted RNA sequencing, NanoString-based panels or RNA *in situ* hybridization on pretreatment biopsy or formalin-fixed paraffin-embedded specimens. Such assays should not be interpreted in isolation. They should be integrated with conventional clinicopathological variables, HPV status, anatomical subsite, immune infiltration markers and treatment information. For example, SIRPG-high tumors with strong CD8+ T-cell or natural killer-cell infiltration may represent immune-inflamed tumors, whereas SIRPG-high tumors with weak cytotoxic infiltration may indicate a setting in which tumor-cell-associated SIRPG activity is more relevant. Accordingly, clinical translation should interpret SIRPG together with microenvironmental features rather than as an isolated marker. Therapeutically, the present findings justify further evaluation of SIRPG-centered intervention strategies but do not yet establish SIRPG as a ready clinical target. Potential strategies include SIRPG-neutralizing antibodies, blocking peptides, engineered protein binders or bispecific constructs that couple SIRPG blockade with a tumor-enriched antigen or immune-checkpoint target (40, 41). However, SIRPG is not tumor-specific and may be expressed in immune-cell populations, particularly T cells and natural killer cells. Systemic SIRPG blockade may therefore perturb normal immune-cell communication or produce heterogeneous effects depending on the dominant cellular source of SIRPG in each tumor. Any future therapeutic strategy should prioritize tumor- or microenvironment-selective delivery, conditional activation and biomarker-guided patient selection (42, 43).

This study also has important limitations. First, although the work includes a large amount of multi-omics validation, it remains centered on one target gene rather than a newly defined molecular subtype. Unsupervised frameworks such as non-negative matrix factorization or high-definition weighted gene co-expression network analysis could be used in future studies to build a more hierarchical subtype model. Second, although we incorporated CITE-seq-informed protein-potential inference, the multi-omics evidence is still incomplete. Assay for transposase-accessible chromatin using sequencing (ATAC-seq), chromatin immunoprecipitation sequencing (ChIP-seq), single-nucleus RNA-seq (snRNA-seq), spatial metabolomics and spatial proteomics were not available in the current work. Third, COMMOT and CellChat infer ligand-receptor communication from expression data and cannot prove that SIRPG-CD47 signaling directly changes T-cell or macrophage function. Fourth, although knockdown and rescue experiments support the specificity of the SIRPG-CLCA2-BAX/BCL2 axis, deeper molecular validation, including co-immunoprecipitation, mass spectrometry, point-mutant rescue, patient-derived xenografts and immune co-culture assays, will be needed to define direct binding partners and functional immune consequences. In all, this breadth strengthens confidence in SIRPG, but future work should add deeper mechanistic experiments and prospective treatment-response cohorts to define its translational boundary.

## Conclusion

This study integrated multi-omics data mining, a reconstructed 296-pipeline survival machine-learning framework, single-cell and spatial transcriptomic analyses and multi-layered experimental validation to identify SIRPG as a robust prognostic hub gene and candidate context-dependent regulator in HNSCC. Biologically, although SIRPG appears as a protective factor in some clinical bulk analyses, tumor-cell-focused evidence indicates that it can support proliferation and suppress apoptosis. Mechanistically, single-cell analyses connected SIRPG-high tumor cells to immune-inhibitory and metabolically active programs, CLCA2/P53-related perturbation signals and inferred SIRPG-CD47/SIRP communication, while spatial analyses placed this axis within an immune-checkpoint-coupled tissue niche. Stable shRNA rescue, xenograft assays and tissue-level immunohistochemistry further support the specificity and *in vivo* relevance of the SIRPG-CLCA2-BAX/BCL2 axis. These findings provide a framework for interpreting the context-dependent role of SIRPG in HNSCC and support further experimental evaluation of SIRPG-centered tumor-cell and microenvironmental mechanisms.

In summary, this study provides a multi-layered framework for prioritizing immune-related biomarkers in cancer research and characterizes SIRPG as a candidate therapeutic and mechanistic target that warrants deeper molecular and immunological validation in HNSCC.

## Data Availability

The publicly available datasets analyzed in this study are available from TCGA/UCSC Xena, NCBI Gene Expression Omnibus (GSE10300, GSE27020, GSE31056, GSE41613, GSE85446, GSE103322, GSE208253 and GSE213264), the IEU OpenGWAS database and the OneK1K cohort. Additional public pharmacogenomic, structural and proteomic resources are described in the Methods. Data generated in this study are included in the article and Supplementary Material. Further inquiries may be directed to the corresponding authors.

## Supplementary material

Supplementary Table 1 Primer, siRNA and lentiviral vector information used in this study.

Supplementary Table 2 Immune checkpoint-related immune signature.

Supplementary Table 3 Detailed grouping of cluster 1 and cluster 3.

Supplementary Table 4 Results of sc-eQTL Mendelian randomization analysis across the 11 retained GWAS datasets.

Supplementary Table 5 Molecular docking scores between SIRPG and selected chemotherapeutic agents.

Supplementary Figure 1 Univariable Cox screening of immune-subtype DEGs. Univariable Cox regression was performed for the 1,029 DEGs identified between Cluster 1 and Cluster 3. Genes significantly associated with OS were ranked according to prognostic significance, and high-confidence candidates were retained for downstream ensemble machine-learning analysis.

Supplementary Figure 2 Performance landscape of the reconstructed 296-pipeline survival machine-learning framework across validation cohorts. Each panel summarizes the performance of the reconstructed 296-pipeline survival modelling framework for one validation-cohort-specific candidate gene set. The pipeline grid followed the original **Figure 5A**-style structure, consisting of 21 stand-alone survival models and 275 two-stage selector-learner models generated from 11 feature selectors and 25 survival learners. Models were trained in the TCGA-HNSC cohort and evaluated in the corresponding GEO validation cohort. Rows represent individual pipelines, and columns show the validation cohort, the TCGA-HNSC training cohort and the mean C-index across the paired training-validation evaluation. Cell colors and overlaid values indicate the concordance index (C-index). The final source tables contained 296 distinct pipelines in each validation run, with no missing or additional models relative to the reconstructed original **Figure 5A** pipeline standard.

Supplementary Figure 3 Supporting analyses for SIRPG-associated malignant-cell stratification, Scissor validation and scPagwas genetic-risk projection. **(A)** Pairwise correlations between SIRPG and inhibitory checkpoint genes. **(B)** UMAP of extracted tumor cells used for downstream SIRPG-focused analysis. **(C)** Concordance analysis among inferCNV, CopyKAT and scMalignantFinder for malignant-cell identification. **(D, E)** Distribution of SIRPG-high and SIRPG-low tumor-cell groups before **(D)** and after **(E)** dropout correction. **(F)** Pairwise correlations among immune-inhibitory scores calculated by AddModuleScore, ssGSEA and UCell. **(G, H)** Kaplan-Meier survival validation of the 17-gene Scissor-positive survival-related signature in GSE41613 **(G)** and GSE85446 **(H)**. **(I)** Density distributions of normalized scPagwas TRS values across 11 individual HNSCC-related GWAS datasets, comparing SIRPG-high and SIRPG-low tumor cells.

Supplementary Figure 4 Supporting analyses for SIRPG-associated differentiation, pseudotime, regulatory programs and Scissor-positive signature PPI network. **(A)** CytoTRACE scores projected onto the tumor-cell UMAP. **(B)** CytoTRACE-score comparison between SIRPG-high and SIRPG-low tumor cells. **(C)** Ordering-gene selection for pseudotime analysis. **(D)** Detailed SCENIC regulon activity heat map across SIRPG-high and SIRPG-low tumor-cell states. **(E)** STRING database protein-protein interaction network of the 17-gene Scissor-positive survival-related signature. **(F)** Cytoscape visualization of the 17-gene PPI network. (G) Hub-gene ranking of the 17-gene signature using four network-topology algorithms, including MCC, MNC, Closeness and Degree.

Supplementary Figure 5 BayesSpace support for published spatial-region annotations. BayesSpace q=9 clusters were mapped to published tumor core, transitory-region and leading-edge annotations across 12 spatial transcriptomic sections.

Supplementary Figure 6 Marker expression and spatial annotation across 12 spatial transcriptomic sections. The combined DotPlot and spatial annotation view supports the region-level interpretation used for downstream SIRPG spatial-niche analysis.

Supplementary Figure 7 RCTD deconvolution quality assessment across spatial transcriptomic sections. RCTD used the single-cell reference to deconvolute Visium spots. Spots with a dominant cell-type weight greater than 0.6 were defined as high-confidence assignments; their distribution, together with second-largest weights and second-largest cell-type patterns, was used to assess concordance with the histology-based regional organization across sections.

Supplementary Figure 8 COMMOT-inferred CD47-SIRPG regional communication flow across 12 spatial transcriptomic sections. These section-level maps support that the inferred CD47-SIRPG communication pattern was not restricted to a single representative tissue section.

Supplementary Figure 9 scProTrans-inferred CD47 and SIRPG protein-potential maps across 12 spatial transcriptomic sections. These analyses provide CITE-seq-informed, transcriptome-derived protein-potential support for spatial CD47/SIRPG overlap and should not be interpreted as measured spatial proteomics.

Supplementary Figure 10 ProtTalksDB context for SIRPG abundance and detectability. The analysis provides external clinical proteome-context support for interpreting SIRPG-associated checkpoint and apoptosis-related programs.
