# Supplementary material for: Multi-omics identification and functional validation of signal regulatory protein gamma as a prognostic biomarker and immune regulator in head and neck squamous cell carcinoma
Source: Front Immunol. 2026 Aug 21;17:1835156. doi: 10.3389/fimmu.2026.1835156 (PMC13541685; doi:10.3389/fimmu.2026.1835156)
Supplement: Supplementary file 1 [file DataSheet1.pdf]

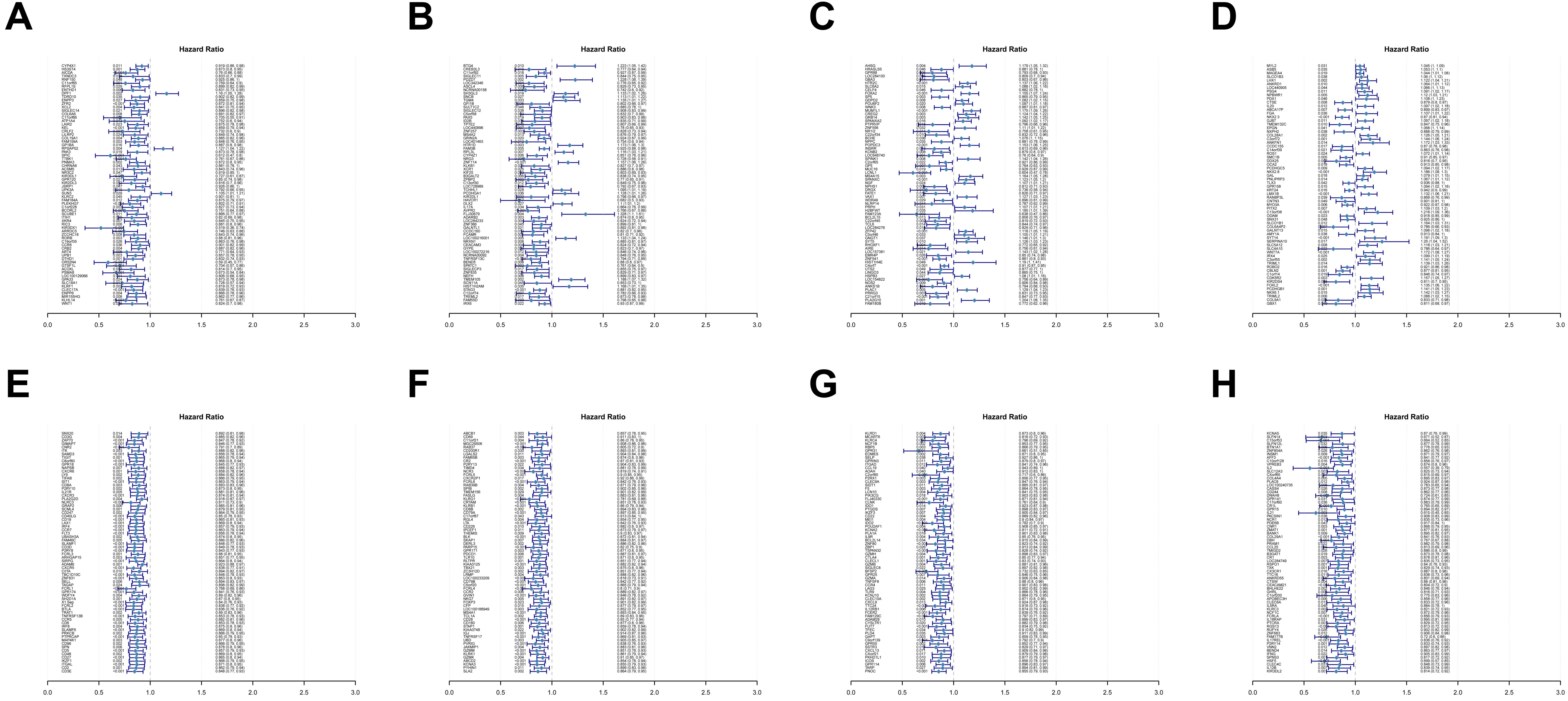
A
B
C
D
E
F
G
H
Hazard Ratio
0.0
0.5
1.0
1.5
2.0
2.5
3.0
