# Supplementary material for: Multi-omics identification and functional validation of signal regulatory protein gamma as a prognostic biomarker and immune regulator in head and neck squamous cell carcinoma
Source: Front Immunol. 2026 Aug 21;17:1835156. doi: 10.3389/fimmu.2026.1835156 (PMC13541685; doi:10.3389/fimmu.2026.1835156)

Supplementary context for SIRPG/Q9P1W8 in ProtTalks-DB

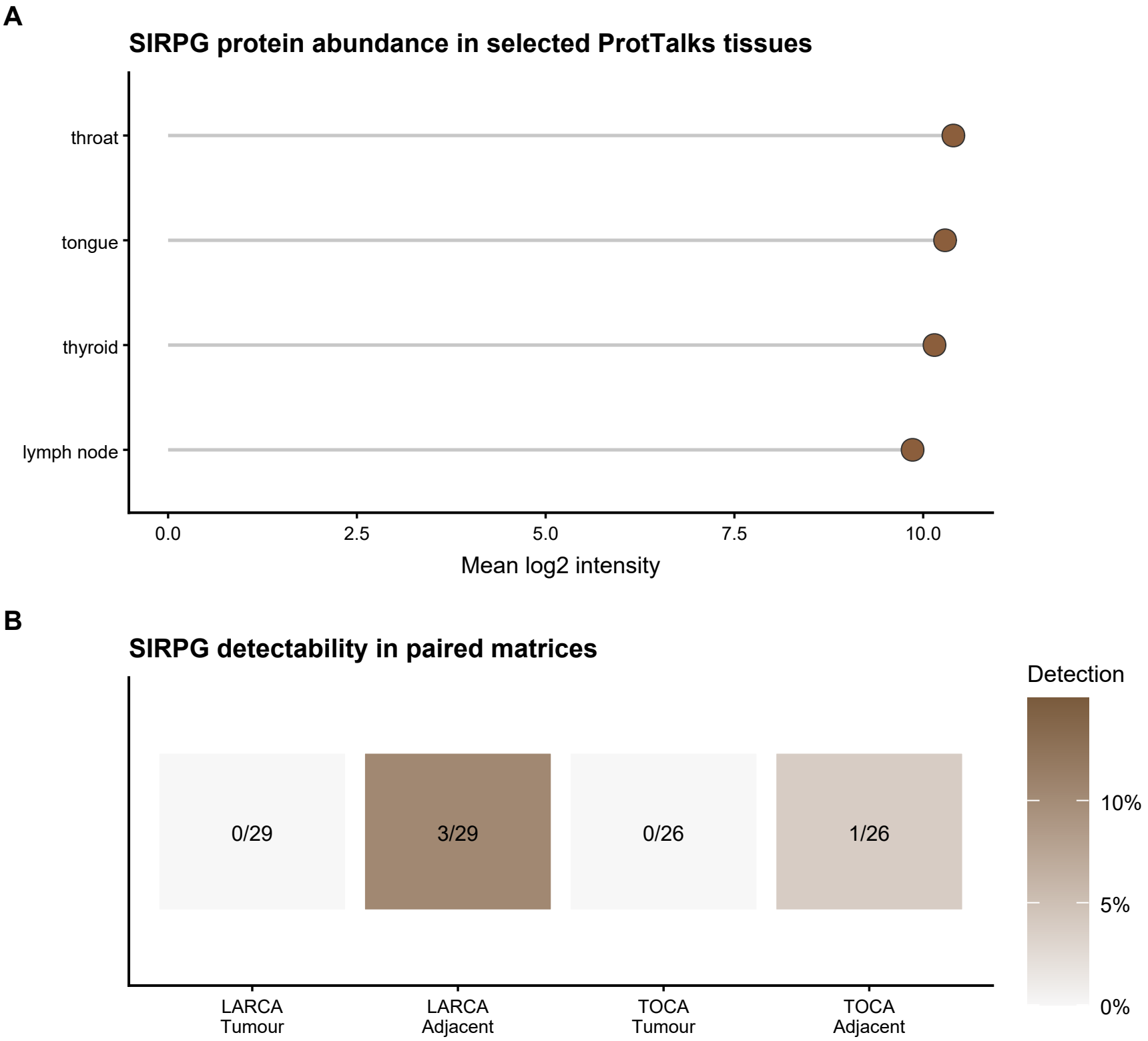

Supplement: Supplementary file 2 [file DataSheet10.pdf]
