# Supplementary material for: Multi-omics identification and functional validation of signal regulatory protein gamma as a prognostic biomarker and immune regulator in head and neck squamous cell carcinoma
Source: Front Immunol. 2026 Aug 21;17:1835156. doi: 10.3389/fimmu.2026.1835156 (PMC13541685; doi:10.3389/fimmu.2026.1835156)
Supplement: Supplementary file 3 [file DataSheet2.pdf]

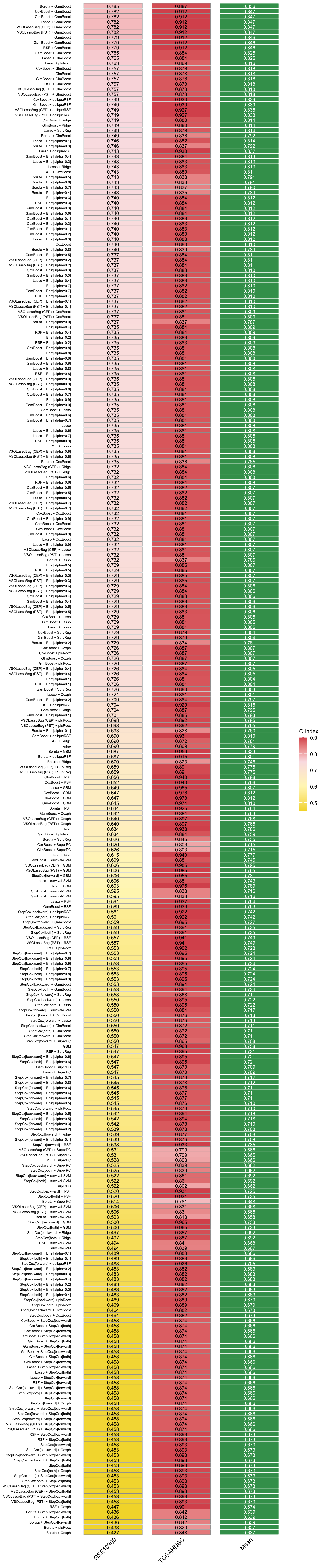
C-index
0.9
0.8
0.7
0.6
0.5
GSE10300
TCGAHNSC
Mean

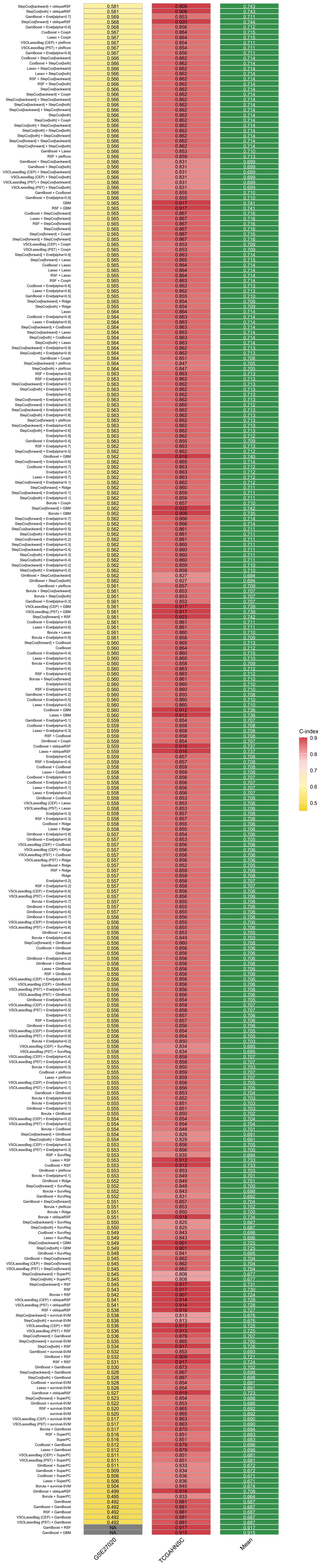
C-index
0.9
0.8
0.7
0.6
0.5
GSE27020
TCGAHNSC
Mean

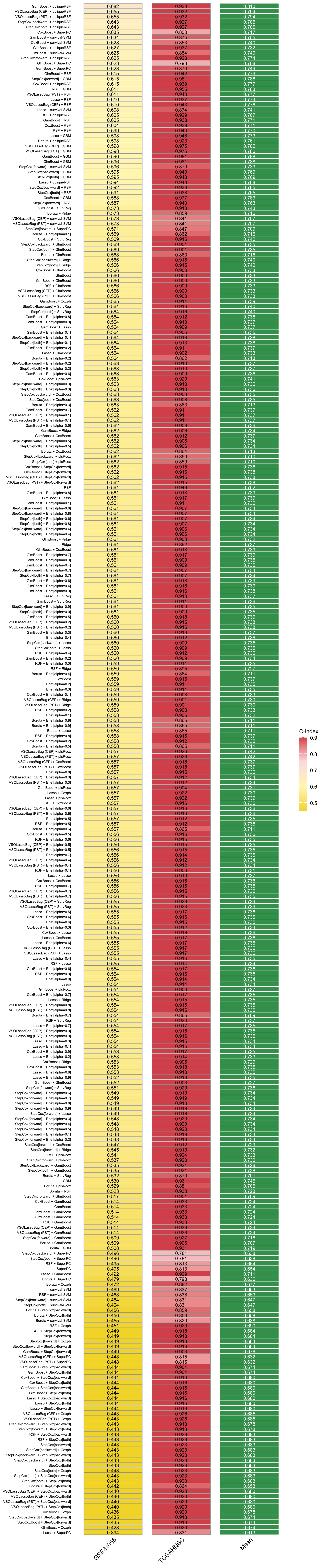
GSE31056
TCGAHNSC
Mean
C-index
0.9
0.8
0.7
0.6
0.5

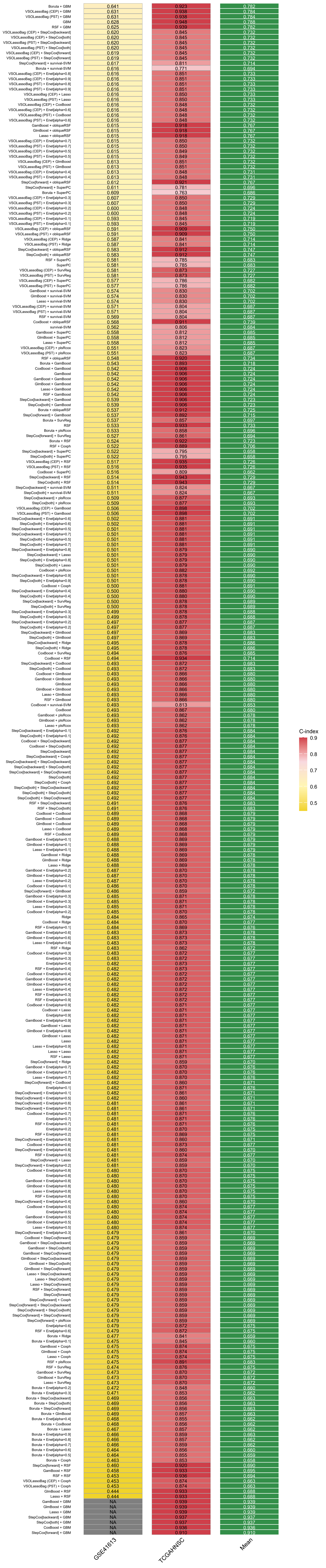
GSE41613
TCGAHNSC
Mean
C-index
0.9
0.8
0.7
0.6
0.5

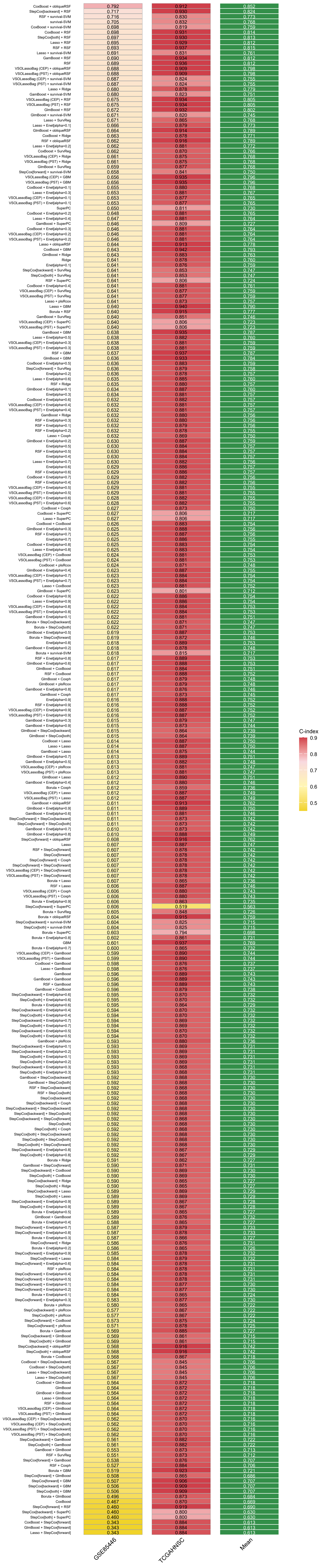
C-index
0.9
0.8
0.7
0.6
0.5
GSE85446
TCGA-HNSC
Mean
