# Supplementary material for: Multi-omics identification and functional validation of signal regulatory protein gamma as a prognostic biomarker and immune regulator in head and neck squamous cell carcinoma
Source: Front Immunol. 2026 Aug 21;17:1835156. doi: 10.3389/fimmu.2026.1835156 (PMC13541685; doi:10.3389/fimmu.2026.1835156)
Supplement: Supplementary file 4 [file DataSheet3.pdf]

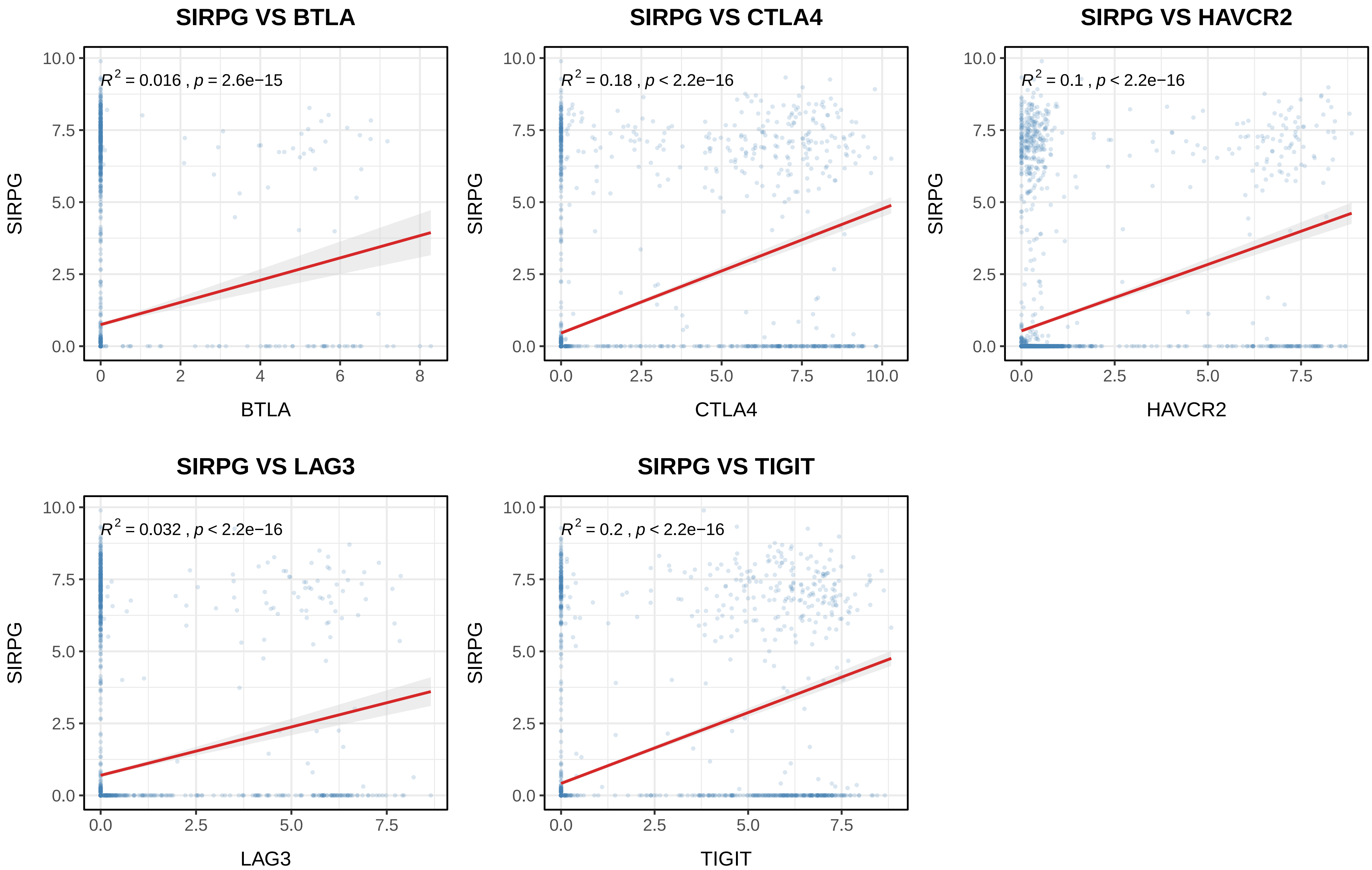
SIRPG VS BTLA
R^2 = 0.016 , p = 2.6e−15
SIRPG
BTLA
SIRPG VS CTLA4
R^2 = 0.18 , p < 2.2e−16
SIRPG
CTLA4
SIRPG VS HAVCR2
R^2 = 0.1 , p < 2.2e−16
SIRPG
HAVCR2
SIRPG VS LAG3
R^2 = 0.032 , p < 2.2e−16
SIRPG
LAG3
SIRPG VS TIGIT
R^2 = 0.2 , p < 2.2e−16
SIRPG
TIGIT

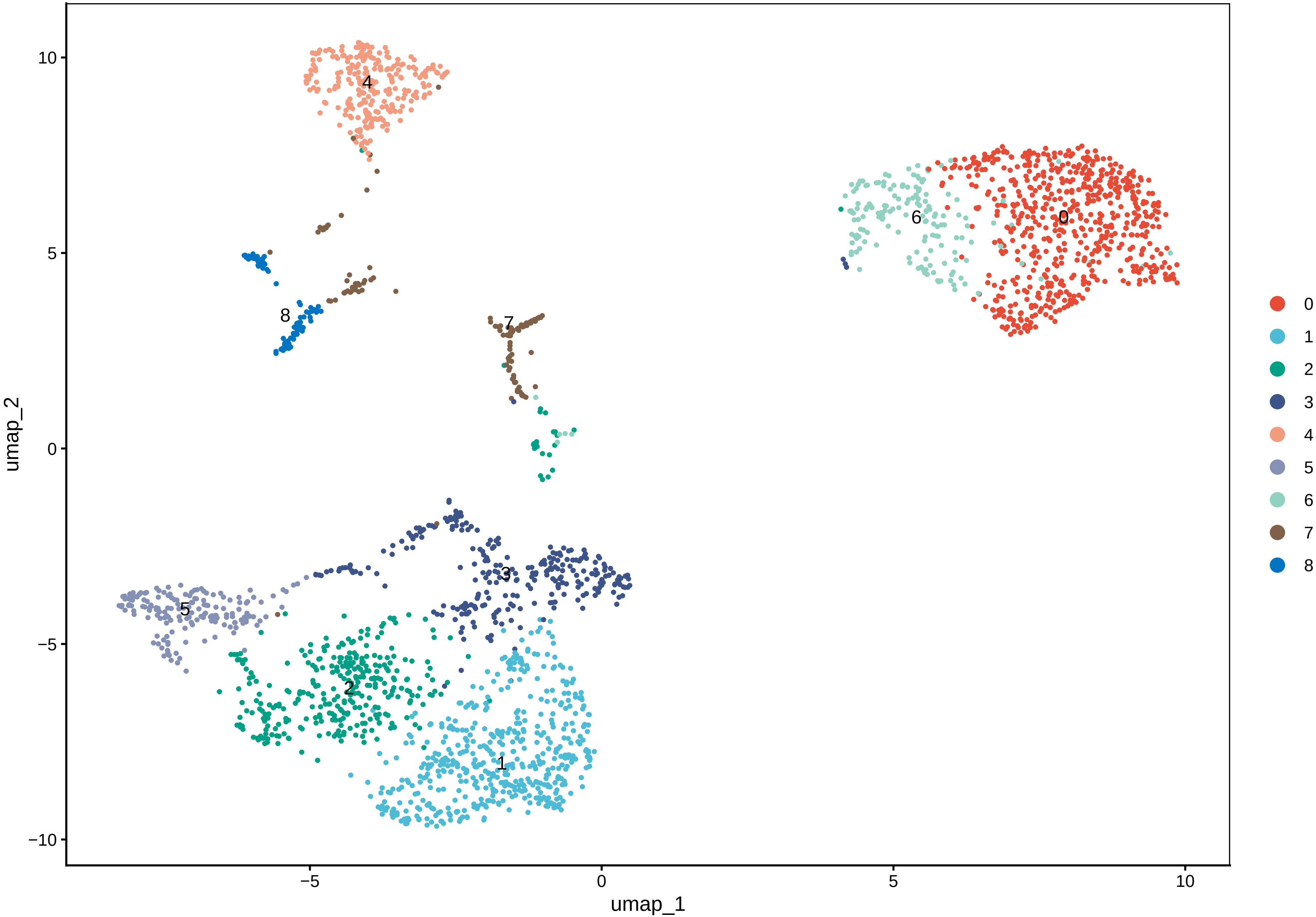

umap_2
umap_1
10
5
0
−5
−10
−5
0
5
10
0
1
2
3
4
5
6
7
8

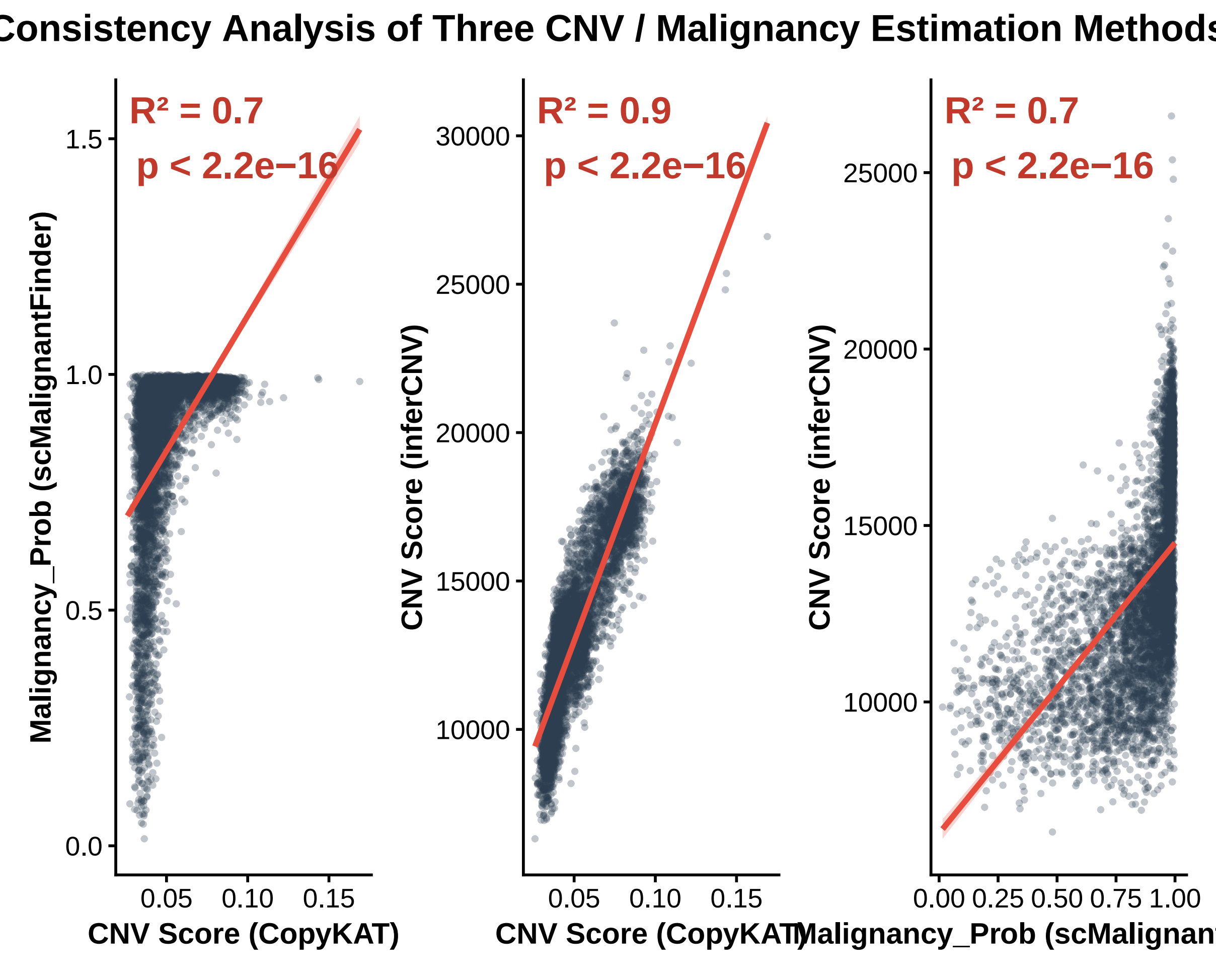
Consistency Analysis of Three CNV / Malignancy Estimation Methods
R² = 0.7
p < 2.2e−16
Malignancy_Prob (scMalignantFinder)
1.5
1.0
0.5
0.0
0.05
0.10
0.15
CNV Score (CopyKAT)
R² = 0.9
p < 2.2e−16
CNV Score (inferCNV)
30000
25000
20000
15000
10000
0.05
0.10
0.15
CNV Score (CopyKAT)
R² = 0.7
p < 2.2e−16
CNV Score (inferCNV)
25000
20000
15000
10000
0.00
0.25
0.50
0.75
1.00
Malignancy_Prob (scMalignant

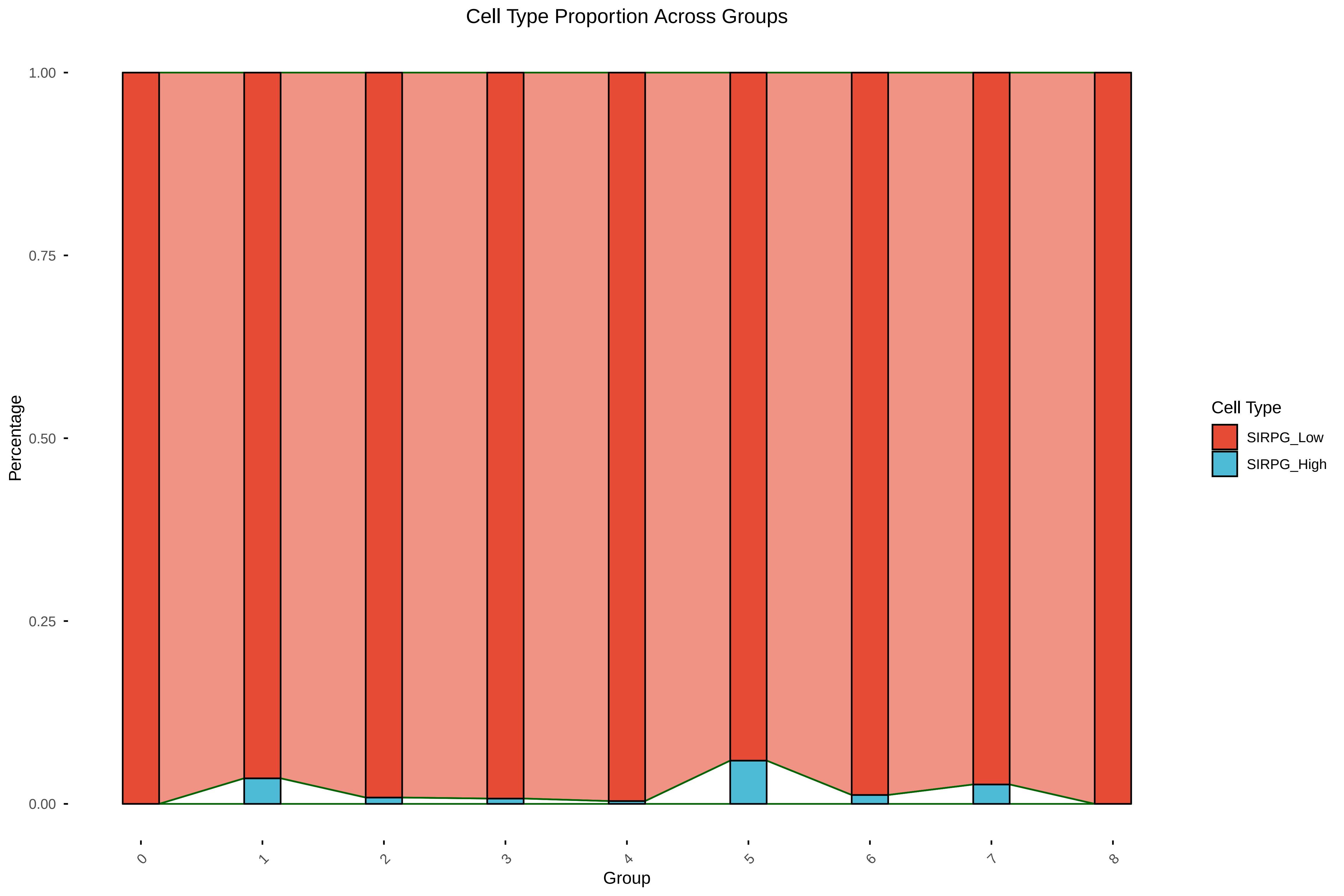
Cell Type Proportion Across Groups
1.00
0.75
0.50
0.25
0.00
Percentage
0
1
2
3
4
5
6
7
8
Group
Cell Type
SIRPG_Low
SIRPG_High

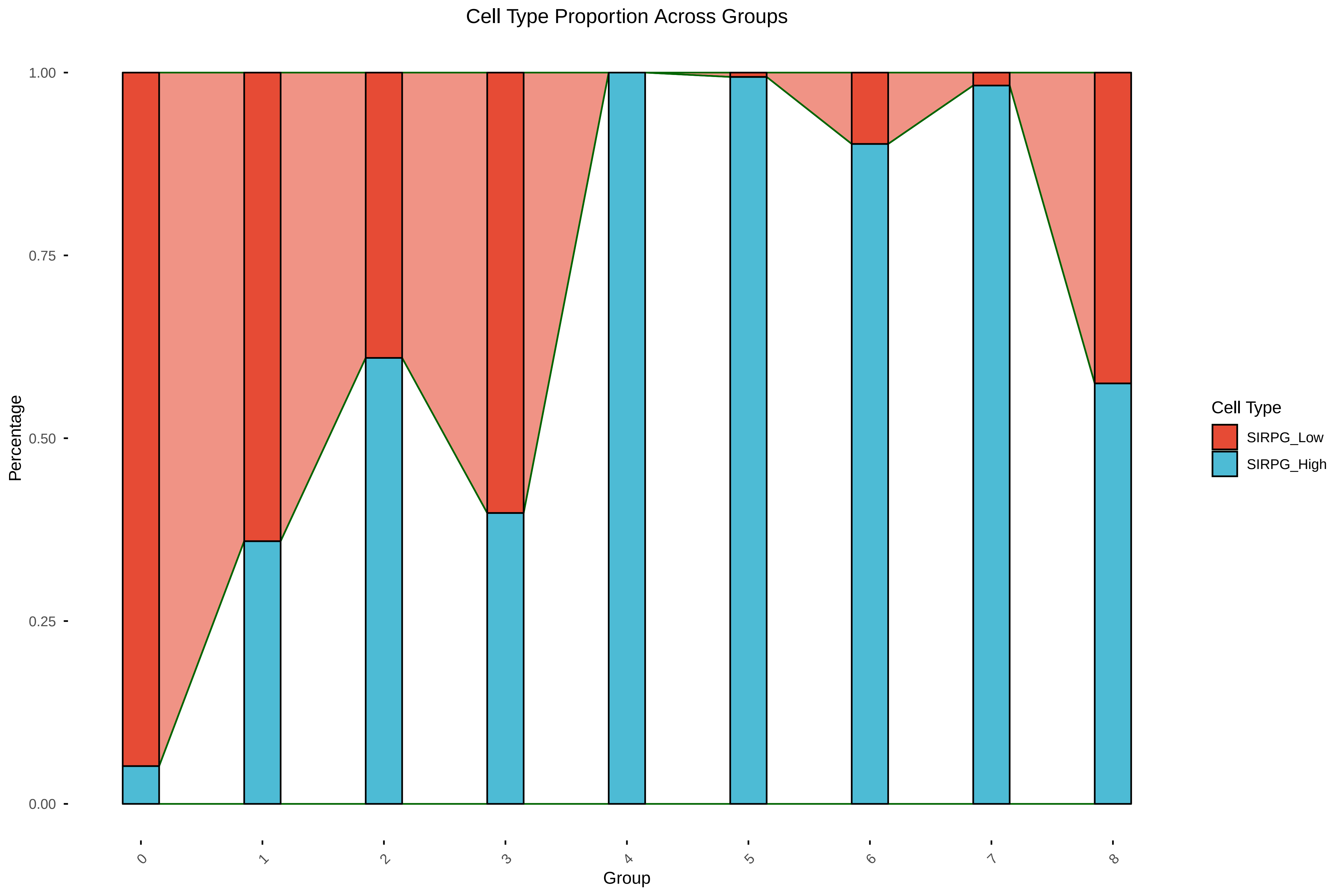
Cell Type Proportion Across Groups
Percentage
1.00
0.75
0.50
0.25
0.00
0
1
2
3
4
5
6
7
8
Group
Cell Type
SIRPG_Low
SIRPG_High

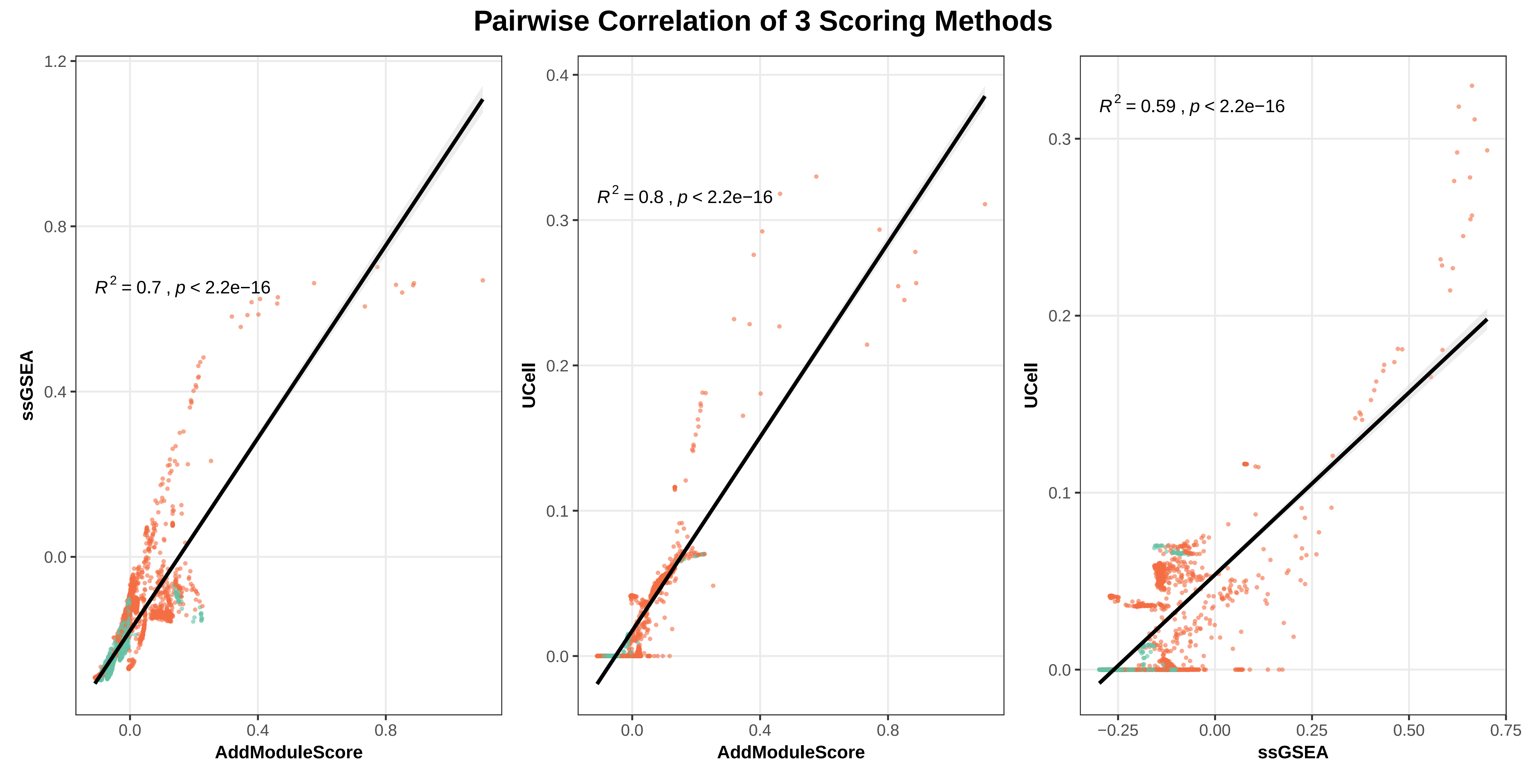

Pairwise Correlation of 3 Scoring Methods
R^2 = 0.7 , p < 2.2e−16
ssGSEA
AddModuleScore
1.2
0.8
0.4
0.0
0.0
0.4
0.8
R^2 = 0.8 , p < 2.2e−16
UCell
AddModuleScore
0.4
0.3
0.2
0.1
0.0
0.0
0.4
0.8
R^2 = 0.59 , p < 2.2e−16
UCell
ssGSEA
0.3
0.2
0.1
0.0
−0.25
0.00
0.25
0.50
0.75

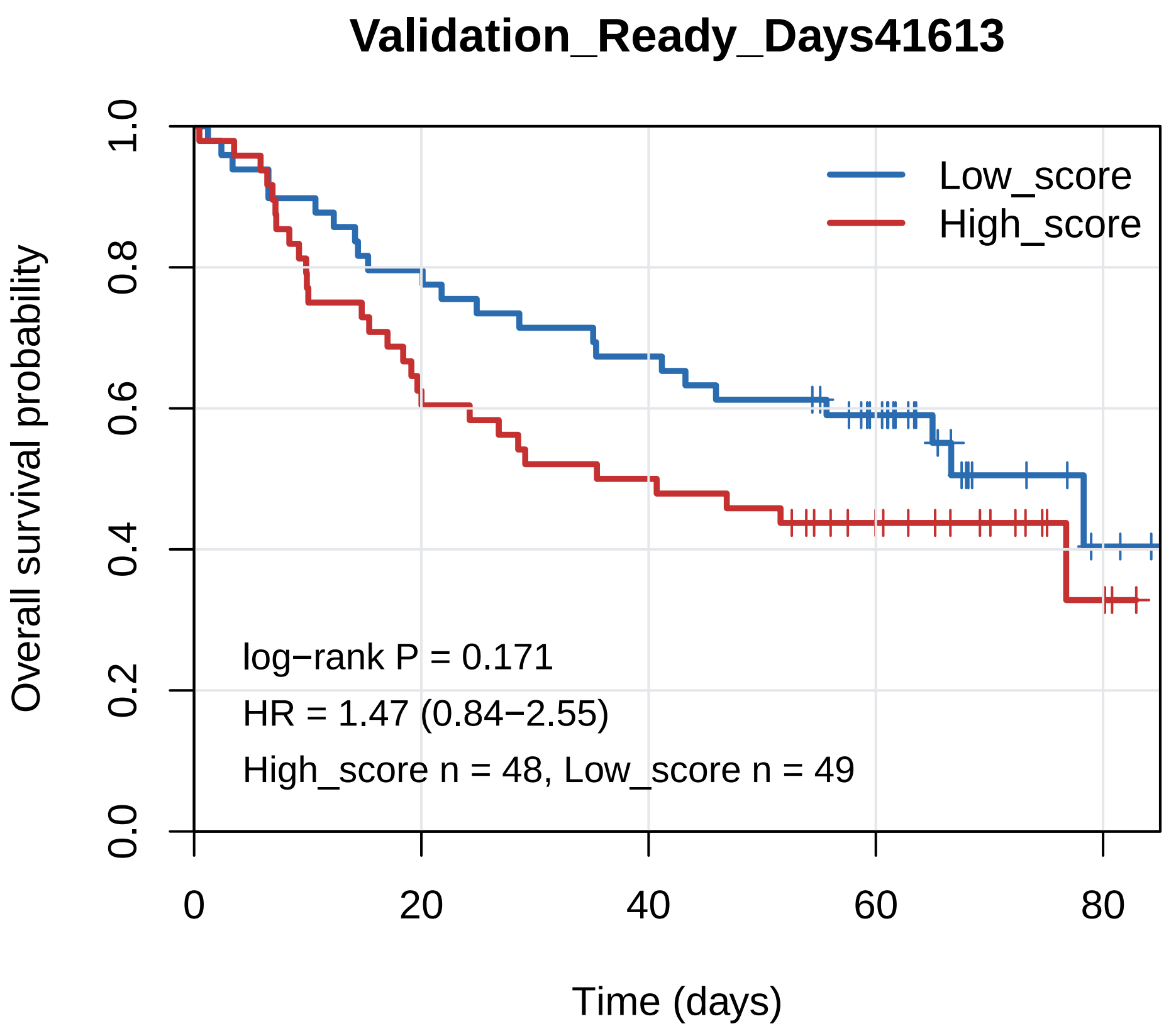
Validation_Ready_Days41613
Overall survival probability
1.0
0.8
0.6
0.4
0.2
0.0
0
20
40
60
80
Time (days)
Low_score
High_score
log−rank P = 0.171
HR = 1.47 (0.84−2.55)
High_score n = 48, Low_score n = 49

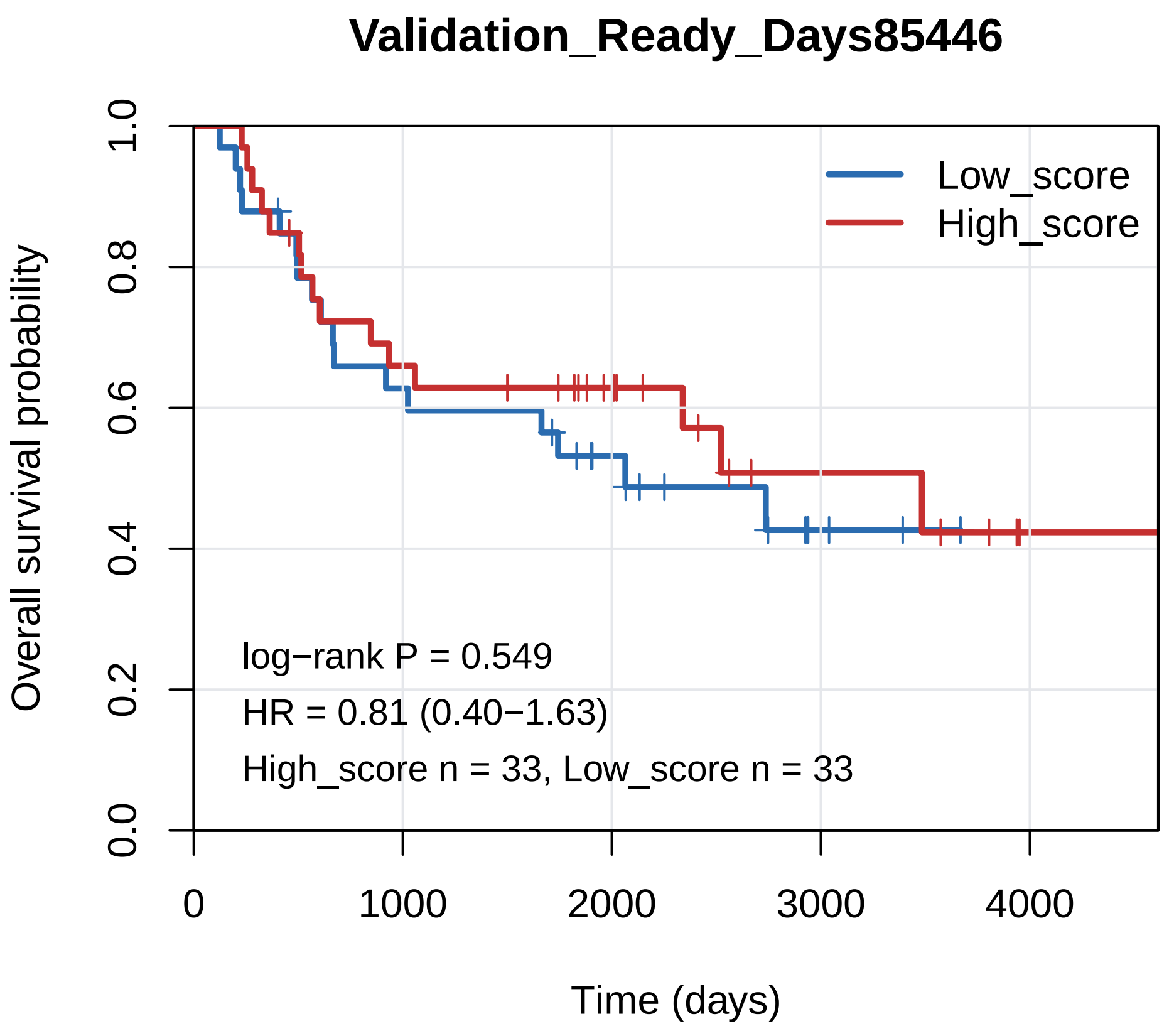
Validation_Ready_Days85446
Low_score
High_score
log−rank P = 0.549
HR = 0.81 (0.40−1.63)
High_score n = 33, Low_score n = 33
Overall survival probability
0.0
0.2
0.4
0.6
0.8
1.0
0
1000
2000
3000
4000
Time (days)

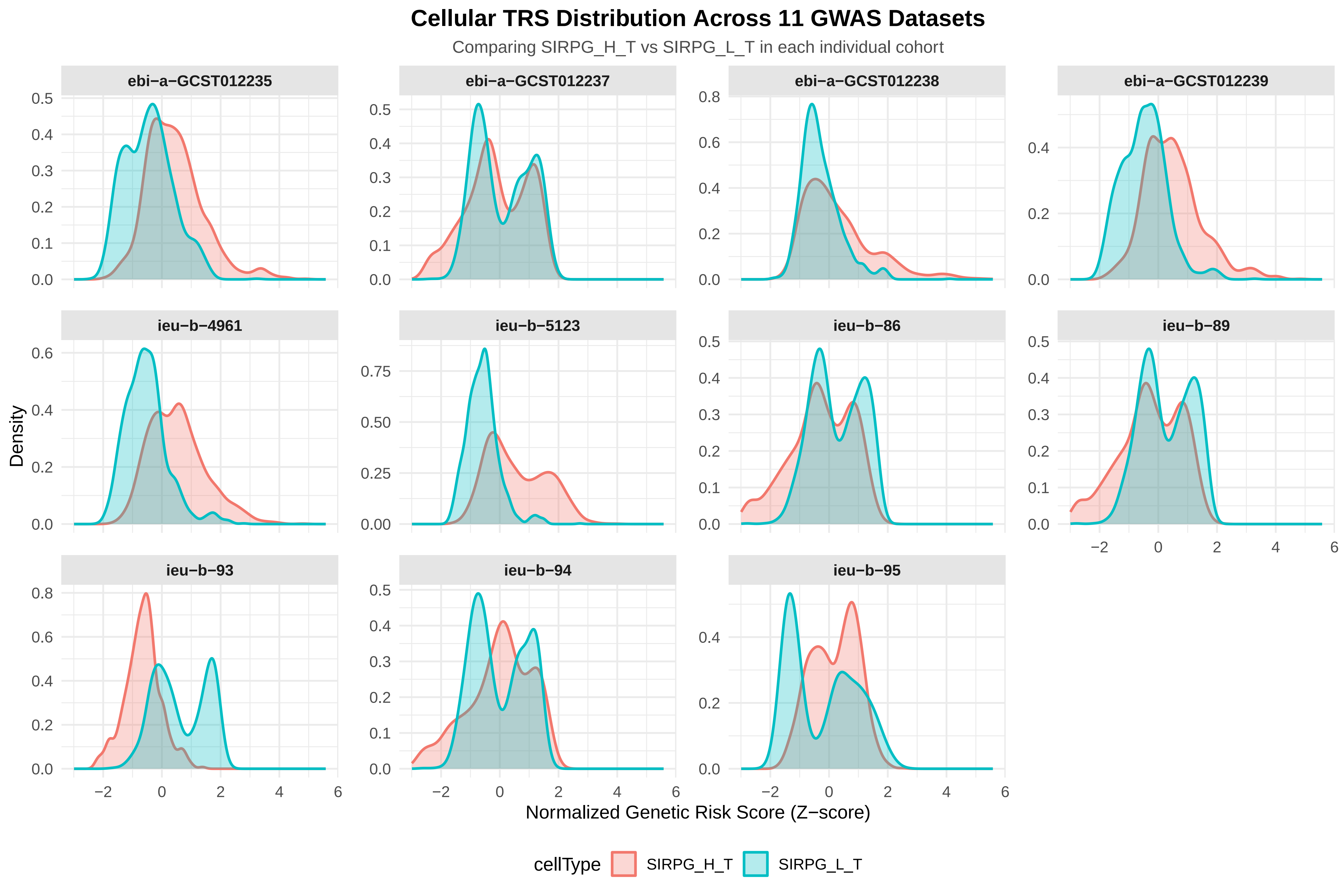
Cellular TRS Distribution Across 11 GWAS Datasets
Comparing SIRPG_H_T vs SIRPG_L_T in each individual cohort
ebi−a−GCST012235
ebi−a−GCST012237
ebi−a−GCST012238
ebi−a−GCST012239
ieu−b−4961
ieu−b−5123
ieu−b−86
ieu−b−89
ieu−b−93
ieu−b−94
ieu−b−95
Density
Normalized Genetic Risk Score (Z−score)
cellType
SIRPG_H_T
SIRPG_L_T
