# Supplementary material for: Multi-omics identification and functional validation of signal regulatory protein gamma as a prognostic biomarker and immune regulator in head and neck squamous cell carcinoma
Source: Front Immunol. 2026 Aug 21;17:1835156. doi: 10.3389/fimmu.2026.1835156 (PMC13541685; doi:10.3389/fimmu.2026.1835156)
Supplement: Supplementary file 5 [file DataSheet4.pdf]

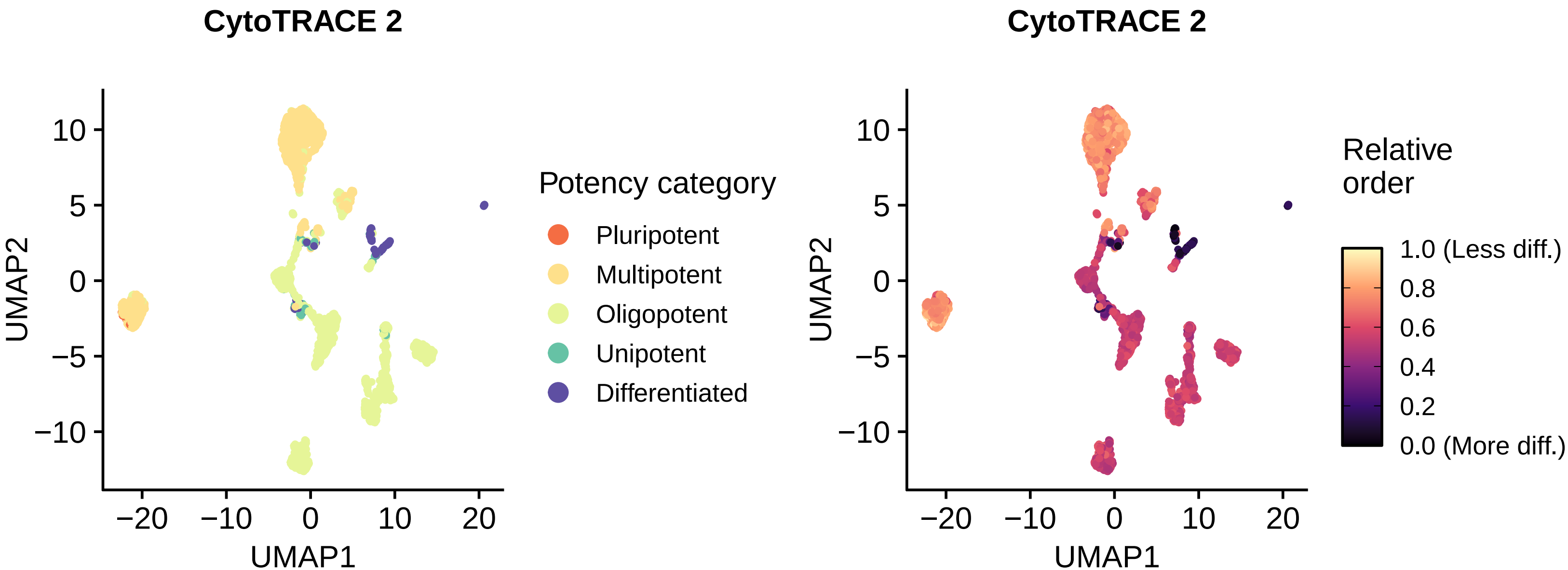

CytoTRACE 2
UMAP2
UMAP1
10
5
0
−5
−10
−20
−10
0
10
20
Potency category
Pluripotent
Multipotent
Oligopotent
Unipotent
Differentiated
CytoTRACE 2
Relative order
1.0 (Less diff.)
0.8
0.6
0.4
0.2
0.0 (More diff.)

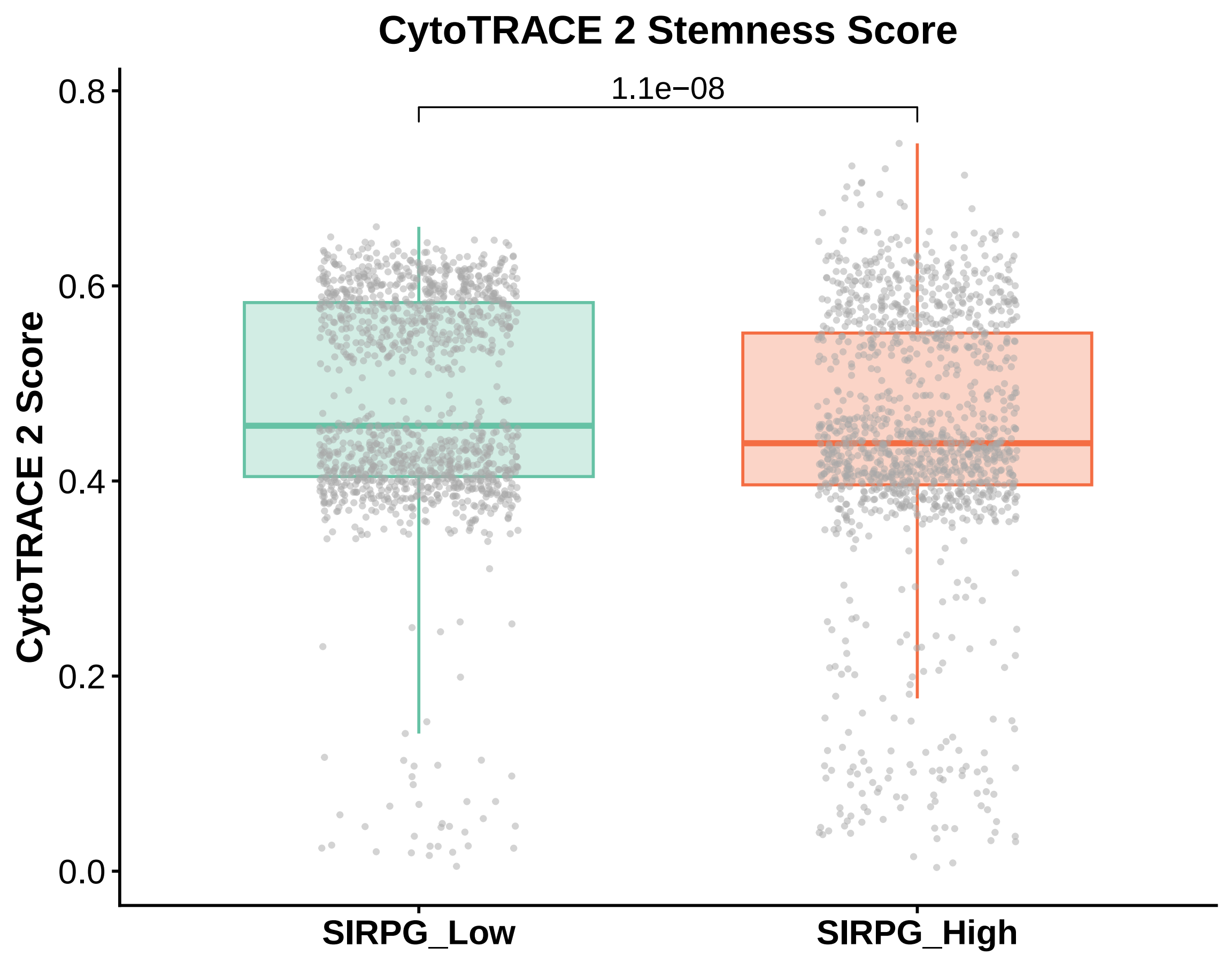
CytoTRACE 2 Stemness Score
1.1e−08
CytoTRACE 2 Score
0.0
0.2
0.4
0.6
0.8
SIRPG_Low
SIRPG_High

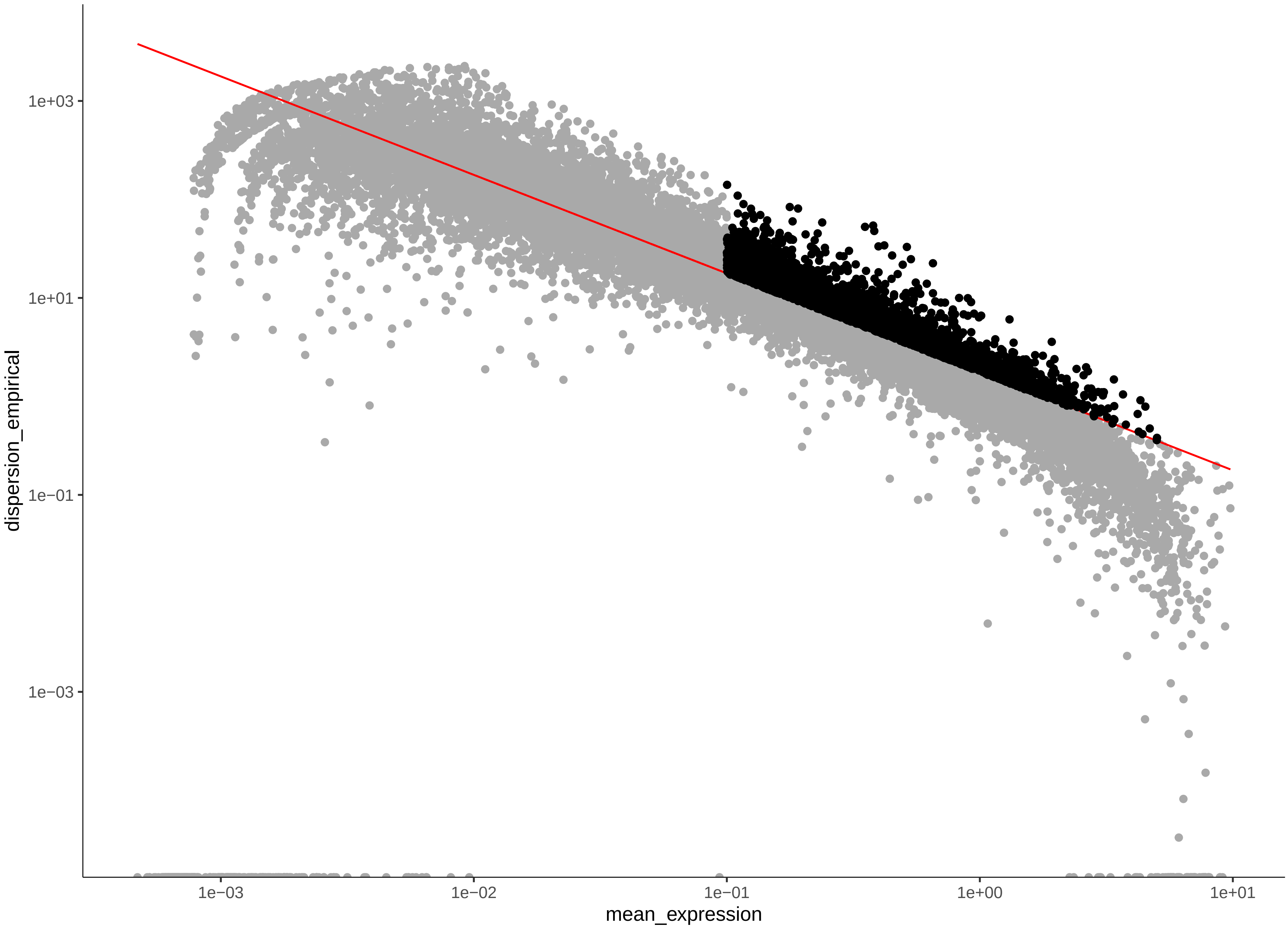
1e+03
1e+01
1e-01
1e-03
dispersion_empirical
1e-03
1e-02
1e-01
1e+00
1e+01
mean_expression

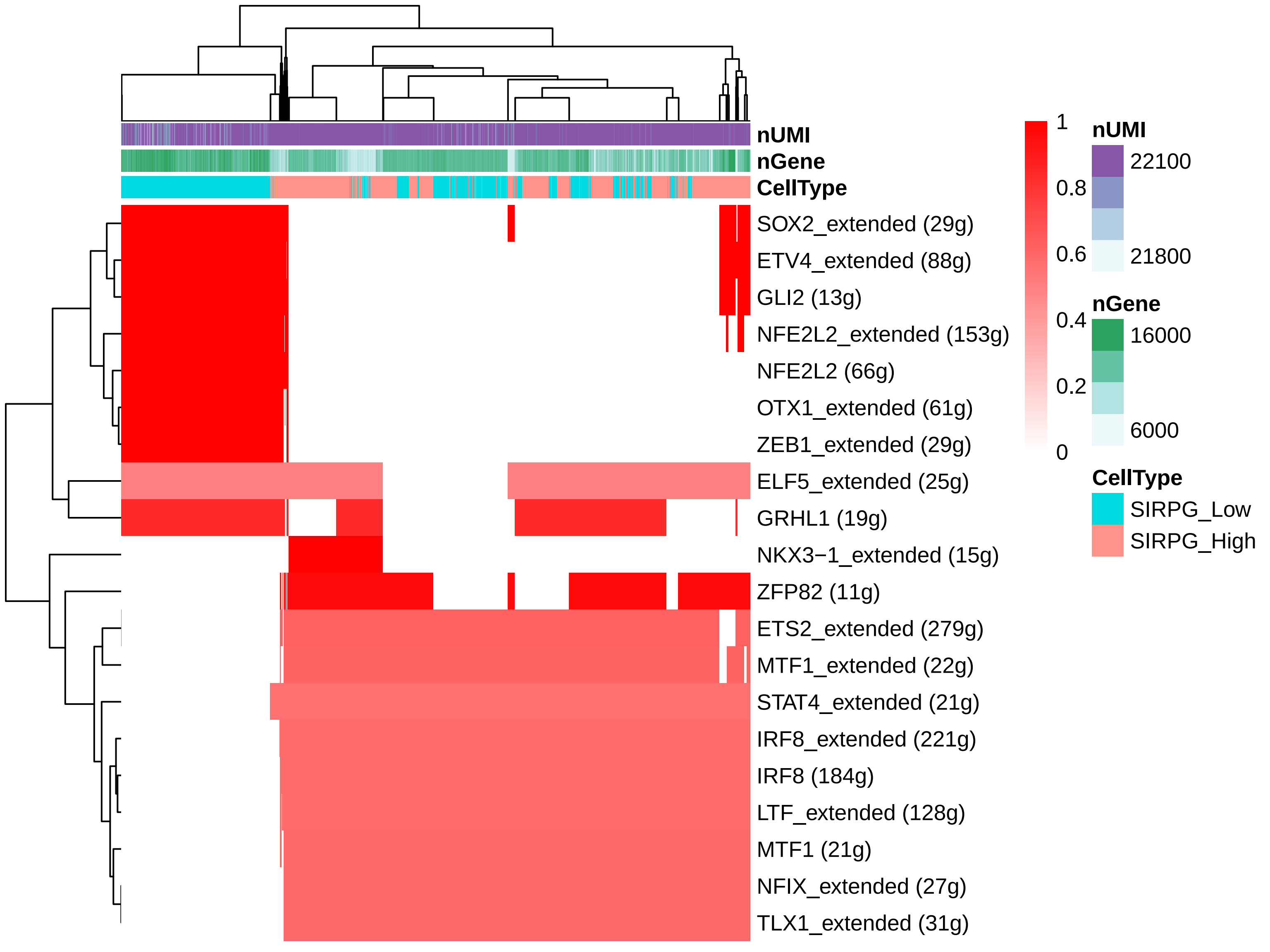
nUMI
nGene
CellType
SOX2_extended (29g)
ETV4_extended (88g)
GLI2 (13g)
NFE2L2_extended (153g)
NFE2L2 (66g)
OTX1_extended (61g)
ZEB1_extended (29g)
ELF5_extended (25g)
GRHL1 (19g)
NKX3−1_extended (15g)
ZFP82 (11g)
ETS2_extended (279g)
MTF1_extended (22g)
STAT4_extended (21g)
IRF8_extended (221g)
IRF8 (184g)
LTF_extended (128g)
MTF1 (21g)
NFIX_extended (27g)
TLX1_extended (31g)
1
0.8
0.6
0.4
0.2
0
nUMI
22100
21800
nGene
16000
6000
CellType
SIRPG_Low
SIRPG_High

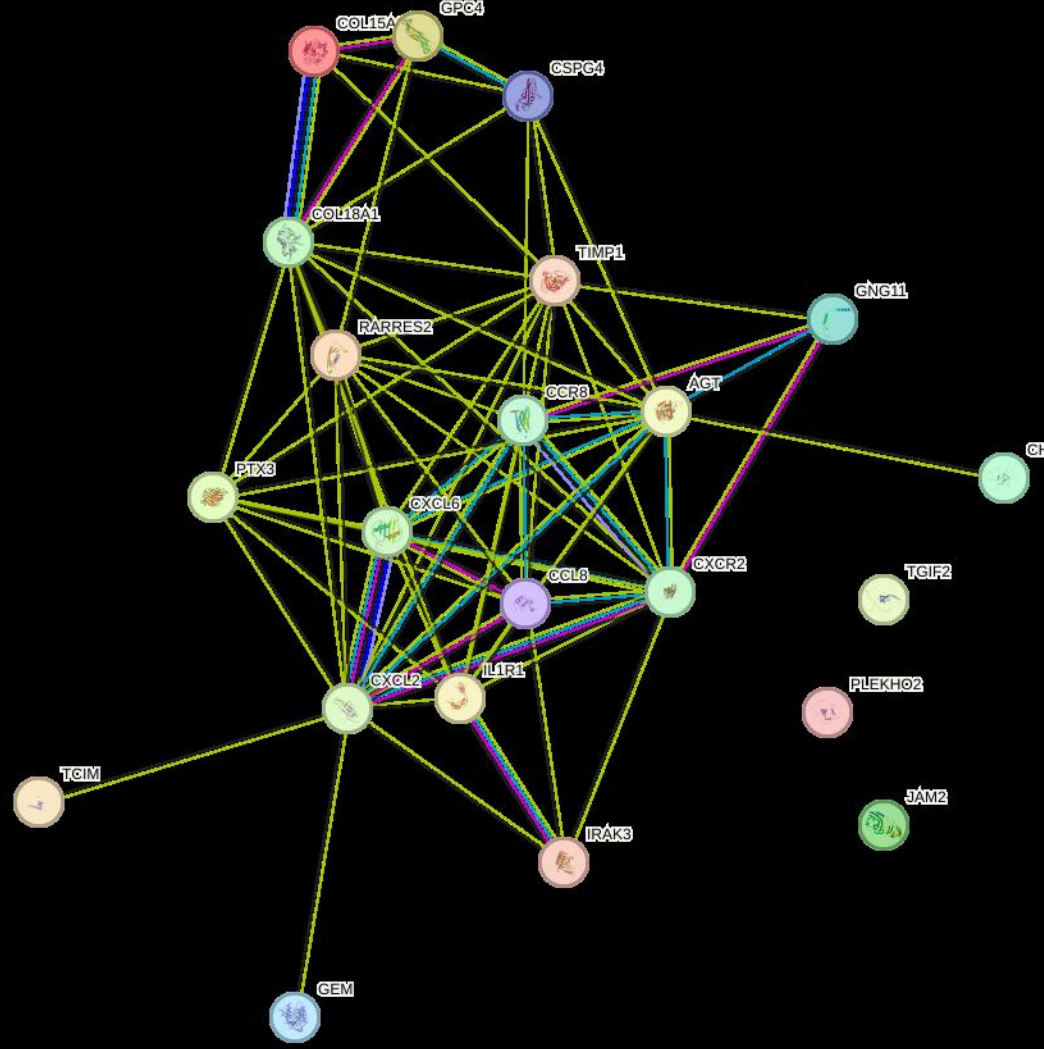
COL15A1
GPC4
CSPG4
COL18A1
TIMP1
GNG11
RARRES2
CCR8
AGT
CHGB
PTX3
CXCL6
CCL8
CXCR2
TGIF2
CXCL2
IL1R1
PLEKHO2
TCIM
JAM2
IRAK3
GEM

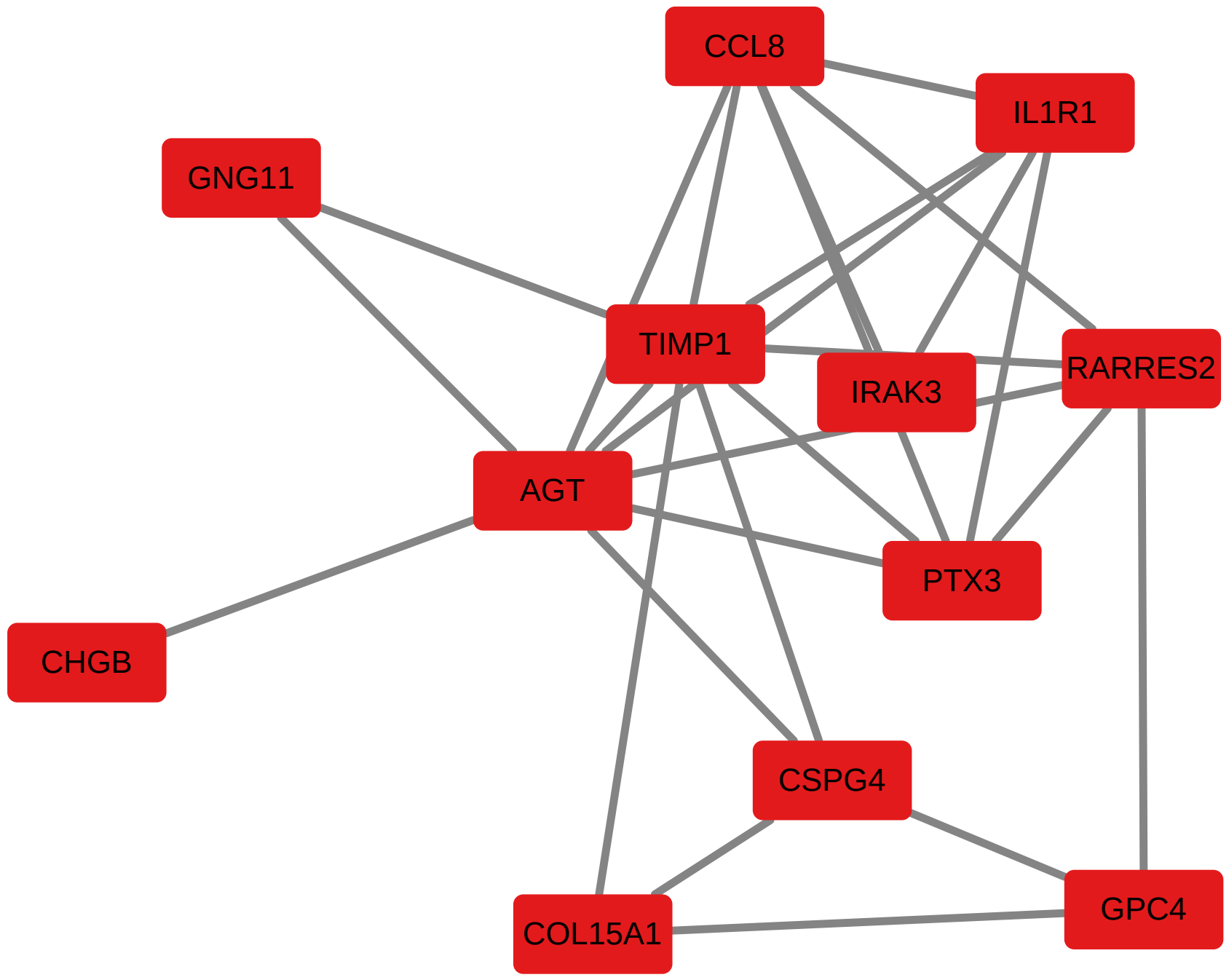
CCL8
IL1R1
GNG11
TIMP1
IRAK3
RARRES2
AGT
PTX3
CHGB
CSPG4
GPC4
COL15A1

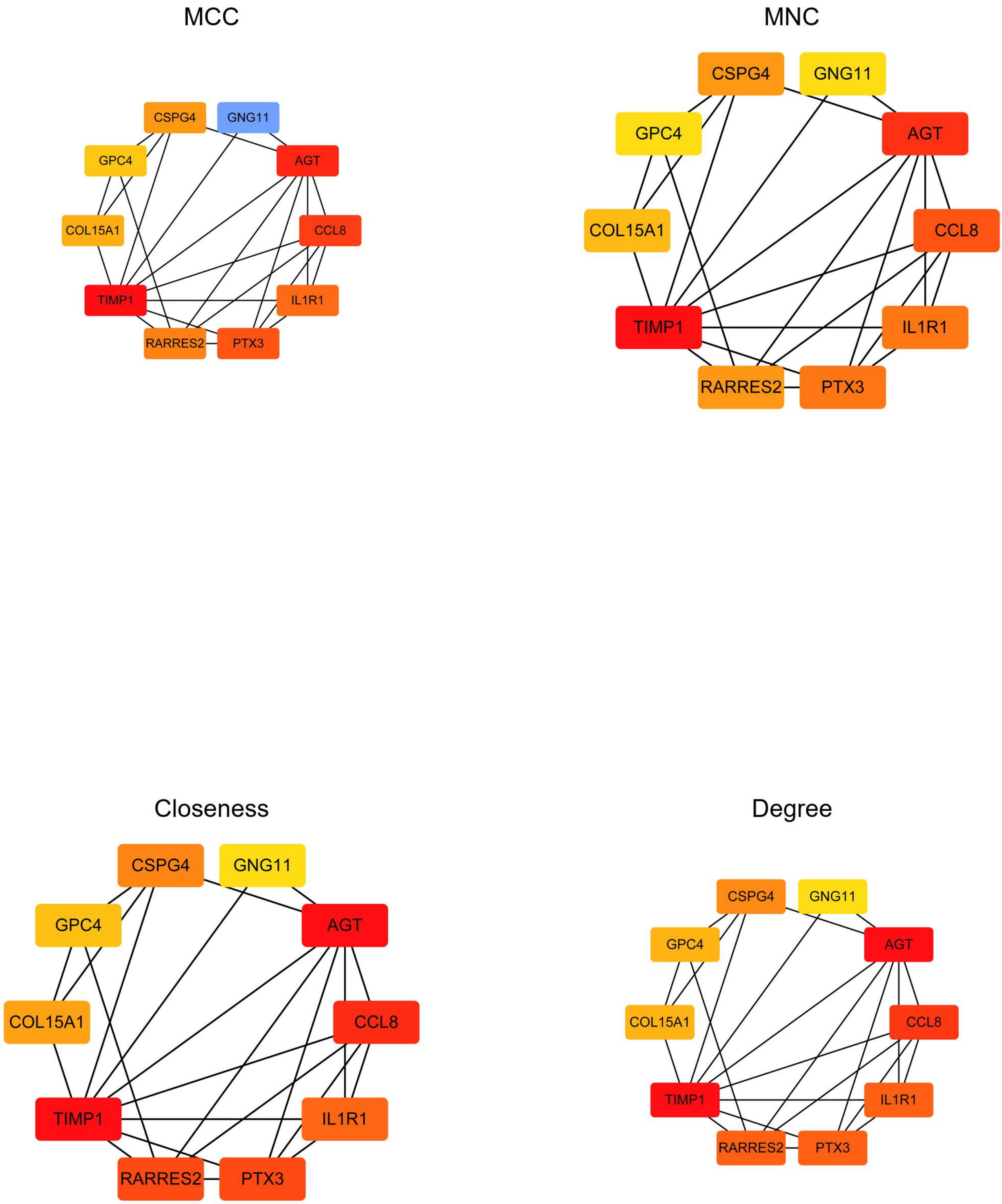
MCC
CSPG4
GNG11
GPC4
AGT
COL15A1
CCL8
TIMP1
IL1R1
RARRES2
PTX3
MNC
CSPG4
GNG11
GPC4
AGT
COL15A1
CCL8
TIMP1
IL1R1
RARRES2
PTX3
Closeness
CSPG4
GNG11
GPC4
AGT
COL15A1
CCL8
TIMP1
IL1R1
RARRES2
PTX3
Degree
CSPG4
GNG11
GPC4
AGT
COL15A1
CCL8
TIMP1
IL1R1
RARRES2
PTX3
