# Supplementary material for: Multi-omics identification and functional validation of signal regulatory protein gamma as a prognostic biomarker and immune regulator in head and neck squamous cell carcinoma
Source: Front Immunol. 2026 Aug 21;17:1835156. doi: 10.3389/fimmu.2026.1835156 (PMC13541685; doi:10.3389/fimmu.2026.1835156)
Supplement: Supplementary file 6 [file DataSheet5.pdf]

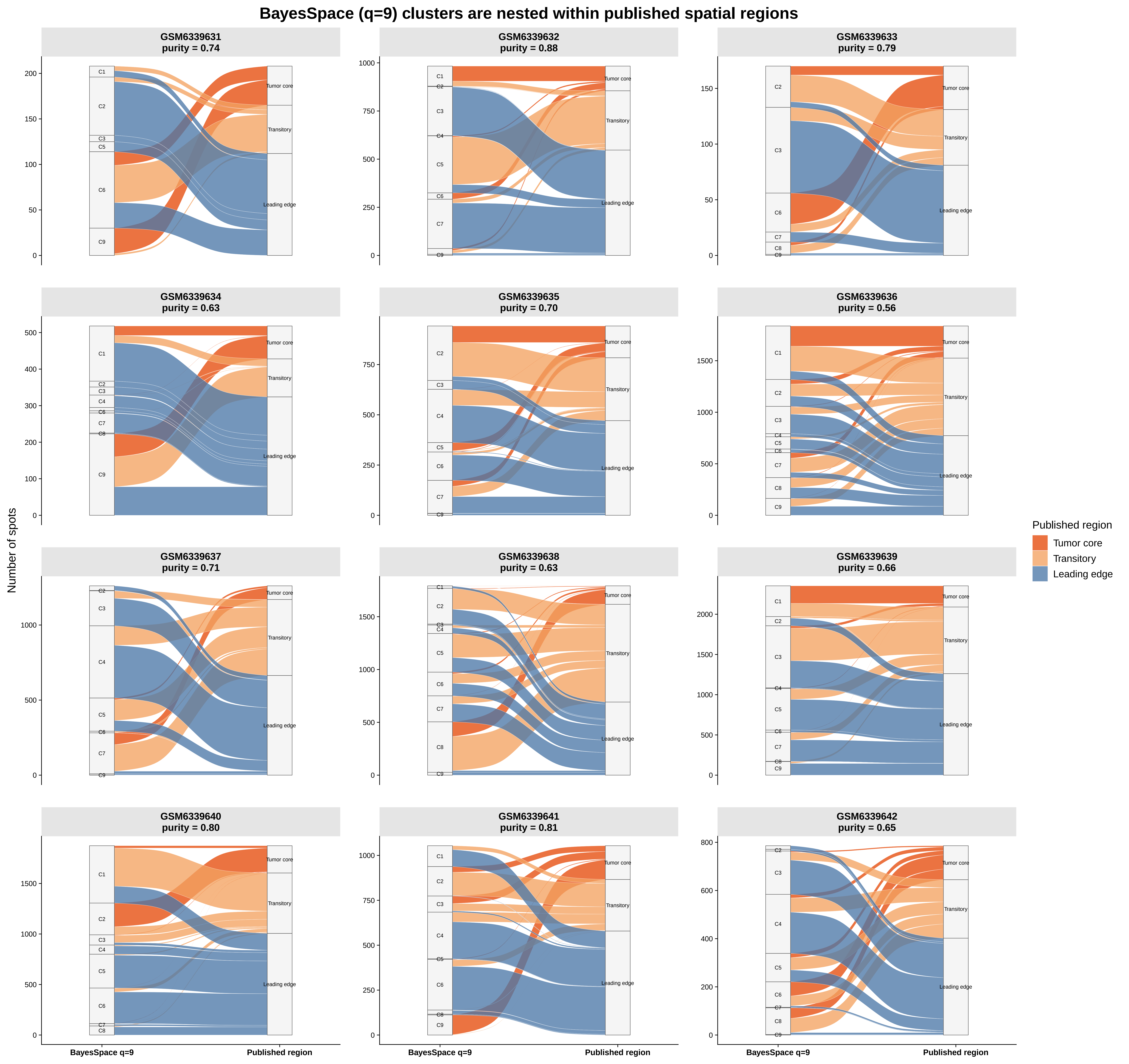
BayesSpace (q=9) clusters are nested within published spatial regions
GSM6339631 purity = 0.74
GSM6339632 purity = 0.88
GSM6339633 purity = 0.79
GSM6339634 purity = 0.63
GSM6339635 purity = 0.70
GSM6339636 purity = 0.56
GSM6339637 purity = 0.71
GSM6339638 purity = 0.63
GSM6339639 purity = 0.66
GSM6339640 purity = 0.80
GSM6339641 purity = 0.81
GSM6339642 purity = 0.65
Number of spots
BayesSpace q=9
Published region
Tumor core
Transitory
Leading edge
Published region
Tumor core
Transitory
Leading edge
