# Supplementary material for: Multi-omics identification and functional validation of signal regulatory protein gamma as a prognostic biomarker and immune regulator in head and neck squamous cell carcinoma
Source: Front Immunol. 2026 Aug 21;17:1835156. doi: 10.3389/fimmu.2026.1835156 (PMC13541685; doi:10.3389/fimmu.2026.1835156)
Supplement: Supplementary file 7 [file DataSheet6.pdf]

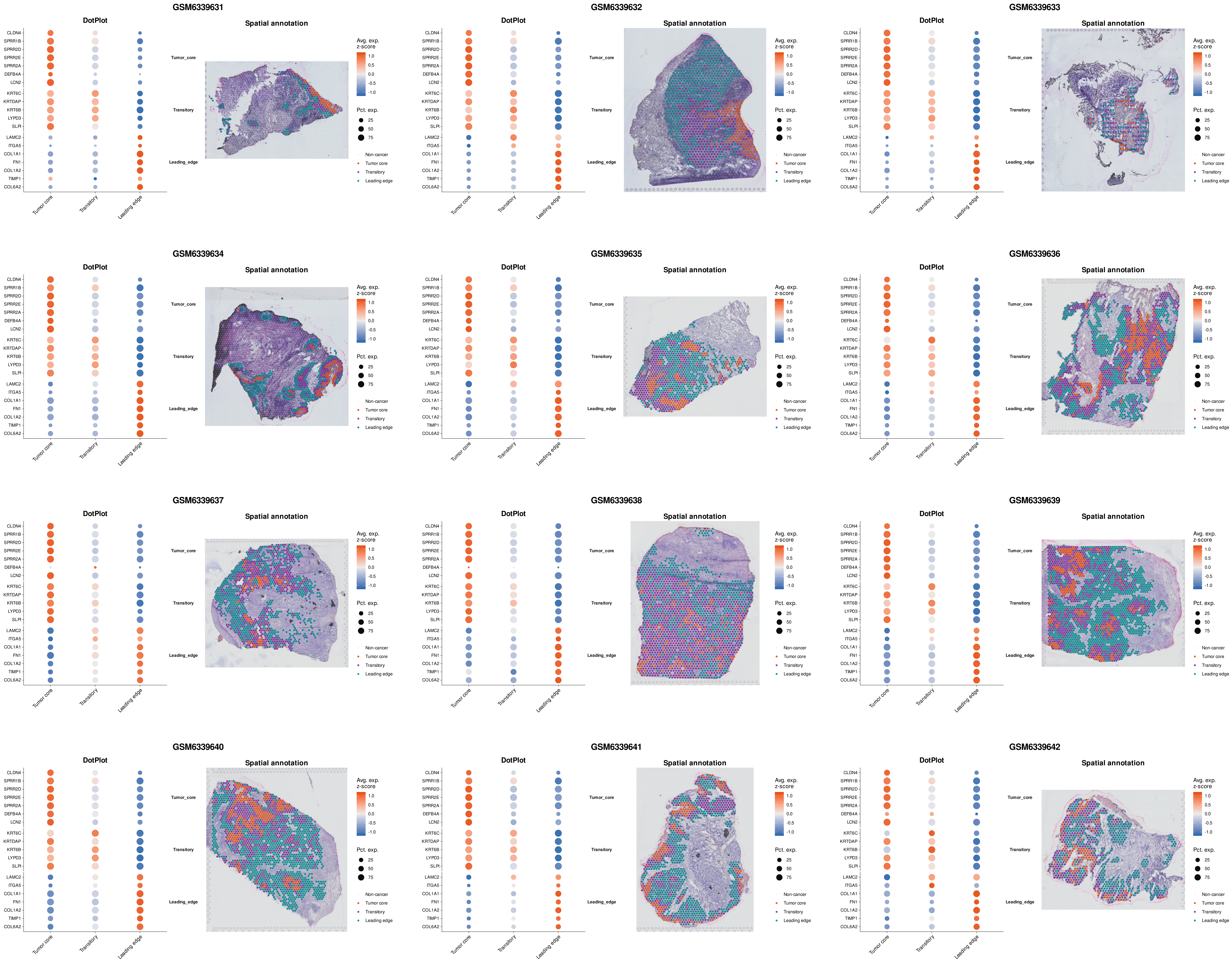

GSM6339631
GSM6339632
GSM6339633
GSM6339634
GSM6339635
GSM6339636
GSM6339637
GSM6339638
GSM6339639
GSM6339640
GSM6339641
GSM6339642
DotPlot
Spatial annotation
CLDN4
SPRR1B
SPRR2D
SPRR2E
SPRR2A
DEFB4A
LCN2
KRT6C
KRTDAP
KRT6B
LYPD3
SLPI
LAMC2
ITGA5
COL1A1
FN1
COL1A2
TIMP1
COL6A2
Tumor core
Transitory
Leading edge
Tumor_core
Transitory
Leading_edge
Avg. exp. z-score
1.0
0.5
0.0
-0.5
-1.0
Pct. exp.
25
50
75
Non-cancer
Tumor core
Transitory
Leading edge
