# Supplementary material for: Multi-omics identification and functional validation of signal regulatory protein gamma as a prognostic biomarker and immune regulator in head and neck squamous cell carcinoma
Source: Front Immunol. 2026 Aug 21;17:1835156. doi: 10.3389/fimmu.2026.1835156 (PMC13541685; doi:10.3389/fimmu.2026.1835156)
Supplement: Supplementary file 8 [file DataSheet7.pdf]

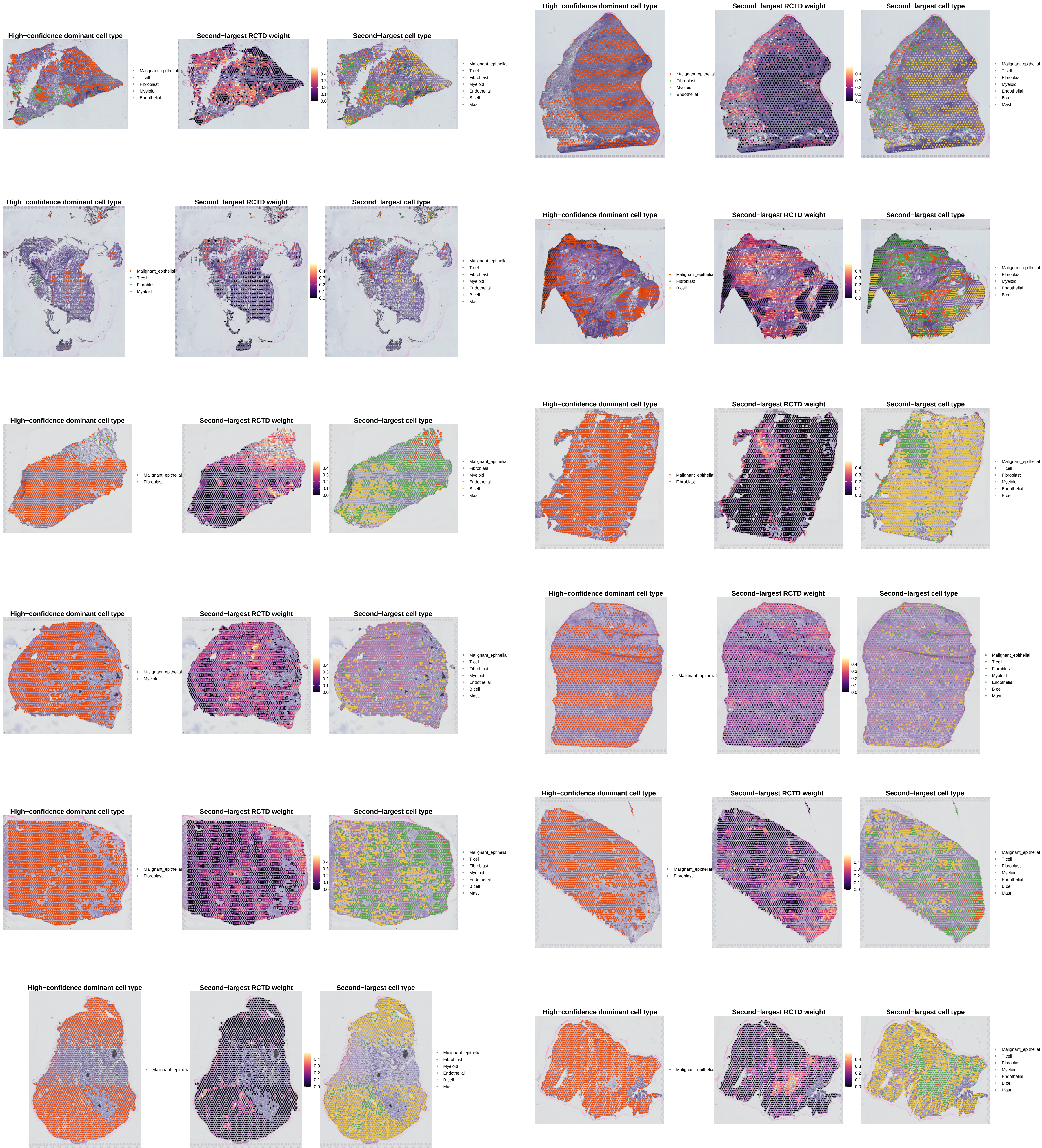

High−confidence dominant cell type
Malignant_epithelial
T cell
Fibroblast
Myeloid
Endothelial
Second−largest RCTD weight
0.4
0.3
0.2
0.1
0.0
Second−largest cell type
Malignant_epithelial
T cell
Fibroblast
Myeloid
Endothelial
B cell
Mast
High−confidence dominant cell type
Malignant_epithelial
Fibroblast
Myeloid
Endothelial
Second−largest RCTD weight
Second−largest cell type
Malignant_epithelial
T cell
Fibroblast
Myeloid
Endothelial
B cell
Mast
High−confidence dominant cell type
Malignant_epithelial
T cell
Fibroblast
Myeloid
Second−largest RCTD weight
Second−largest cell type
Malignant_epithelial
T cell
Fibroblast
Myeloid
Endothelial
B cell
Mast
High−confidence dominant cell type
Malignant_epithelial
Fibroblast
B cell
Second−largest RCTD weight
Second−largest cell type
Malignant_epithelial
Fibroblast
Myeloid
Endothelial
B cell
High−confidence dominant cell type
Malignant_epithelial
Fibroblast
Second−largest RCTD weight
Second−largest cell type
Malignant_epithelial
Fibroblast
Myeloid
Endothelial
B cell
Mast
High−confidence dominant cell type
Malignant_epithelial
Fibroblast
Second−largest RCTD weight
Second−largest cell type
Malignant_epithelial
T cell
Fibroblast
Myeloid
Endothelial
B cell
High−confidence dominant cell type
Malignant_epithelial
Myeloid
Second−largest RCTD weight
Second−largest cell type
Malignant_epithelial
T cell
Fibroblast
Myeloid
Endothelial
B cell
Mast
High−confidence dominant cell type
Malignant_epithelial
Second−largest RCTD weight
Second−largest cell type
Malignant_epithelial
T cell
Fibroblast
Myeloid
Endothelial
B cell
Mast
High−confidence dominant cell type
Malignant_epithelial
Fibroblast
Second−largest RCTD weight
Second−largest cell type
Malignant_epithelial
T cell
Fibroblast
Myeloid
Endothelial
B cell
Mast
High−confidence dominant cell type
Malignant_epithelial
Fibroblast
Second−largest RCTD weight
Second−largest cell type
Malignant_epithelial
T cell
Fibroblast
Myeloid
Endothelial
B cell
Mast
High−confidence dominant cell type
Malignant_epithelial
Second−largest RCTD weight
Second−largest cell type
Malignant_epithelial
Fibroblast
Myeloid
Endothelial
B cell
Mast
High−confidence dominant cell type
Malignant_epithelial
Second−largest RCTD weight
Second−largest cell type
Malignant_epithelial
T cell
Fibroblast
Myeloid
Endothelial
B cell
Mast
