# Supplementary material for: Multi-omics identification and functional validation of signal regulatory protein gamma as a prognostic biomarker and immune regulator in head and neck squamous cell carcinoma
Source: Front Immunol. 2026 Aug 21;17:1835156. doi: 10.3389/fimmu.2026.1835156 (PMC13541685; doi:10.3389/fimmu.2026.1835156)
Supplement: Supplementary file 9 [file DataSheet8.pdf]

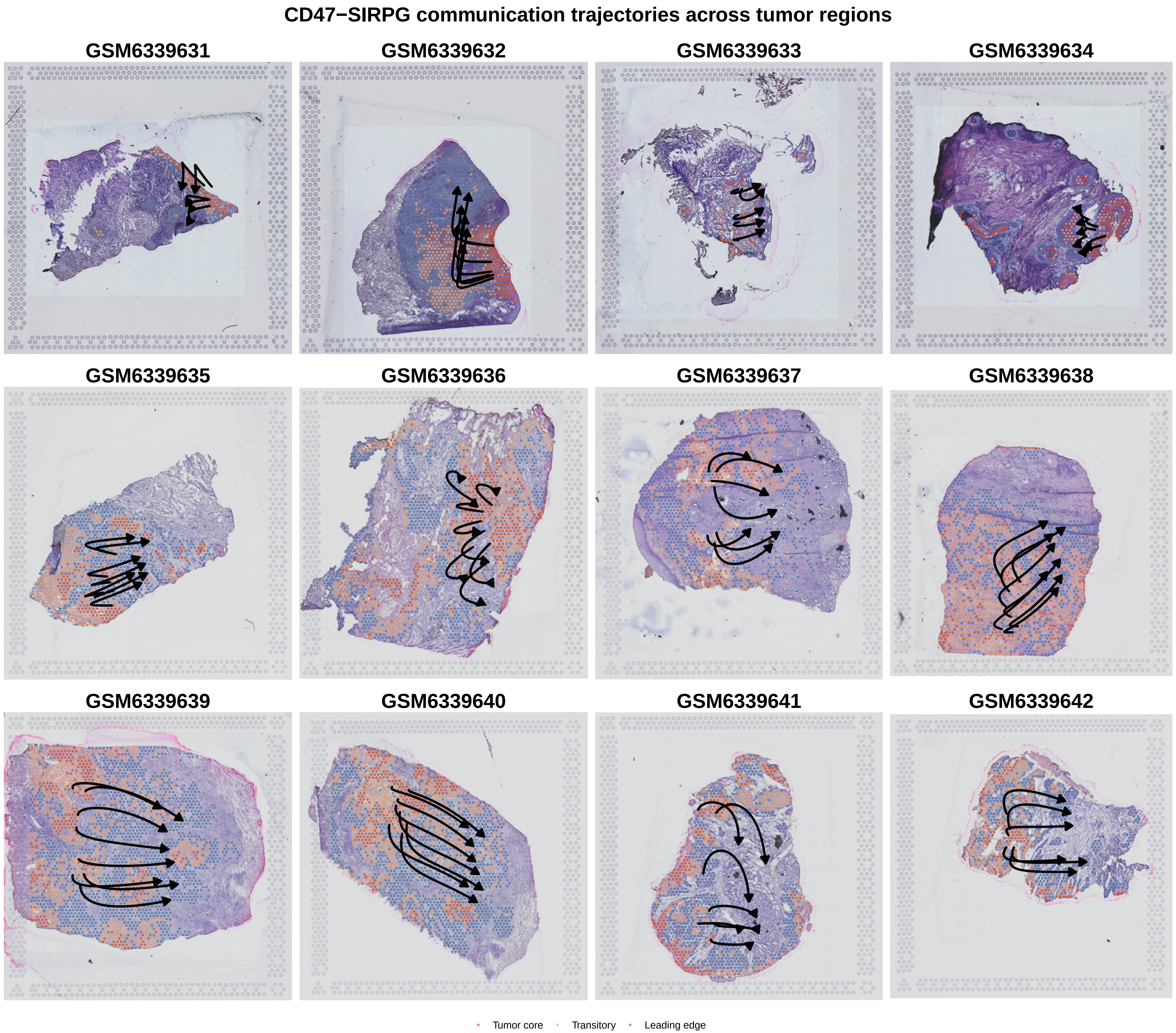
CD47−SIRPG communication trajectories across tumor regions
GSM6339631
GSM6339632
GSM6339633
GSM6339634
GSM6339635
GSM6339636
GSM6339637
GSM6339638
GSM6339639
GSM6339640
GSM6339641
GSM6339642
Tumor core
Transitory
Leading edge
