# Supplementary material for: Multi-omics identification and functional validation of signal regulatory protein gamma as a prognostic biomarker and immune regulator in head and neck squamous cell carcinoma
Source: Front Immunol. 2026 Aug 21;17:1835156. doi: 10.3389/fimmu.2026.1835156 (PMC13541685; doi:10.3389/fimmu.2026.1835156)
Supplement: Supplementary file 10 [file DataSheet9.pdf]

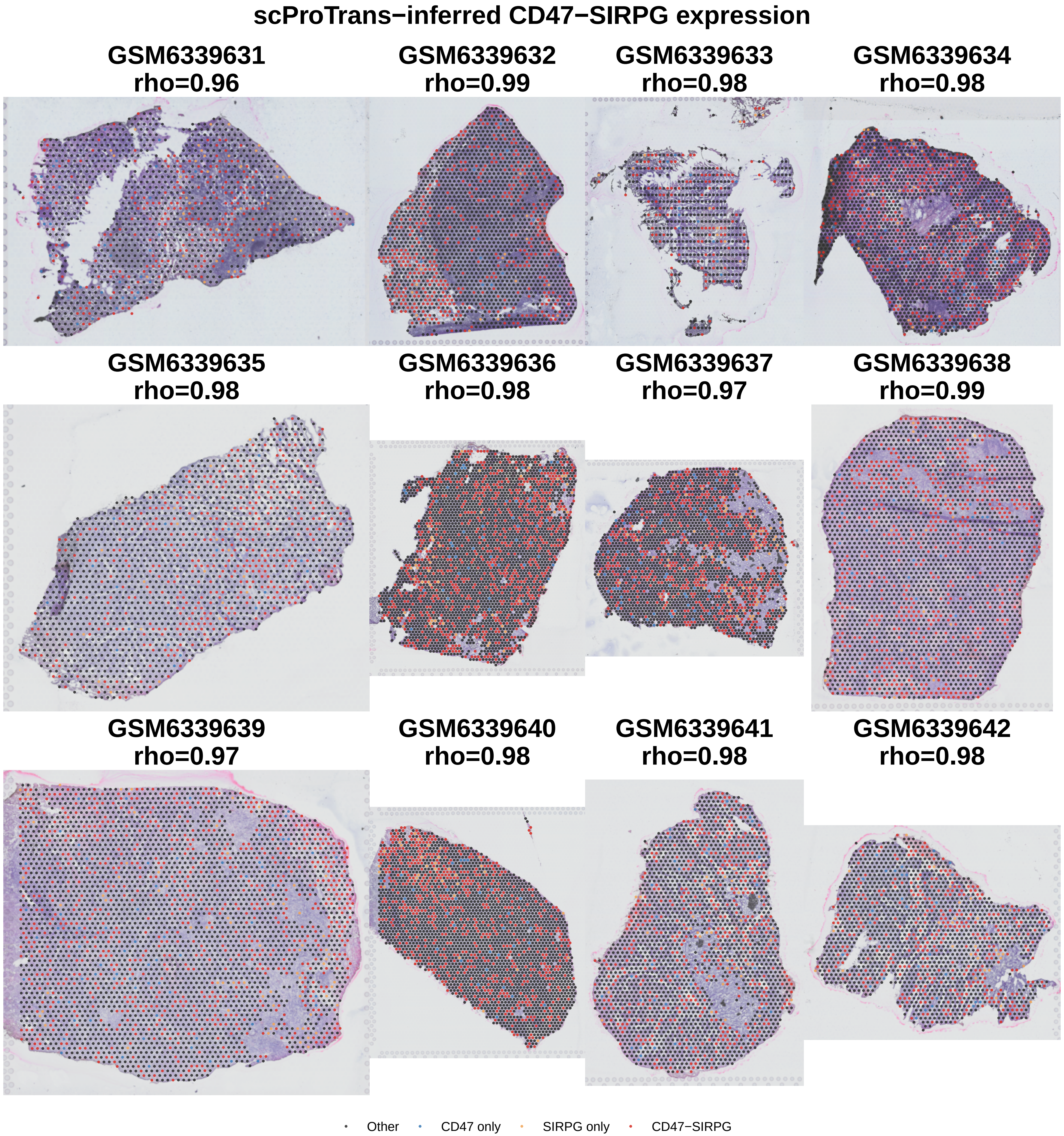
scProTrans−inferred CD47−SIRPG expression
GSM6339631
rho=0.96
GSM6339632
rho=0.99
GSM6339633
rho=0.98
GSM6339634
rho=0.98
GSM6339635
rho=0.98
GSM6339636
rho=0.98
GSM6339637
rho=0.97
GSM6339638
rho=0.99
GSM6339639
rho=0.97
GSM6339640
rho=0.98
GSM6339641
rho=0.98
GSM6339642
rho=0.98
Other
CD47 only
SIRPG only
CD47−SIRPG
